# Evaluating long-read *de novo* assembly tools for eukaryotic genomes: insights and considerations

**DOI:** 10.1093/gigascience/giad100

**Published:** 2023-11-24

**Authors:** Bianca-Maria Cosma, Ramin Shirali Hossein Zade, Erin Noel Jordan, Paul van Lent, Chengyao Peng, Stephanie Pillay, Thomas Abeel

**Affiliations:** Delft Bioinformatics Lab, Intelligent Systems, Delft University of Technology, 2628 XE, Delft, The Netherlands; Delft Bioinformatics Lab, Intelligent Systems, Delft University of Technology, 2628 XE, Delft, The Netherlands; Delft Bioinformatics Lab, Intelligent Systems, Delft University of Technology, 2628 XE, Delft, The Netherlands; Technical Biochemistry, TU Dortmund University, 44227, Dortmund, Germany; Delft Bioinformatics Lab, Intelligent Systems, Delft University of Technology, 2628 XE, Delft, The Netherlands; Delft Bioinformatics Lab, Intelligent Systems, Delft University of Technology, 2628 XE, Delft, The Netherlands; Delft Bioinformatics Lab, Intelligent Systems, Delft University of Technology, 2628 XE, Delft, The Netherlands; Delft Bioinformatics Lab, Intelligent Systems, Delft University of Technology, 2628 XE, Delft, The Netherlands; Infectious Disease and Microbiome Program, Broad Institute of MIT and Harvard, Cambridge, MA 02142, USA

**Keywords:** *de novo* assembly, third-generation sequencing, benchmarking, eukaryote genomes

## Abstract

**Background:**

Assembly algorithm choice should be a deliberate, well-justified decision when researchers create genome assemblies for eukaryotic organisms from third-generation sequencing technologies. While third-generation sequencing by Oxford Nanopore Technologies (ONT) and Pacific Biosciences (PacBio) has overcome the disadvantages of short read lengths specific to next-generation sequencing (NGS), third-generation sequencers are known to produce more error-prone reads, thereby generating a new set of challenges for assembly algorithms and pipelines. However, the introduction of HiFi reads, which offer substantially reduced error rates, has provided a promising solution for more accurate assembly outcomes. Since the introduction of third-generation sequencing technologies, many tools have been developed that aim to take advantage of the longer reads, and researchers need to choose the correct assembler for their projects.

**Results:**

We benchmarked state-of-the-art long-read *de novo* assemblers to help readers make a balanced choice for the assembly of eukaryotes. To this end, we used 12 real and 64 simulated datasets from different eukaryotic genomes, with different read length distributions, imitating PacBio continuous long-read (CLR), PacBio high-fidelity (HiFi), and ONT sequencing to evaluate the assemblers. We include 5 commonly used long-read assemblers in our benchmark: Canu, Flye, Miniasm, Raven, and wtdbg2 for ONT and PacBio CLR reads. For PacBio HiFi reads , we include 5 state-of-the-art HiFi assemblers: HiCanu, Flye, Hifiasm, LJA, and MBG. Evaluation categories address the following metrics: reference-based metrics, assembly statistics, misassembly count, BUSCO completeness, runtime, and RAM usage. Additionally, we investigated the effect of increased read length on the quality of the assemblies and report that read length can, but does not always, positively impact assembly quality.

**Conclusions:**

Our benchmark concludes that there is no assembler that performs the best in all the evaluation categories. However, our results show that overall Flye is the best-performing assembler for PacBio CLR and ONT reads, both on real and simulated data. Meanwhile, best-performing PacBio HiFi assemblers are Hifiasm and LJA. Next, the benchmarking using longer reads shows that the increased read length improves assembly quality, but the extent to which that can be achieved depends on the size and complexity of the reference genome.

## Introduction

*De novo* genome assembly is essential in several leading fields of research, including disease identification, gene identification, and evolutionary biology [1–4]. Unlike reference-based assembly, which relies on the use of a reference genome, *de novo* assembly only uses the genomic information contained within the sequenced reads. Since it is not constrained to the use of a reference, high-quality *de novo* assembly is essential for studying novel organisms, as well as for the discovery of overlooked genomic features, such as gene duplication [5], in previously assembled genomes.

The introduction of third-generation sequencing (TGS) led to massive improvements in *de novo* assembly. The advent of TGS has addressed the main drawback of next-generation sequencing (NGS) platforms—namely, the short read length—but has introduced new challenges in genome assembly, because of the higher error rates of long reads. The leading platforms in long-read sequencing are Pacific Biosciences Single Molecule, Real-Time sequencing (often abbreviated as “PacBio”) and Oxford Nanopore (ONT) sequencing [6].

Since the introduction of TGS platforms, many methods have been developed that aim to take the most benefits from the longer read length and overcome the new challenges due to sequencing error. Recent studies have been conducted to compare long-read *de novo* assemblers. One such study was conducted by Wick and Holt [7], who focused on long-read *de novo* assembly of prokaryotic genomes. Eight assemblers were tested on real and simulated reads from PacBio and ONT sequencing, and evaluation metrics included sequence identities, circularization of contigs, computational resources, and accuracy. Murigneux et al. [8] performed similar experiments on the genome of *Macadamia jansenii*, although in this case, the focus was on comparatively benchmarking Illumina sequencing and 3 long-read sequencing technologies, in addition to the comparison of long-read assembly tools. Studies narrowed down to just 1 type of sequencing technology include those of Jung et al. [9], who evaluated assemblers on real PacBio reads from 5 plant genomes, and Chen et al. [10], who used Oxford Nanopore real and simulated reads from bacterial pathogens in their comparison. Except for the Wick and Holt study, which provides a compressive comparison on *de novo* assembly of prokaryotic genomes, other studies are either comparing the assemblers on single genome or using data from a single sequencing platform. Here, we provide a comprehensive comparison on *de novo* assembly tools on the most used TGS technologies and 7 different eukaryotic genomes, to complement the study of Wick and Holt.

**Figure 1:**
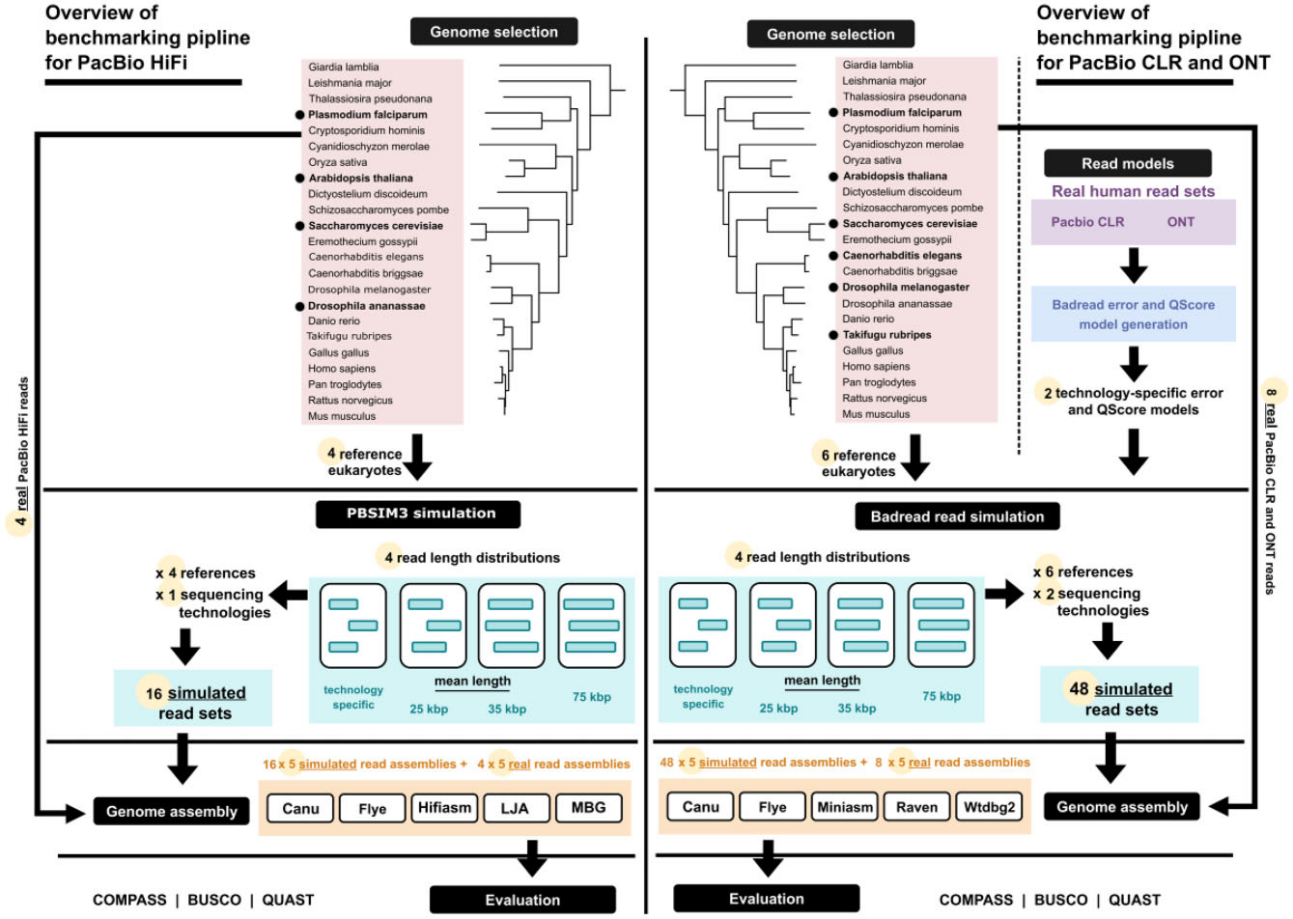
The benchmarking pipeline. For PacBio CLR and ONT (right panel), first we select 6 representative eukaryotes from the Tree of Life [11] and use Badread's error and Qscore model generation feature [15] to create 2 models of PacBio CLR and ONT long sequencing technologies. This is input to the read simulation stage, where we simulate reads from all genomes, with 4 different read length distributions. We then perform assembly of simulated and real reads, using 5 long-read assemblers. For PacBio HiFi (left panel), first we select 4 representative eukaryotes and use PBSIM3 to simulate HiFi reads. These reads are then assembled using 5 state-of-the-art HiFi assemblers. Lastly, we evaluate all PacBio HiFi, PacBio CLR, and ONT assemblies based on several criteria.

In this study, we are benchmarking these methods using 12 real and 64 simulated datasets (see Fig. 1) from PacBio continuous long-read (CLR), PacBio high-fidelity (HiFi), and ONT platforms to guide researchers to choose the proper assembler for their studies. Benchmarking using simulated reads allows us to accurately compare the final assembly with the ground truth, and benchmarking using the real reads can validate the results based on simulated reads. The assembler comparison presented in this article complements the literature that has already been published, by introducing an analysis of not just assembler performance but also of the effect of read length on assembly quality. Although increased read length is considered an advantage, we investigate if it is always a necessary advantage to have for assembly performance. To that end, the scope of the study extends to 6 model eukaryotes that provide a performance indication for genomes of variable complexity, covering a wide range of taxa on the eukaryotic branch of the Tree of Life [11]. Complexity in genome assembly is determined by multiple variables, the most notable of which is the proportion of repetitive sequences within the genome of a particular organism. Complexity in eukaryotic genomes is further exacerbated by size and organization of chromosomal architecture, including telomeres and centromeres, and the presence of circular elements such as mitochondrial and chloroplast DNA.

*De novo* genome assembly evaluation remains challenging, as it represents a process that must account for variables such as the goal of an assembly and the existence of a ground-truth reference. A standard evaluation procedure was introduced in the literature by the 2 Assemblathon competitions [12, 13], which outlined a selection of metrics that encompasses the most relevant aspects of genome assembly, but these metrics require a reference sequence. Most of these metrics are adopted in our benchmark.

Consequently, this study addresses 2 main objectives. First, we provide a systematic comparison of state-of-the-art long-read assembly tools, documenting their performance in assembling real and simulated PacBio CLRs, PacBio HiFi reads, and ONT reads on a diverse set of eukaryotic organisms. The PacBio CLR and ONT reads generated from the genomes of *Saccharomyces cerevisiae, Plasmodium falciparum, Caenorhabditis elegans, Arabidopsis thaliana, Drosophila melanogaster*, and *Takifugu rubripes* and the PacBio HiFi reads are generated from the genomes of *S. cerevisiae, P. falciparum, A. thaliana*, and *Drosophila ananassae*. Our second objective is to investigate whether increased read length has a positive effect on overall assembly quality, given that increasing the length of reads is an ongoing effort in the development of TGS platforms [14].

It is important to note that our objective is to evaluate the performance of these tools in generating a consensus assembly without taking haplotypes into account. Moreover, it is crucial to highlight that the results and conclusions drawn from this comparison may not be directly applicable to metagenome assembly. The unique characteristics and complexities associated with metagenomic data warrant a separate and distinct analysis, which is beyond the scope of this study.

## Materials and Methods

### Data

In this study, we are using real and simulated data from various organisms to benchmark long-read *de novo* assembly tools.

#### Reference genomes

We selected 6 reference genomes from eukaryotic organisms represented in the Interactive Tree of Life (iTOL) v6 [11] for evaluating PacBio CLR and ONT assemblers: *S. cerevisiae* (strain S288C), *P. falciparum* (isolate 3D7), *C. elegans* (strain VC2010), *A. thaliana* (ecotype Col-0), *D. melanogaster* (strain ISO-1), and *T. rubripes*. Moreover, we selected the 4 eukaryotic organisms to evaluate PacBio HiFi assemblers: *S. cerevisiae* (strain S288C), *P. falciparum* (isolate 3D7), *A. thaliana* (ecotype Col-0), and *D. ananassae* (strain 14024–0371.13). Assembly accessions are included in Supplementary Table S1.

The reference assemblies for *C. elegans, D. melanogaster*, and *T. rubripes* included uncalled bases. In these cases, before read simulation, each base N was replaced with base A, as done by Wick and Holt [7]. This avoids ambiguity in the read simulation process and consequently simplifies the evaluation of the simulated read assemblies. As such, we used this modified version as a reference when evaluating all assemblies of simulated reads from these 4 genomes. In the evaluation of real read assemblies, the original assemblies were used as references.

#### Simulated reads

The PacBio CLR and ONT simulated read sets were generated using Badread v0.2.0 [15]. To create read error and Qscore (quality score) models in addition to the simulator's own default models, Badread requires the following 3 parameters: a set of real reads, a high-quality reference genome, and an alignment file, obtained by aligning the reads to the reference genome. We used real read sets from the human genome to create error and Qscore models that reflect the state of the art for PacBio CLRs and Oxford Nanopore reads. The simulated PacBio HiFi reads were generated using PBSIM3. To generate reads similar to HiFi, we used the –num-pass 10 parameter and then applied ccs version 6.4.0 to generate the consensus reads.

To create the models for PacBio CLR and Oxford Nanopore reads, we used the real read sets sequenced from the human genome and aligned to the latest high-quality human genome reference assembled by [16]: assembly T2T-CHM13v2.0, with RefSeq accession GCF_009914755.1. The alignment was performed using Minimap2 v2.24 (RRID:SCR_018550) [17] with default parameters. The sources for these sequencing data are outlined in Supplementary Table S2, as well as the read identities for each technology, which are later passed as parameters for the simulation stage.

To study the effect of read length on genome assembly, we simulated reads that imitate PacBio CLR, PacBio HiFi, and Oxford Nanopore sequencing, with 4 different read length distributions, using Badread for PacBio CLR and Oxford Nanopore sequencing while using PBSIM3 for PacBio HiFi. The first read simulation represents the current state of the 3 long-read technologies. The other 3 simulations reflect data points in between technology-specific values and ultra-long reads, data points of a similar length as ultra-long-reads, and longer than ultra-long reads. We need to define the mean and standard deviation of the read length distributions for these simulations. The values for the mean and standard deviation of these distributions were selected as follows. First, we calculated the read length distributions of the real read sets in Supplementary Table S2 and simulated an initial iteration of reads using these technology-specific values. For choosing these values for the other 3 iterations, we analyzed a set of Oxford Nanopore ultra-long reads used in the latest assembly of the human genome [16]. We selected GridION run SRR12564452, available as sequence data in BioProject PRJNA559484, with a mean read length of approximately 35.7 kilobase pairs (kbp) and a standard deviation of 42.5 kbp. A summary of the Badread and PBSIM3 commands used in our simulation can be found in Supplementary Tables S3 and S4.

A full overview of the mean and standard deviation of all 4 read length distributions is given in Table 1. Note that, for each of the technologies, the standard deviation for the last 3 distributions was derived from the mean, using the ratio between the mean and standard deviation reflected by the technology-specific values. Hence, for the last 3 iterations, the mean read length is consistent across sequencing technologies, but the standard deviation varies.

**Table 1:** The mean and standard deviation describing the read length distributions used in our simulations. Note that read length increases with each iteration, and the distribution parameters are different for each technology.

	Read length distribution parameters (kbp), per technology					
	PacBio CLR		PacBio HiFi		Oxford Nanopore	
	Mean	SD	Mean	SD	Mean	SD
**Iteration 1** (technology-specific values)	15.7	14.4	20.7	2.5	12.1	17.1
**Iteration 2**	25	22.5	25	3	25	35
**Iteration 3** (imitate ultra-long reads)	35	31.5	35	4.2	35	49
**Iteration 4**	75	67.5	75	9	75	105

Consequently, we ran the simulations for each reference genome. As described above, we used our own models for each technology and passed them to the simulator as the –error_model and –qscore_model. The read identities per technology were set to the values included in Supplementary Table S3. Across all simulations, we chose a coverage depth of 30×. Canu’s documentation [18] specifies a minimum coverage of 20–25× for HiFi data and 20× for other types of data, while Flye’s guidelines [19] indicate a minimum coverage of 30×. As there is no minimum recommended coverage indicated for the other assemblers we used in our benchmark, we simulated reads following the stricter guideline among these two, that is, 30× coverage.

#### Real reads

In support of our evaluation on simulated reads, we also performed a benchmark on real read assemblies from Oxford Nanopore and PacBio reads sequenced from the reference genomes. These reads were sampled to approximately 30× coverage, to avoid introducing potentially confounding variables when comparing assemblies of real and simulated datasets. The data sources for all real sets are included in Supplementary Table S5. Please note that the PacBio CLR data from *C. elegans* were generated using the older RSII technology. These reads’ inherent characteristics of the RSII system, such as shorter average reads and a higher error rate, might have influenced the assembly results.

### Assemblies

For the PacBio CLR and ONT reads, we included the following 5 long-read *de novo* assemblers: Canu v2.2 (RRID:SCR_015880) [18], Flye v2.9 (RRID:SCR_017016) [19], Wtdbg2 (also known as Redbean) v2.5 (RRID:SCR_017225) [20], Raven v1.7.0 (RRID:SCR_001937) [21], and Miniasm v0.3_r179 (RRID:SCR_024114) [22]. For the PacBio HiFi reads, we included HiCanu v2.2 [23], Flye v2.9, Hifiasm 0.19.5-r587 [24], LJA v 0.2 [25], and MBG v 1.0.14 [26]. We used the most recent releases of the assemblers at the time we started this study.

The assemblies were performed with default values for most parameters. Canu and Wtdbg2 require the estimated genome size as a parameter, and we set the following values: *S. cerevisiae* = 12 megabase pairs (Mbp), *P. falciparum* = 23 Mbp, *A. thaliana* = 135 Mbp, *D. melanogaster* = 139 Mbp, *C. elegans* = 103 Mbp, *T. rubripes* = 384 Mbp, and *D. ananassae* = 217 Mbp. All commands used in the assembly pipelines are available in Supplementary Table S6. We note that further polishing of assemblies using high-fidelity short reads, although common in practice [27–29], is omitted in this study, as the focus is exclusively on assembler performance on long-read data and not polishing tools.

We added a long-read polishing step for Miniasm and Wtdbg2, as their assembly pipelines do not include long-read based polishing. Following Raven’s default pipeline, which performs 2 rounds of Racon polishing [30], we used 2 rounds of Racon polishing on Wtdbg2 and Miniasm. We note that for Miniasm, we used Minipolish [7], which simplifies Racon polishing by applying it in 2 iterations on the Graphical Fragment Assembly files produced by the assembler. For both Miniasm and Wtdbg2, the alignments required for polishing were generated with Minimap v2.24.

### Evaluation

We evaluated the assemblies in 3 different categories of metrics. The COMPASS analysis compares the assemblies with their corresponding reference genome and provides insight into their similarities. The assembly statistics provide some basic knowledge about the contiguity and misassemblies. Finally, the BUSCO assessment investigates the presence of essential genes in the assemblies. These 3 categories of metrics, next to each other, can provide a complete overview of the assembly’s quality.

#### Correctness analysis

For each assembly, we ran the COMPASS script to measure the coverage, validity, multiplicity, and parsimony, to assess the quality of the assemblies, as defined in Assemblathon 2 [13]. These metrics describe several characteristics that were deemed important for comparing *de novo* assembly tools, and they were computed using 3 types of data: (i) the reference sequence, (ii) the assembled scaffolds, and (iii) the alignments (sequences from the assembled scaffolds that were aligned to the reference sequences). Definitions and formulas for the metrics are reported in Supplementary Table S7.

Moreover, we use QUAST v5.2.0 (RRID:SCR_001228) [31] to calculate the number of misassemblies. QUAST identifies misassemblies based on the definition outlined by [32]. The total number of misassemblies is the sum of all relocations, inversions, and translocations. Considering 2 adjacent flanking sequences, if they both align to the same chromosome, but 1 kbp away from each other, or overlapping for more than 1 kbp, this is counted as a relocation. If these flanking sequences, aligned to the same chromosome, are on opposite strands, the misassembly is considered an inversion. Lastly, translocations describe events in which 2 flanking sequences align to different chromosomes.

#### Contiguity assessment

We use QUAST v5.2.0 [31] to measure the auNGA of an assembly. The auNGA metric, standing for the area under the NGAx [12] curve, is a measure of assembly contiguity. By calculating the area beneath this profile, which integrates the aligned sequence fragment or contig lengths at various percentage thresholds, it provides a more thorough understanding of the contiguity of the assembly compared to single-value metrics. A larger auNGA value indicates better contiguity in the genome assembly.

#### Completeness assessment

BUSCO v5.4.2 (RRID:SCR_015008) assessment [33, 34] is performed to evaluate the completeness of the essential genes in the assemblies. This quantifies the number of single-copy, duplicated, fragmented, and missing orthologs in an assembled genome. From the number of orthologs specific to each dataset, BUSCO identifies how many orthologs are present in the assembly (either as single copy or duplicated), how many are fragmented, and how many are missing.  We ran these evaluations with a different OrthoDB lineage dataset for each genome: *S. cerevisiae*—saccharomycetes, *P. falciparum—*plasmodium, *A. thaliana—*brassicales, *D. melanogaster*—diptera, *C. elegans*—nematoda, *T. rubripes*—ctinopterygii, and *D. ananassae*—diptera.

## Results and Discussion

### Overview of the benchmarking pipeline

Figure 1 shows an overview of the benchmarking pipeline. For the PacBio CLR and Oxford Nanopore reads, we begin with the selection of 6 representative eukaryotes from the iTOL [11]: *S. cerevisiae, P. falciparum, A. thaliana, D. melanogaster, C. elegans*, and *T. rubripes*. We also use 3 read sets from the latest human assembly project [16] to generate Badread error and Qscore models [15] for PacBio CLRs and Oxford Nanopore reads (see Supplementary Table S2). The reference sequences and models become input to the Badread simulation stage. For each genome, we simulate reads with 4 different read length distributions and 2 sequencing technologies (see Table 1), amounting to a total of 8 simulated read sets per reference genome. These reads, as well as real read sets, are assembled with 5 assembly tools: Canu, Flye, Miniasm, Raven, and Wtdbg2.

For the PacBio HiFi reads, we begin with the reference genome of the 4 selected eukaryote species: *S. cerevisiae, P. falciparum, A. thaliana*, and *D. ananassae*. Then we use PBSIM3 and CCS to generate PacBio HiFi reads. Similar to the previous setup, for each reference genome, we simulate reads with 4 different read length distributions. The simulated reads along with real reads for each of the 4 reference genomes are assembled with 5 assembly tools: HiCanu, Flye, Hifiasm, LJA, and MBG.

Next, the resulting assemblies are evaluated using COMPASS, QUAST, and BUSCO, and based on the reported metrics, we distinguish 6 main evaluation categories: sequence identity, repeat collapse, rate of valid sequences, contiguity, misassembly count, and gene identification. The selected COMPASS metrics are the coverage, multiplicity, and validity of an assembly, which provide insight on sequence identity, repeat collapse, and the rate of valid sequences, respectively. In this regard, an ideal assembly has coverage, multiplicity, and validity close to 1. This suggests that a large fraction of the reference genome is assembled, repeats are generally collapsed instead of replicated, and most sequences in the assembly are validated by the reference. Among others, QUAST reports the number of misassemblies and the auNG of an assembly. A high auNG value indicates high contiguity. In order to assess contiguity across genomes of different sizes, we report the ratio between the assembly’s auNG and the N50 of the references. Lastly, gene identification is quantified in terms of the percentage of complete BUSCOs in an assembly.

### The search for an optimal assembler for PacBio CLR and ONT reads is influenced by read sequencing technology, genome complexity, and research goal

To select an assembler that is most versatile across eukaryotic taxa, we simulate PacBio CLRs and Oxford Nanopore reads from the genomes of 6 eukaryotes, assemble these reads, and evaluate the assemblers in the 6 main categories mentioned in the previous section. The results for each evaluation category are normalized in the range given by the worst and best values encountered in the evaluation of all assemblies of reads with default length. This highlights differences between assemblers, as well as between genomes and sequencing technologies.

**Figure 2:**
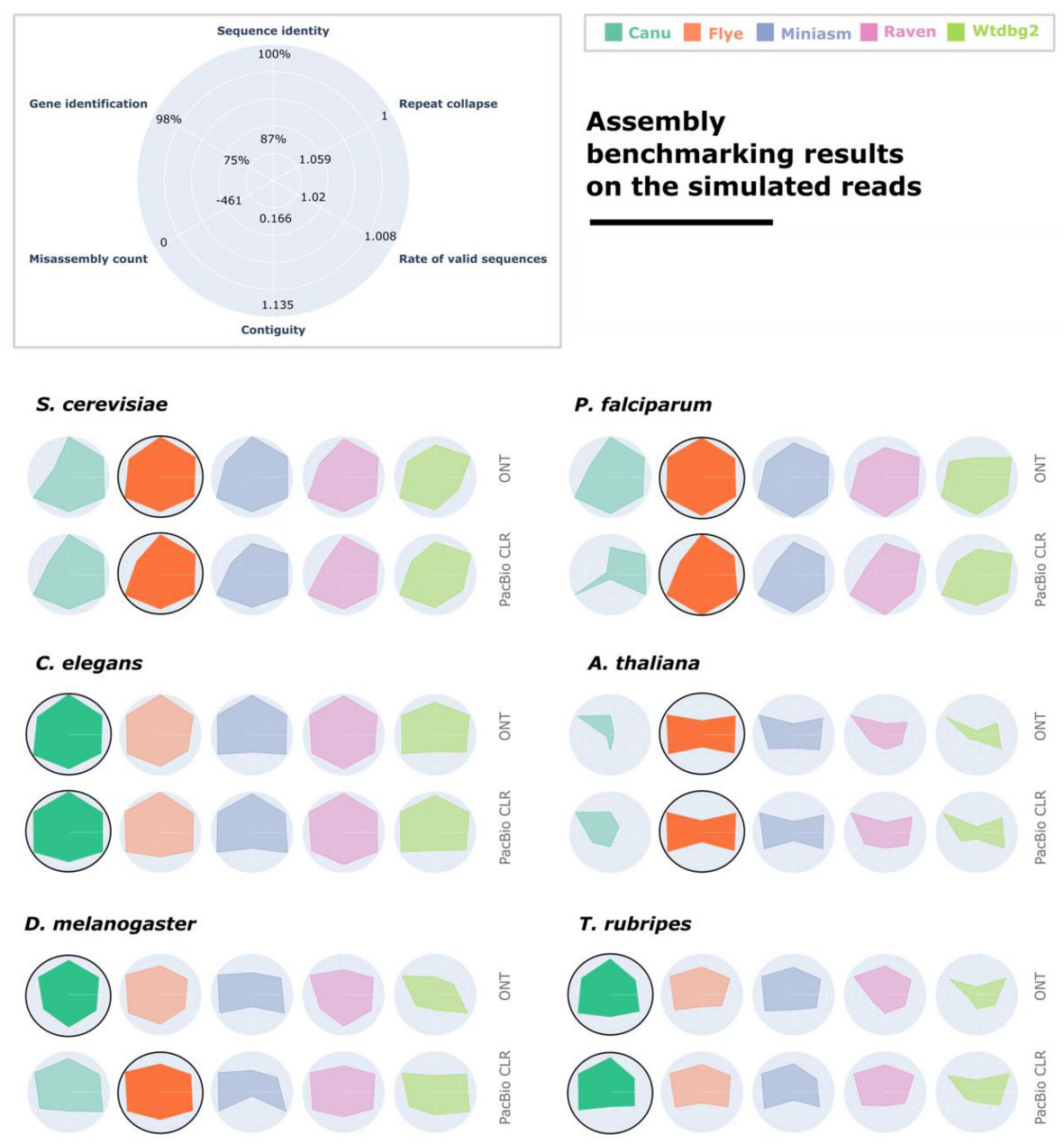
The performance of the 5 assemblers on the read sets with default read lengths, from iteration 1 (see Table 1), generated from 6 eukaryotic genomes. Six evaluation categories are reported for each assembler, and the results are normalized among all assemblies included in the figure. Ranges for each metric are reported as the best and worst values computed for these assemblies. The best-performing assembler is highlighted and has a black outline.

The results of the benchmark on the PacBio CLR and ONT read sets with default lengths—namely, those belonging to the first iteration (see Table 1)—are illustrated in Fig. 2. A full report of the evaluation metrics in this figure is included in the Supplementary Tables S8–S24, under “Iteration 1.” We note that no assembler unanimously ranks first in all categories, across different sequencing technologies and eukaryotic genomes, although our findings highlight some of their strengths and thus their potential for various research aims. The runtime and memory usage of the assembly tools on all of the simulated datasets are reported in Supplementary Tables S25–S30, since this can also be a deciding factor next to the quality of the assembly for the researchers to choose the suitable assembler for their purpose. We note that all assemblies were run on our local High-Performance Computing Cluster, and the runtime and RAM usage may have been affected by the heterogeneity of the shared computing environment in which the assembly jobs executed.

While working with PacBio CLR and ONT reads, Miniasm, Raven, and Wtdbg2 are all well-rounded choices for the simpler *S. cerevisiae, P. falciparum*, and *C. elegans* genomes, with a balanced trade-off between assembly quality and computational resources. For PacBio HiFi reads, Raven is generally qualitatively outperformed by other assemblers like Canu, Flye, and Miniasm, likely as a consequence of the fact that its pipeline is not customized for all long-read sequencing technology. Nonetheless, if computational resources are a concern, Raven is a more suitable choice, since Miniasm and Wtdbg2 do not scale well for larger genomes.

We can single out Flye as the most robust assembler for PacBio CLR and ONT reads across all 6 organisms, although for larger genomes such as *T. rubripes*, Canu is a better tool. Both produce assemblies with high sequence identity and validity, as well as good gene prediction, but Flye assemblies generally rank first when we compute the average score across all 6 metrics. For Canu, we notice more variation in assembly quality across different genomes, particularly for *P. falciparum* and *A. thaliana*, while Flye maintains more consistent results. Nonetheless, on the *T. rubripes* genome, Canu assemblies have higher sequence identity and contiguity, as well as more accurate gene identification.

### Evaluation of PacBio CLR and ONT real read assemblies supports our rankings on simulated read assemblies

**Figure 3:**
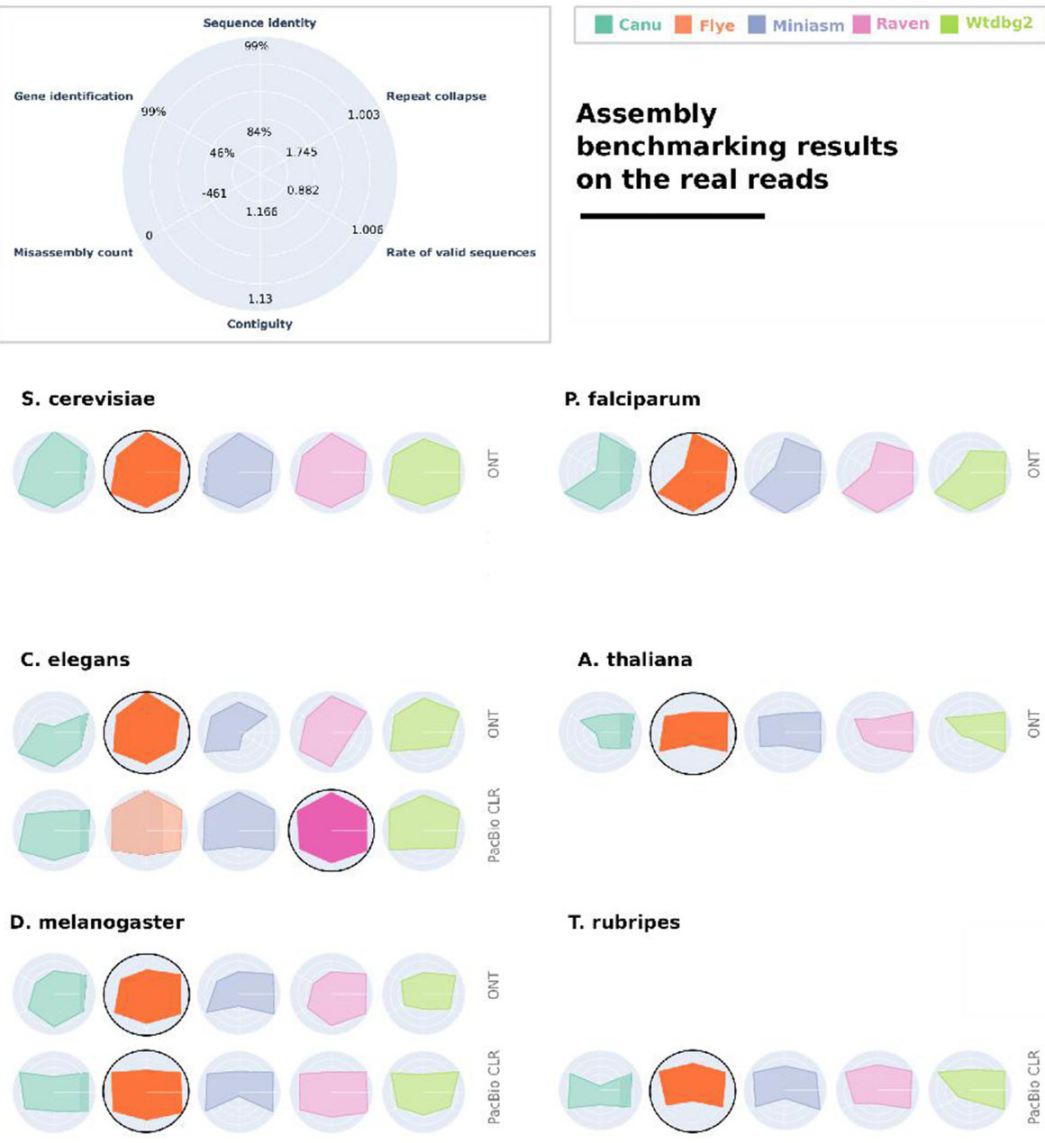
The performance of the 5 assemblers on the real PacBio CLR and ONT reads (see Supplementary Table S5), sequenced from 6 eukaryotic genomes. As in Fig. 2, 6 evaluation categories are reported for each assembler, and the results are normalized among all assemblies included in the figure. Ranges for each metric are reported as the best and worst values computed for these assemblies. The best-performing assembler is highlighted and has a black outline.

To determine assembler performance on real PacBio CLR and ONT reads and validate the rankings of the simulated read assemblies, we assemble several real read sets from the 6 reference eukaryotes (Supplementary Table S5). Supplementary Figs. S1–S12 provide a visual representation of the read length distribution for all of the real read sets. The evaluation results on the real read assemblies, summarized in Fig. 3, indicate that assemblers that perform well on simulated reads perform similarly well in assembling the sets of real reads. The full report of metrics on the real read assemblies is included in Supplementary Table S31. We conclude that, overall, the assembler rankings remain consistent. This illustrates that benchmarking using simulated data is similar to real read sets. For reference-based metrics, we used the reference genomes given in Supplementary Table S1.

Notably, reference-based metrics in the evaluation of real read assemblies rely on comparisons with an assembly and not the genome from which the reads were initially sequenced. In contrast to the evaluation of simulated read assemblies, the existence of a ground-truth reference is not available in this case, but reference-based metrics are included for the sake of consistency with the simulated read evaluation.

In the evaluation of real read assemblies of PacBio CLR and ONT reads, Flye ranks first for nearly all datasets, with the exception of the *T. rubripes* and *C. elegans* PacBio reads, for which Raven performs better overall. However, even in *C. elegans*, Flye performance is close to the best values in all metrics other than contiguity. As expected, overall assembler performance decreases for reference-based metrics like sequence identity, repeat collapse, and validity, but surprisingly the misassembly count is considerably lower.

### Searching for the best HiFi assembler based on simulated and real datasets

Similarly, in order to identify the best-performing HiFi assembler for diverse eukaryotic taxa, we first generate simulated PacBio HiFi reads from the genomes of 4 different eukaryotes. These simulated reads are then assembled, and the performance of each assembler is evaluated based on the 6 primary categories outlined in the previous section. For comparative clarity, the results for each evaluation category are normalized within the range established by the lowest and highest values observed across all assembly evaluations of reads of default length. This method emphasizes both the variations among different assemblers, as well as the discrepancies across genomes and sequencing technologies.

**Figure 4:**
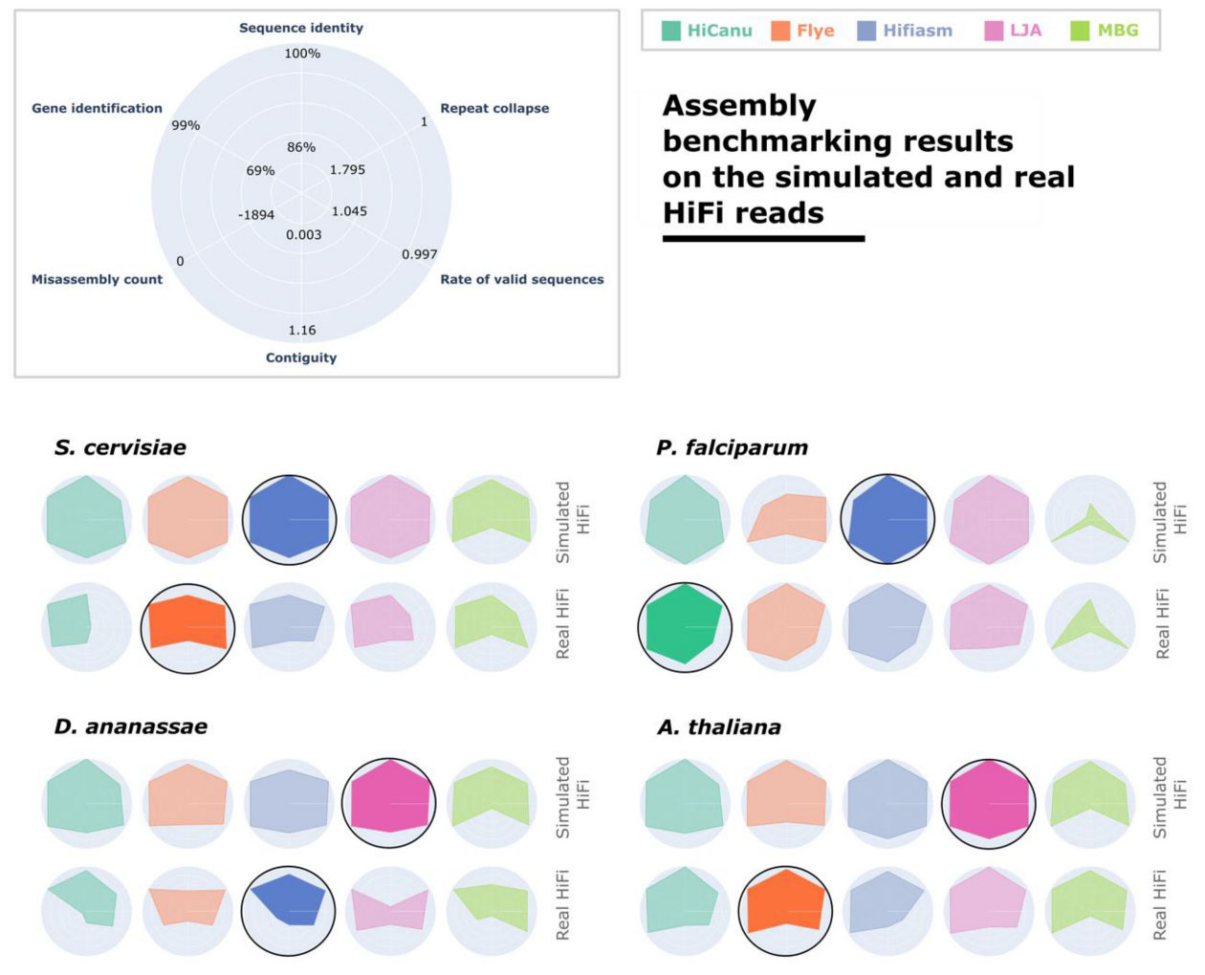
The performance of the 5 assemblers on the real PacBio HiFi read sets and simulated PacBio HiFi read sets with default read lengths, from iteration 1 (see Table 1), generated from 4 eukaryotic genomes. Six evaluation categories are reported for each assembler, and the results are normalized among all assemblies included in the figure. Ranges for each metric are reported as the best and worst values computed for these assemblies. The best-performing assembler is highlighted and has a black outline.

The results from simulated PacBio HiFi read sets with default lengths—namely, those belonging to the first iteration (see Table 1)—are illustrated in Fig. 4. Next to that, the results of real HiFi reads of the same species are presented in Fig. 4. We note that Hifiasm and LJA outperformed other assemblers and performed well in all datasets. The assembly results generated by the MBG assembler demonstrated notably low sequence identity when compared to the reference genome.

### Longer reads lead to more contiguous assemblies of large genomes but do not always improve assembly quality

To investigate the effect of increased read length on assembly quality, we simulate Oxford Nanopore, as well as PacBio CLR and HiFi reads with different read length distributions (Table 1). These reads are simulated from the genomes of *S. cerevisiae, P. falciparum, C. elegans, A. thaliana, D. melanogaster*, and *T. rubripes* for PacBio CLR and ONT reads, as well as *S. cerevisiae, P. falciparum, A. thaliana*, and *D. ananassae* for PacBio HiFi reads. We assemble PacBio CLR and ONT reads with Canu, Flye, wtdbg2, Raven, and miniasm and assemble PacBio HiFi reads with HiCanu, Flye, Hifiasm, LJA, and MBG. We evaluate assembly quality based on 6 evaluation categories (see Overview of the benchmarking pipeline). It is worth mentioning that Canu’s PacBio CLR and ONT reads iteration 4 (the longest reads) assemblies of *A. thaliana* and *T. rubripes* did not finish within reasonable time and are excluded from the evaluation.

**Figure 5:**
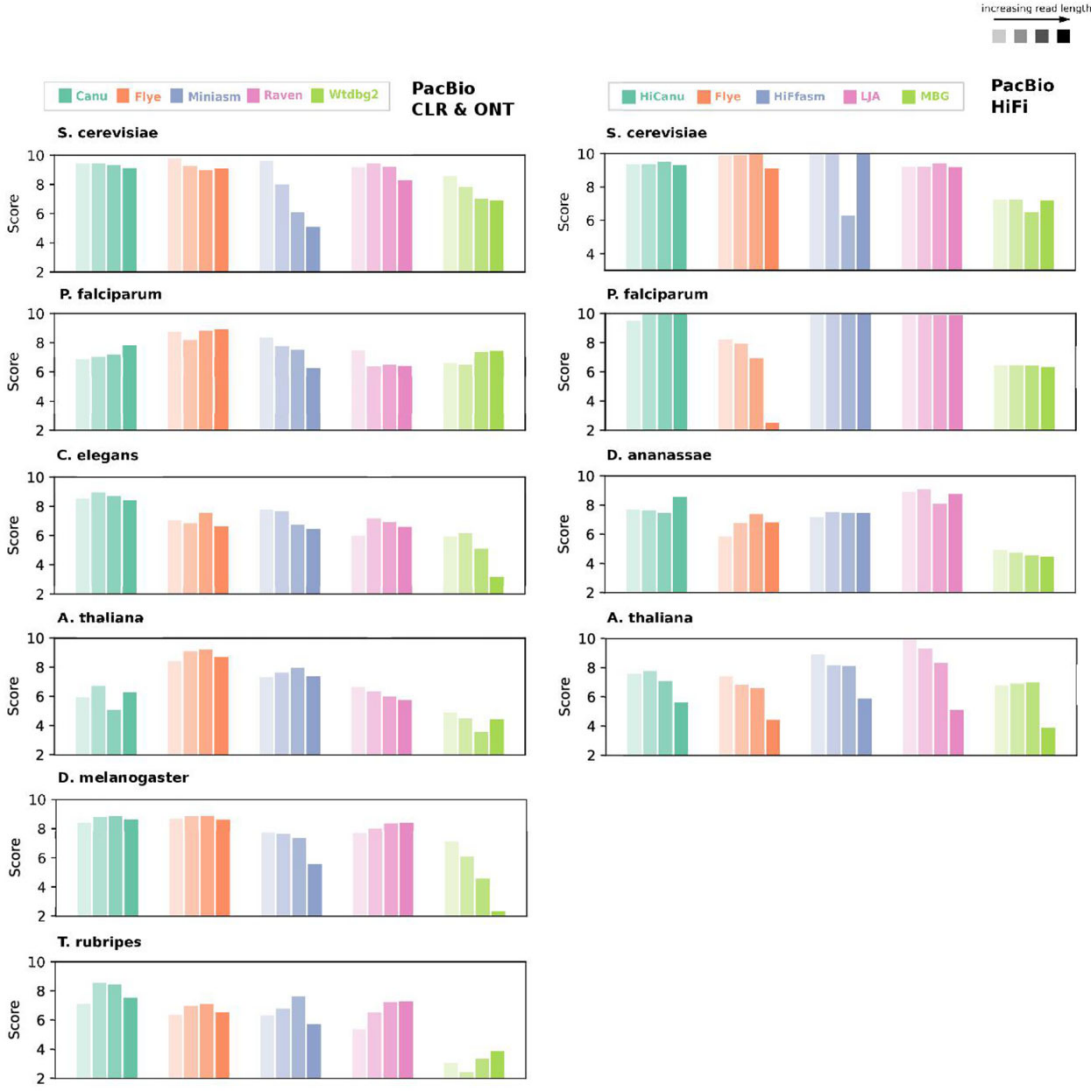
The left panel shows the performance of the 5 assemblers on all simulated PacBio CLR and ONT read sets, with 4 different read length distributions (as previously described in Table 1). A score of 1–10 is reported for each assembler. We did not divide the auNGA with the N50 of the reference genomes for this figure. The results are normalized for each genome, per sequencing technology. For PacBio CLR and ONT, an average score for each read length distribution is first computed and then these 2 scores are averaged to obtain an overall score per read length distribution. For the *A. thaliana* and *T. rubripes* ONT iteration 4, the Canu assembly was not completed. Therefore, the iteration 4 bar in the plot represents only the PacBio CLR assemblies. Similarly, the right panel shows the performance of the 5 HiFi assemblers on all simulated PacBio HiFi read sets with 4 different read length distributions.

Figure 5 shows a summary of the assemblers’ performance on all simulated read sets, highlighting changes in performance for each read length distribution. All 6 evaluation metrics are normalized given the maximum and minimum metric values per genome, per sequencing technology, and combined to obtain an average score. For PacBio CLR and ONT read sets, we then average the 2 resulted scores. Finally, we report a rate between 1 and 10 for each assembler, per read length distribution for PacBio CLR and ONT read sets, and a separate score for PacBio HiFi read sets. The results on all computed metrics are fully described in Supplementary Tables S8–S24.

The results imply that there is a correlation between the size and complexity of the reference genome and the extent of the improvement in assembly quality that can be achieved by increasing the length of the reads. While we observe no trend in assembly quality improvement on the assemblies of smaller genomes, the results on the *T. rubripes* assemblies are more conclusively in favor of the longer reads. For instance, on the shorter and simpler *S. cerevisiae* and *P. falciparum* genomes, identification of repetitive and complex regions is not aided by increased read length, likely as these regions are already spanned by the reads with default lengths. However, the benchmark results suggest that more complex and repetitive regions within the *A. thaliana, D. melanogaster*, and, most notably, *T. rubripes* genomes are better captured by longer reads.

As recorded in Supplementary Tables S22 and S23, for larger genomes, longer reads generally lead to significantly higher assembly contiguity and a lower misassembly count. The latter implies that the resulting assemblies are more faithful to the references, although this is not necessarily supported by other metrics. We cannot report any compelling improvements in sequence identity, multiplicity, validity, and gene identification.

## Conclusion

In fulfillment of the first objective of this study, we conclude that Flye is the highest-performing assembler when considering the overview of all evaluation categories in this benchmark, which include the sequence identity, repeat collapse, rate of valid sequences, contiguity, misassembly count, and gene identification. Rankings are mostly consistent for all 3 sequencing platforms included in the study: PacBio CLR, PacBio HiFi, and ONT. However, no assembler ranks first in all evaluation categories, suggesting that the choice of assembler is often a trade-off between certain advantages and disadvantages. Therefore, we have corroborated the conclusion of Wick and Holt [7], who benchmarked long-read assemblers on prokaryotes, for eukaryotic organisms, and recommend that these benchmarking parameters are considered in relation to the desired outcome of an assembly experiment.

Additionally, the tests performed on real reads validate our rankings of simulated read assemblies. Flye, the assembler that scored consistently well in most evaluation categories for assemblies of simulated reads in PacBio CLR and ONT datasets, also ranks first when evaluated on several sets of real reads sequenced on long-read platforms.

In our analysis, we found that when processing HiFi reads, both LJA and Hifiasm assemblers showed better performance than other options. While LJA and Hifiasm may not always have been the absolute best, their high performance was a constant, irrespective of the dataset. This was not dataset specific but was consistently observed in both simulated and real datasets. This underscores their efficiency and accuracy in assembling genomic sequences using HiFi reads.

Regarding our second objective, which is addressing the effect of increased read length on assembly quality, the benchmarking of assemblers on read sets with different read length distributions suggests that longer reads have the potential to improve assembly quality. However, this depends on the size and complexity of the genome that is being reconstructed. We found that improvements in contiguity were most significant among all metrics, as also supported by the conclusion of [8], who showed that using third-generation sequencing considerably improves contiguity in assembling a plant genome (*M. jansenii*). However, we did not find significant improvements in other aspects of assembly quality, such as sequence identity or gene identification.

This study focused on comparison of different sequencing technologies and assemblers on a specific coverage level of 30×, which provided insights into the performance of different assemblers. However, it is important to recognize that assemblers may behave differently at lower or higher coverage levels, and project planners need guidance in selecting the right coverage for their goals and budget. Unfortunately, studying the effect of different coverages on assembly performance is not part of this study.

The field of genomics is continuously evolving, and advancements in sequencing technologies can significantly influence assembly outcomes. While our study focuses on benchmarking long-read *de novo* assembly tools for eukaryotic genomes, the rapid progress in sequencing technologies introduces complexities and challenges in comparing different data types, chemistries, and versions of the tools. In an ideal situation, it would be important to consider all the various factors, including different chemistries, sequencing devices, and base callers when evaluating assemblies. However, due to the limitations of available data and resources, we focused primarily on analyzing the impact of specific chemistry and related factors in this study. We recognize that this represents one of the limitations of our research.

The generations of HiFi reads have witnessed substantial advancements in both read length and accuracy. In earlier versions, HiFi reads typically had read lengths ranging from around 10 to 15 kilobases (kb) with high accuracy rates of 99.9% or greater. However, with subsequent generations, there has been a significant increase in read lengths. The latest versions of HiFi reads now offer read lengths exceeding 20 kb, with some reaching up to 30 kb or more, while still maintaining high accuracy rates above 99.9%. These longer and highly accurate HiFi reads provide researchers with more contiguous and reliable genomic sequences, enabling improved *de novo* assembly and enhancing various genomic analyses. An interesting innovation worth mentioning, while not included in this study, is the introduction of Oxford Nanopore’s Duplex reads. This cutting-edge technology holds the potential to enhance sequencing accuracy even further, making it a worthwhile subject for future investigations.

## Supplementary Material

giad100_GIGA-D-23-00008_Original_Submission

giad100_GIGA-D-23-00008_Revision_1

giad100_Response_to_Reviewer_Comments_Original_Submission

giad100_Reviewer_1_Report_Original_Submission Brandon Pickett -- 2/23/2023 Reviewed

giad100_Reviewer_2_Report_Original_Submission Luca Ermini -- 2/28/2023 Reviewed

giad100_Reviewer_2_Report_Revision_1 Luca Ermini -- 6/22/2023 Reviewed

giad100_Supplemental_File

## Data Availability

All additional supporting data are available in the *GigaScience* repository, GigaDB [35].

## Additional Files

**Supplementary Fig. S1**. Read length distribution of real *A. thaliana* ONT reads.

**Supplementary Fig. S2**. Read length distribution of real *A. thaliana* PacBio HiFi reads.

**Supplementary Fig. S3**. Read length distribution of real *C. elegans* ONT reads.

**Supplementary Fig. S4**. Read length distribution of real *C. elegans* PacBio CLR reads.

**Supplementary Fig. S5**. Read length distribution of real *D. ananassae* PacBio CLR reads.

**Supplementary Fig. S6**. Read length distribution of real *D. melanogaster* ONT reads.

**Supplementary Fig. S7**. Read length distribution of real *D. melanogaster* PacBio CLR reads.

**Supplementary Fig. S8**. Read length distribution of real *P. falciparum* ONT reads.

**Supplementary Fig. S9**. Read length distribution of real *P. falciparum* PacBio HiFi reads.

**Supplementary Fig. S10**. Read length distribution of real *S. cerevisiae* ONT reads.

**Supplementary Fig. S11**. Read length distribution of real *S. cerevisiae* PacBio HiFi reads.

**Supplementary Fig. S12**. Read length distribution of real *T. rubripes* PacBio CLR reads.

**Supplementary Fig. S13**. Read length distribution of real human PacBio CLR reads.

**Supplementary Fig. S14**. Read length distribution of real human ONT reads.

**Supplementary Table S1**. Assembly accession numbers for all 7 reference genomes used in the experiments.

**Supplementary Table S2**. Long read sets from the human genome used to generate Badread error and QScore models for PacBio CLR and ONT. Where needed, we downsampled reads to 3 gigabase pairs (Gbp), which meets the simulator’s requirements for at least 1 Gbp of real sequence data. Read identities were calculated as described by Wick [15], who used the definition of BLAST identity. The sequence data were aligned to reference GCF_009914755.1 [16], with Minimap v2.24 [17].

**Supplementary Table S3**. Badread parameters used in the simulation of PacBio CLR and ONT reads. In total, using Badread, we simulated 48 read sets, accounting for 6 genomes (Supplementary Table S1), 2 sequencing technologies, and 4 read length distributions per technology (Table 1). Aside from read length, these parameters were kept consistent for each technology across all simulations. All other parameters not included in this table were kept as the simulator’s defaults. Please note that the pacbio_human2019 and ont_human2019 models were generated in this study (see Supplementary Table S2).

**Supplementary Table S4**. Commands and parameters used for simulating PacBio HiFi reads using PBSIM3.

**Supplementary Table S5**. Accession for the sequencing data used in our benchmark of real read assemblies. To match our simulated reads, we have further downsampled these read sets to 30× coverage. Due to the low coverage of the *T. rubripes* PacBio and D. *melanogaster* ISO-1 Oxford Nanopore datasets, we combined multiple datasets and sampled reads to a coverage of approximately 30×.

**Supplementary Table S6**. Assembly commands for all assemblers. The $genome_size in the assembly commands below was set as follows: *S. cerevisiae* = 12 Mbp, *P. falciparum* = 23 Mbp, *A. thaliana* = 130 Mbp, *D. melanogaster* = 139 Mbp, *C. elegans* = 103 Mbp, *T. rubripes* = 384 Mbp, and *D. ananassae* = 217 Mbp. The $threads parameter was set to 8 for *S. cerevisiae* and *P. falciparum*; 16 for *A. thaliana, C. elegans, D. melanogaster*, and *D. ananassae*; and 20 for *T. rubripes*.

**Supplementary Table S7**. Definitions and formulas for the COMPASS metrics defined in Assemblathon 2 [13]. We define $C,\ V,\ M,\ P$ as the coverage, validity, multiplicity, and parsimony of an assembly, respectively. We also denote ${L}_{CI}$ as the total length of the coverage islands, ${L}_A$ as the total length of the alignments between the reference and the assembly, ${L}_R$ as the total length of the reference, and ${L}_S$ as the total length of the assembly (sum of the scaffold lengths).

**Supplementary Table S8**. Evaluation results for the *S. cerevisiae* Oxford Nanopore simulated read assemblies.

**Supplementary Table S9**. Evaluation results for the *S. cerevisiae* PacBio CLR simulated read assemblies.

**Supplementary Table S10**. Evaluation results for the *S. cerevisiae* PacBio HiFi simulated read assemblies.

**Supplementary Table S11**. Evaluation results for the *P. falciparum* Oxford Nanopore simulated read assemblies.

**Supplementary Table S12**. Evaluation results for the *P. falciparum* PacBio CLR simulated read assemblies.

**Supplementary Table S13**. Evaluation results for the *P. falciparum* PacBio HiFi simulated read assemblies.

**Supplementary Table S14**. Evaluation results for the *C. elegans* Oxford Nanopore simulated read assemblies.

**Supplementary Table S15**. Evaluation results for the *C. elegans* PacBio CLR simulated read assemblies.

**Supplementary Table S16**. Evaluation results for the *A. thaliana* Oxford Nanopore simulated read assemblies.

**Supplementary Table S17**. Evaluation results for the *A. thaliana* PacBio CLR simulated read assemblies.

**Supplementary Table S18**. Evaluation results for the *A. thaliana* PacBio Hifi simulated read assemblies.

**Supplementary Table S19**. Evaluation results for the *D. melanogaster* Oxford Nanopore simulated read assemblies.

**Supplementary Table S20**: Evaluation results for the *D. melanogaster* PacBio CLR simulated read assemblies.

**Supplementary Table S21**. Evaluation results for the *D. ananassae* PacBio HiFi simulated read assemblies.

**Supplementary Table S22**. Evaluation results for the *T. rubripe*s Oxford Nanopore simulated read assemblies.

**Supplementary Table S23**. Evaluation results for the *T. rubripes* PacBio CLR simulated read assemblies.

**Supplementary Table S24**. Evaluation results for the PacBio HiFi real read assemblies.

**Supplementary Table S25**. Runtime (minutes) and memory usage (GB) for all *S. cerevisiae* ONT and PacBio CLR simulated read assemblies.

**Supplementary Table S26**. Runtime (minutes) and memory usage (GB) for all *P. falciparum* ONT and PacBio CLR simulated read assemblies.

**Supplementary Table S27**. Runtime (minutes) and memory usage (GB) for all *C. elegans* ONT and PacBio CLR simulated read assemblies.

**Supplementary Table S28**. Runtime (minutes) and memory usage (GB) for all *A. thaliana* ONT and PacBio CLR simulated read assemblies.

**Supplementary Table S29**. Runtime (minutes) and memory usage (GB) for all *D. melanogaster* ONT and PacBio CLR simulated read assemblies.

**Supplementary Table S30**. Runtime (minutes) and memory usage (GB) for all *T. rubripes* ONT and PacBio CLR simulated read assemblies.

**Supplementary Table S31**. Evaluation results for all real read assemblies.

## Availability of Supporting Source Code and Requirements

Our evaluations were produced with QUAST v5.0.2 [31], BUSCO v5.4.2 [33, 34], and COMPASS [13]. We also provide the scripts we used in GitHub:

Project name: Long-read assembly benchmark

Project homepage: https://github.com/AbeelLab/long-read-assembly-benchmark

Operating system(s): Platform independent

Programming language: Python and Shell

License: GNU General Public License v3.0

## Abbreviations

BLAST: Basic Local Alignment Search Tool; BUSCO: Benchmarking Universal Single-Copy Orthologs; CLR: continuous long read; Gbp: gigabase pair; HiFi: high-fidelity; iTOL: Interactive Tree of Life; kb: kilobases; kbp: kilobase pair; Mbp: megabase pair; NGS: next-generation sequencing; ONT: Oxford Nanopore; PacBio: Pacific Biosciences; TGS: third-generation sequencing.
