# Supplementary material for: Evaluating long-read de novo assembly tools for eukaryotic genomes: insights and considerations
Source: Gigascience. 2023 Nov 24;12:giad100. doi: 10.1093/gigascience/giad100 (PMC10673639; doi:10.1093/gigascience/giad100)
Supplement: giad100_GIGA-D-23-00008_Revision_1 [file giad100_giga-d-23-00008_revision_1.pdf]

GigaScience

Evaluating Long Read De Novo Assembly Tools for Eukaryotic Genomes: Insights and Considerations

--Manuscript Draft--

**Manuscript Number:**	GIGA-D-23-00008R1
**Full Title:**	Evaluating Long Read De Novo Assembly Tools for Eukaryotic Genomes: Insights and Considerations
**Article Type:**	Research
**Funding Information:**	
**Abstract:**	Background: Assembly algorithm choice should be a deliberate, well-justified decision when researchers create genome assemblies for eukaryotic organisms from third-generation sequencing technologies. While third-generation sequencing by Oxford Nanopore Technologies (ONT) and Pacific Biosciences (PacBio) have overcome the disadvantages of short read lengths specific to next-generation sequencing (NGS), third-generation sequencers are known to produce more error-prone reads, thereby generating a new set of challenges for assembly algorithms and pipelines. However, the introduction of HiFi reads, which offer substantially reduced error rates, has provided a promising solution for more accurate assembly outcomes. Since the introduction of third-generation sequencing technologies, many tools have been developed that aim to take advantage of the longer reads, and researchers need to choose the correct assembler for their projects.<br>Results: We benchmarked state-of-the-art long-read de novo assemblers, to help readers make a balanced choice for the assembly of eukaryotes. To this end, we used 12 real and 64 simulated datasets from different eukaryotic genomes, with different read length distributions, imitating PacBio CLR, PacBio HiFi, and ONT sequencing to evaluate the assemblers. We include five commonly used long read assemblers in our benchmark: Canu, Flye, Miniasm, Raven, and wtdbg2 for ONT and PacBio CLR reads. For PacBio HiFi reads LJA, we include five state-of-the-art HiFi assemblers: HiCanu, Flye, Hifiasm, LJA and MBG. Evaluation categories address the following metrics: reference-based metrics, assembly statistics, misassembly count, BUSCO completeness, runtime, and RAM usage. Additionally, we investigated the effect of increased read length on the quality of the assemblies, and report that read length can, but does not always, positively impact assembly quality.<br>Conclusions: Our benchmark concludes that there is no assembler that performs the best in all the evaluation categories. However, our results show that overall Flye is the best-performing assembler for PacBio CLR and ONT reads, both on real and simulated data. Meanwhile, best performing PacBio HiFi assemblers are Hifiasm, and LJA. Next, the benchmarking using longer reads shows that the increased read length improves assembly quality, but the extent to which that can be achieved depends on the size and complexity of the reference genome.
**Corresponding Author:**	Thomas Abeel<br>TU Delft: Technische Universiteit Delft<br>NETHERLANDS
**Corresponding Author Secondary Information:**	
**Corresponding Author's Institution:**	TU Delft: Technische Universiteit Delft
**Corresponding Author's Secondary Institution:**	
**First Author:**	Bianca-Maria Cosma
**First Author Secondary Information:**	
**Order of Authors:**	Bianca-Maria Cosma
	Ramin Shiarli Hossein Zade
	Erin Noel Jordan

Powered by Editorial Manager® and ProduXion Manager® from Aries Systems Corporation

	Paul van Lent
	Chengyao Peng
	Stephanie Pillay
	Thomas Abeel
**Order of Authors Secondary Information:**	
**Response to Reviewers:**	Dear Editor,<br><br>In order to be able to use different colors in our responses, we have uploaded a document named Response_letter.docx which includes the reviewers comments and our responses.
**Additional Information:**	
**Question**	**Response**
Are you submitting this manuscript to a special series or article collection?	No
**Experimental design and statistics**<br><br>Full details of the experimental design and statistical methods used should be given in the Methods section, as detailed in our Minimum Standards Reporting Checklist. Information essential to interpreting the data presented should be made available in the figure legends.<br><br>Have you included all the information requested in your manuscript?	Yes
**Resources**<br><br>A description of all resources used, including antibodies, cell lines, animals and software tools, with enough information to allow them to be uniquely identified, should be included in the Methods section. Authors are strongly encouraged to cite Research Resource Identifiers (RRIDs) for antibodies, model organisms and tools, where possible.<br><br>Have you included the information requested as detailed in our Minimum Standards Reporting Checklist?	Yes
**Availability of data and materials**	Yes

Powered by Editorial Manager® and ProduXion Manager® from Aries Systems Corporation

All datasets and code on which the conclusions of the paper rely must be either included in your submission or deposited in publicly available repositories (where available and ethically appropriate), referencing such data using a unique identifier in the references and in the "Availability of Data and Materials" section of your manuscript.<br><br>Have you have met the above requirement as detailed in our Minimum Standards Reporting Checklist?	

Powered by Editorial Manager® and ProduXion Manager® from Aries Systems Corporation

Manuscript

Click here to access/download;Manuscript;Manuscript-Oct 25_final.docx

Evaluating Long Read De Novo Assembly Tools for Eukaryotic Genomes: Insights and Considerations

Bianca-Maria Cosma[1], Ramin Shirali Hossein Zade[1], Erin Noel Jordan[1,2], Paul van Lent[1], Chengyao Peng[1], Stephanie Pillay[1], and Thomas Abeel[1,3,*]

[1]Delft Bioinformatics Lab, Delft University of Technology Van Mourik, Broekmanweg 6, 2628 XE, Delft, The Netherlands; [23]Technical Biochemistry, TU Dortmund University, Emil-Figge-Straße 66, 44227, Dortmund, Germany; and [4]Infectious Disease and Microbiome Program, Broad Institute of MIT and Harvard, 415 Main Street, Cambridge, MA, 02142, USA

*t.abeel@tudelft.nl

Bianca-Maria Cosma [0009-0000-1447-2701];

Hossein Zade [0000-0003-2624-0718];

Erin Noel Jordan [0000-0003-2022-0679];

Paul van Lent [0009-0001-2887-0193];

Chengyao Peng [0000-0002-9765-4400];

Stephanie Pillay [0000-0002-4219-8698];

Thomas Abeel [0000-0002-7205-7431].

Abstract

Background: Assembly algorithm choice should be a deliberate, well-justified decision when researchers create genome assemblies for eukaryotic organisms from third-generation sequencing technologies. While third-generation sequencing by Oxford Nanopore Technologies (ONT) and Pacific Biosciences (PacBio) have overcome the disadvantages of short read lengths specific to next-generation sequencing (NGS), third-generation sequencers are known to produce more error-prone reads, thereby generating a new set of challenges for assembly algorithms and pipelines. However, the introduction of HiFi reads, which offer substantially reduced error rates, has provided a promising solution for more accurate assembly outcomes. Since the introduction of third-generation sequencing technologies, many tools have been developed that aim to take advantage of the longer reads, and researchers need to choose the correct assembler for their projects.

Results: We benchmarked state-of-the-art long-read *de novo* assemblers, to help readers make a balanced choice for the assembly of eukaryotes. To this end, we used 12 real and 64 simulated datasets from different eukaryotic genomes, with different read length distributions, imitating PacBio CLR, PacBio HiFi, and ONT sequencing to evaluate the assemblers. We include five commonly used long read assemblers in our benchmark: Canu, Flye, Miniasm, Raven, and wtdbg2 for ONT and PacBio CLR reads. For PacBio HiFi reads LJA, we include five state-of-the-art HiFi assemblers: HiCanu, Flye, Hifiasm, LJA and MBG. Evaluation categories address the following metrics: reference-based metrics, assembly statistics, misassembly count, BUSCO completeness, runtime, and RAM usage. Additionally, we investigated the effect of increased read length on the quality of the assemblies, and report that read length can, but does not always, positively impact assembly quality.

Conclusions: Our benchmark concludes that there is no assembler that performs the best in all the evaluation categories. However, our results show that overall Flye is the best-performing assembler for PacBio CLR and ONT reads, both on real and simulated data. Meanwhile, best performing PacBio HiFi assemblers are Hifiasm, and LJA. Next, the benchmarking using longer reads shows that the increased read length improves assembly quality, but the extent to which that can be achieved depends on the size and complexity of the reference genome.

Key words: *De novo* assembly, Third-generation sequencing, Benchmarking, Eukaryote genomes.

Introduction

De novo genome assembly is essential in several leading fields of research, including disease identification, gene identification, and evolutionary biology [1–4]. Unlike reference-based assembly, which relies on the use of a reference genome, *de novo* assembly only uses the genomic information contained within the sequenced reads. Since it is not constrained to the use of a reference, high quality *de novo* assembly is essential for studying novel organisms, as well as for the discovery of overlooked genomic features, such as gene duplication [5], in previously assembled genomes.

The introduction of Third Generation Sequencing (TGS) led to massive improvements in *de novo* assembly. The advent of TGS has addressed the main drawback of Next Generation Sequencing (NGS) platforms, namely the short read length, but has introduced new challenges in genome assembly, because of the higher error rates of long reads. The leading platforms in long-read sequencing are Pacific Biosciences Single Molecule, Real-Time sequencing (often abbreviated as "PacBio") and Oxford Nanopore (ONT) sequencing [6].

Since the introduction of TGS platforms, many methods have been developed that aim to take the most benefits from the longer read length and overcome the new challenges due to sequencing error. Recent studies have been conducted to compare long-read *de novo* assemblers. One such study was conducted by Wick and Holt [7], who focused on long-read *de novo* assembly of prokaryotic genomes. Eight assemblers were tested on real and simulated reads from PacBio and ONT sequencing, and evaluation metrics included sequence identities, circularisation of contigs, computational resources, as well as accuracy. Murigneux et al. [8] performed similar experiments on the genome of *M. jansenii*, although in this case, the focus was on comparatively benchmarking Illumina sequencing and three long-read sequencing technologies, in addition to the comparison of long-read assembly tools. Studies narrowed down to just one type of sequencing technology include those of Jung et al. [9], who evaluated assemblers on real PacBio reads from five plant genomes, and Chen et al. [10], who used Oxford Nanopore real and simulated reads from bacterial pathogens in their comparison. Except for the Wick and Holt study, which provides a compressive comparison on *de novo* assembly of prokaryotic genomes, other studies are either comparing the assemblers on single genome or using data from a single sequencing platform. Here, we provide a comprehensive comparison on *de novo* assembly tools on the most used TGS technologies and 7 different eukaryotic genomes, to complement the study of Wick and Holt.

In this study, we are benchmarking these methods using 12 real and 64 simulated datasets (see Figure 1) from PacBio CLR, PacBio HiFi and ONT platforms to guide researchers to choose the proper

assembler for their studies. Benchmarking using simulated reads allows us to accurately compare the final assembly with the ground truth, and benchmarking using the real reads can validate the results based on simulated reads. The assembler comparison presented in this manuscript complements the literature that has already been published, by introducing an analysis of not just assembler performance, but also of the effect of read length on assembly quality. Although increased read length is considered an advantage, we investigate if it is always a necessary advantage to have for assembly performance. To that end, the scope of the study extends to six model eukaryotes that provide a performance indication for genomes of variable complexity, covering a wide range of taxa on the eukaryotic branch of the Tree of Life [11]. Complexity in genome assembly is determined by multiple variables, the most notable of which is the proportion of repetitive sequences within the genome of a particular organism. Complexity in eukaryotic genomes is further exacerbated by size and organization of chromosomal architecture, including telomeres and centromeres, and the presence of circular elements such as mitochondrial and chloroplast DNA.

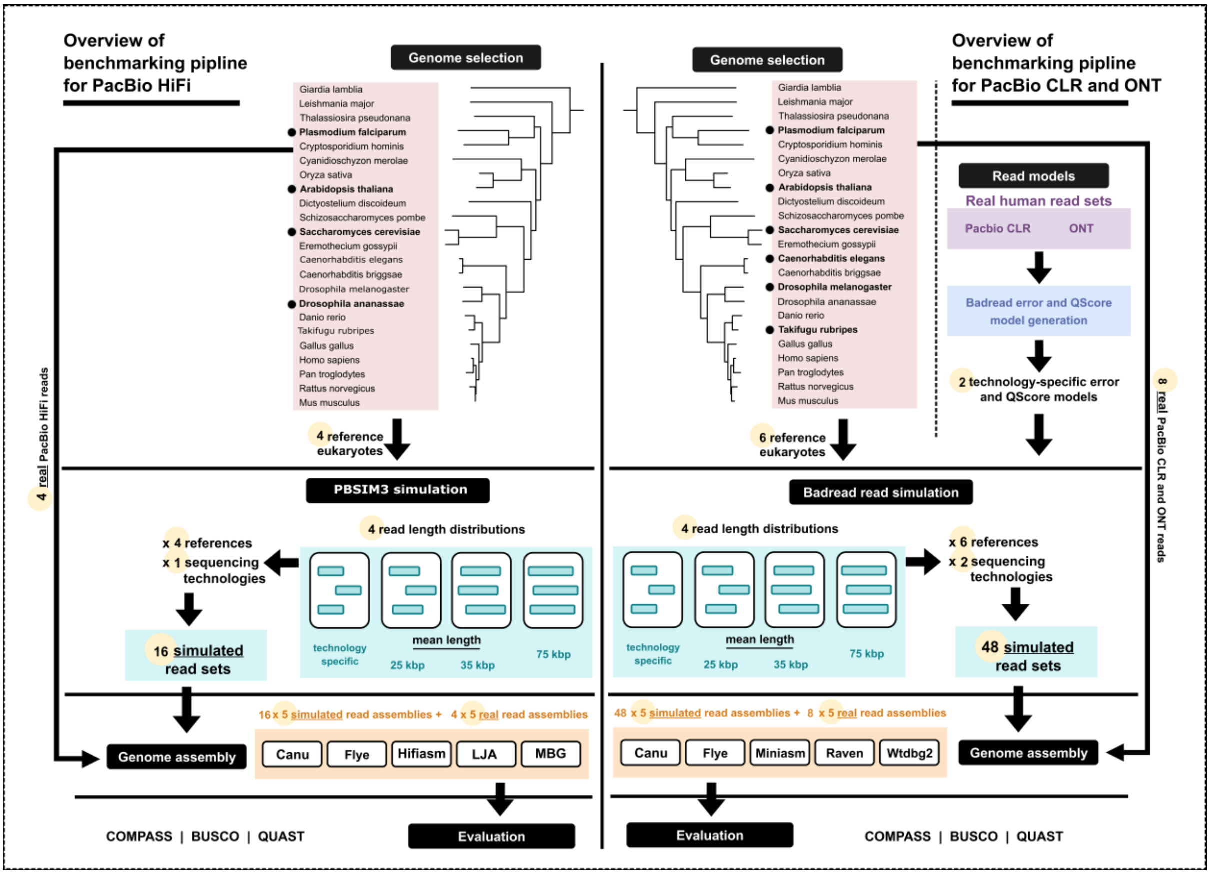

Figure 1: The benchmarking pipeline. For PacBio CLR and ONT (right panel), first we select 6 representative eukaryotes from the Tree of Life (Letunic and Bork, 2021) and use Badread's error and Qscore model generation feature (Wick, 2019) to create 2 models of PacBio CLR and ONT long sequencing technologies. This is input to the read simulation stage, where we simulate reads from all genomes, with four different read length distributions. We then perform assembly of simulated and real reads, using five long-read assemblers. For PacBio HiFi (left panel), first we select 4 representative eukaryotes and use PBSIM3 to simulate HiFi reads. These reads are then assembled using five state-of-the-art HiFi assemblers. Lastly, we evaluate all PacBio HiFi, PacBio CLR and ONT assemblies based on several criteria

De novo genome assembly evaluation remains challenging, as it represents a process that must account for variables such as the goal of an assembly and the existence of a ground-truth reference. A standard evaluation procedure was introduced in the literature by the two Assemblathon competitions [12,13], which outlined a selection of metrics that encompasses the most relevant aspects of genome assembly, however, these metrics require a reference sequence. Most of these metrics are adopted in our benchmark.

Consequently, this study addresses two main objectives. First, we provide a systematic comparison of state-of-the-art long-read assembly tools, documenting their performance in assembling real and simulated PacBio Continuous Long (CLRs), PacBio High fidelity (HiFi), and Oxford Nanopore (ONT) reads on a diverse set of eukaryotic organisms. The PacBio CLR and ONT reads generated from the genomes of *S. cerevisiae*, *P. falciparum*, *C. elegans*, *A. thaliana*, *D. melanogaster*, and *T. rubripes* and the PacBio HiFi reads are generated from the genomes of *S. cerevisiae, P. falciparum, A. thaliana and D. ananassae.* Our second objective is to investigate whether increased read length has a positive effect on overall assembly quality, given that increasing the length of reads is an on-going effort in the development of Third Generation Sequencing platforms [14].

It is important to note that our objective is to evaluate the performance of these tools in generating a consensus assembly without taking haplotypes into account. Moreover, it is crucial to highlight that the results and conclusions drawn from this comparison may not be directly applicable to metagenome assembly. The unique characteristics and complexities associated with metagenomic data warrant a separate and distinct analysis, which is beyond the scope of this study.

Materials and methods

Data

In this study, we are using real and simulated data from various organisms to benchmark long read *de novo* assembly tools.

Reference genomes

We selected six reference genomes from eukaryotic organisms represented in the Interactive Tree Of Life (iTOL) v6 [11] for evaluating PacBio CLR and ONT assemblers: *S. cerevisiae* (strain S288C), *P. falciparum* (isolate 3D7), *C. elegans* (strain VC2010), *A. thaliana* (ecotype Col-0), *D. melanogaster* (strain ISO-1), and *T. rubripes*. Moreover, we selected the four eukaryotic organisms to evaluate PacBio HiFi assemblers: *S. cerevisiae* (strain S288C), *P. falciparum* (isolate 3D7), *A. thaliana* (ecotype

Col-0), and *D. ananassae* (strain 14024-0371.13). Assembly accessions are included in Supplementary Table S1.

The reference assemblies for *C. elegans*, *D. melanogaster*, and *T. rubripes* included uncalled bases. In these cases, before read simulation, each base N was replaced with base A, as done by Wick and Holt [7]. This avoids ambiguity in the read simulation process and consequently simplifies the evaluation of the simulated-read assemblies. As such, we used this modified version as a reference when evaluating all assemblies of simulated reads from these four genomes. In the evaluation of real-read assemblies, the original assemblies were used as references.

Simulated reads

The PacBio CLR and ONT simulated read sets were generated using Badread v0.2.0 [15]. To create read error and Qscore (quality score) models in addition to the simulator's own default models, Badread requires the following three parameters: a set of real reads, a high-quality reference genome, and an alignment file, obtained by aligning the reads to the reference genome. We used real read sets from the human genome to create error and Qscore models that reflect the state-of-the-art for PacBio Continuous Long Reads (CLRs), and Oxford Nanopore reads. The simulated PacBio HiFi reads were generated using PBSIM3. To generate reads similar to HiFi, we used –num-pass 10 parameter, and then applied ccs version 6.4.0 to generate the consensus reads.

To create the models for PacBio CLR and Oxford Nanopore reads, we used the real read sets sequenced from the human genome and aligned to the latest high-quality human genome reference assembled by [16]: assembly T2T-CHM13v2.0, with RefSeq accession GCF_009914755.1. The alignment was performed using Minimap2 v2.24 (RRID:SCR_018550) [17] with default parameters. The sources for these sequencing data are outlined in Supplementary Table S2, as well as the read identities for each technology, which are later passed as parameters for the simulation stage.

To study the effect of read length on genome assembly, we simulated reads that imitate PacBio CLR, PacBio HiFi, and Oxford Nanopore sequencing, with four different read length distributions, using Badread for PacBio CLR and Oxford Nanopore sequencing while using PBSIM3 for PacBio HiFi. The first read simulation represents the current state of the three long-read technologies. The other three simulations reflect data points in-between technology-specific values and ultra-long reads, data points of a similar length as ultra-long-reads, and longer than ultra-long reads. We need to define the mean and standard deviation of the read length distributions for these simulations. The values for the mean and standard deviation of these distributions were selected as follows. First, we calculated the read length distributions of the real read sets in Supplementary Table S2 and simulated an initial iteration of reads using these technology-specific values. For choosing these values for the other three iterations, we analysed a set of Oxford Nanopore Ultra-Long reads used in the latest assembly of the human genome [16]. We selected GridION run SRR12564452, available as sequence data in BioProject PRJNA559484, with a mean read length of approximately 35.7 kbp, and a standard deviation of 42.5 kbp. A summary of the Badread and PBSIM3 commands used in our simulation can be found in Supplementary Table S3 and S4.

A full overview of the mean and standard deviation of all four read length distributions is given in Table 1. Note that, for each of the technologies, the standard deviation for the last three distributions was derived from the mean, using the ratio between the mean and standard deviation reflected by the technology-specific values. Hence, for the last three iterations, the mean read length is consistent across sequencing technologies, but the standard deviation varies.

Table 1: The mean and standard deviation describing the read length distributions used in our simulations. Note that read length increases with each iteration, and the distribution parameters are different for each technology.

	**Read length distribution parameters (kbp), per technology**					
	**PacBio CLR**		**PacBio HiFi**		**Oxford Nanopore**	
	**Mean**	**Stdev**	**Mean**	**Stdev**	**Mean**	**Stdev**

**Iteration 1** (technology-specific values)	15.7	14.4	20.7	2.5	12.1	17.1
**Iteration 2**	25	22.5	25	3	25	35
**Iteration 3** (imitate ultra-long reads)	35	31.5	35	4.2	35	49
**Iteration 4**	75	67.5	75	9	75	105

Consequently, we ran the simulations for each reference genome. As described above, we used our own models for each technology, and passed them to the simulator as the `–error_model` and `–qscore_model`. The read identities per technology were set to the values included in Supplementary Table S3. Across all simulations, we chose a coverage depth of 30x. Canu's documentation [18] specifies a minimum coverage of 20 – 25x for HiFi data, and 20x for other types of data, while Flye's guidelines [19] indicate a minimum coverage of 30x. As there is no minimum recommended coverage indicated for the other assemblers we used in our benchmark, we simulated reads following the stricter guideline among these two, that is, 30x coverage.

Real reads

In support of our evaluation on simulated reads, we also performed a benchmark on real-read assemblies from Oxford Nanopore and PacBio reads sequenced from the reference genomes. These reads were sampled to approximately 30x coverage, to avoid introducing potentially confounding variables when comparing assemblies of real and simulated datasets. The data sources for all real sets are included in Supplementary Table S5. Please note that the PacBio CLR data from *C. elegans* was generated using the older RSII technology. These reads inherent characteristics of the RSII system, such as shorter average reads and a higher error rate, which might have influenced the assembly results.

Assemblies

For the PacBio CLR and ONT reads, we included the following five long-read *de novo* assemblers : Canu v2.2 (RRID:SCR_015880) [18], Flye v2.9 (RRID:SCR_017016) [19], Wtdbg2 (also known as Redbean)

v2.5 (RRID:SCR_017225) [20], Raven v1.7.0 (RRID:SCR_001937) [21], and Miniasm v0.3_r179 (RRID:SCR_024114) [22]. For the PacBio HiFi reads, we included HiCanu v2.2 [23], Flye v2.9, Hifiasm 0.19.5-r587 [24], LJA v 0.2 [25], and MBG v 1.0.14 [26]. We used the most recent releases of the assemblers at the time we started this study.

The assemblies were performed with default values for most parameters. Canu and Wtdbg2 require the estimated genome size as a parameter, and we set the following values: *S. cerevisiae* = 12 Mbp, *P. falciparum* = 23 Mbp, *A. thaliana* = 135 Mbp, *D. melanogaster* = 139 Mbp, *C. elegans* = 103 Mbp, and *T. rubripes* = 384 Mbp, *D. ananassae* = 217 Mbp. All commands used in the assembly pipelines are available in Supplementary Table S6. We note that further polishing of assemblies using high-fidelity short reads, although common in practice [27–29], is omitted in this study, as the focus is exclusively on assembler performance on long-read data and not polishing tools.

We added a long-read polishing step for Miniasm and Wtdbg2, as their assembly pipelines do not include long-read based polishing. Following Raven's default pipeline, which performs two rounds of Racon polishing [30], we used two rounds of Racon polishing on Wtdbg2 and Miniasm. We note that for Miniasm, we used Minipolish [7], which simplifies Racon polishing by applying it in two iterations on the GFA (Graphical Fragment Assembly) files produced by the assembler. For both Miniasm and Wtdbg2, the alignments required for polishing were generated with Minimap v2.24.

Evaluation

We evaluated the assemblies in three different categories of metrics. The COMPASS analysis compares the assemblies with their corresponding reference genome and provides insight into their similarities. The assembly statistics provide some basic knowledge about the contiguity and misassemblies. Finally, the BUSCO assessment investigates the presence of essential genes in the assemblies. These three categories of metrics, next to each other, can provide a complete overview of the assembly's quality.

Correctness analysis

For each assembly, we ran the COMPASS script to measure the coverage, validity, multiplicity, and parsimony, to assess the quality of the assemblies, as defined in Assemblathon 2 [13]. These metrics describe several characteristics that were deemed important for comparing *de novo* assembly tools, and they were computed using three types of data: (1) the reference sequence, (2) the assembled scaffolds, and (3) the alignments (sequences from the assembled scaffolds that were aligned to the reference sequences). Definitions and formulas for the metrics are reported in Supplementary Table S7.

Moreover, we use QUAST v5.2.0 (RRID:SCR_001228) [31] to calculate the number of misassemblies. QUAST identifies misassemblies based on the definition outlined by [32]. The total number of misassemblies is the sum of all relocations, inversions, and translocations. Considering two adjacent flanking sequences, if they both align to the same chromosome, but 1 kbp away from each other, or overlapping for more than 1 kbp, this is counted as a relocation. If these flanking sequences, aligned to the same chromosome, are on opposite strands, the misassembly is considered an inversion. Lastly, translocations describe events in which two flanking sequences align to different chromosomes.

Contiguity assessment

We use QUAST v5.2.0 [31] is used to measure the auNG of an assembly. The auNG metric, standing for the area under the NG50 [12] curve, is a measure of assembly contiguity. By evaluating the area beneath the NG50 curve as each sequence fragment or contig is included, it provides a more thorough understanding of the contiguity of the assembly compared to single-value metrics. A larger auNG value indicates better contiguity in the genome assembly.

Completeness assessment

BUSCO v5.4.2 (RRID:SCR_015008) assessment [33,34] is performed to evaluate the completeness of the essential genes in the assemblies. This quantifies the number of single-copy, duplicated, fragmented and missing orthologs in an assembled genome. From the number of orthologs specific to

each dataset, BUSCO identifies how many orthologs are present in the assembly (either as single-copy or duplicated), how many are fragmented, and how many are missing. We ran these evaluations with a different OrthoDB lineage dataset for each genome: *S. cerevisiae* – `saccharomycetes`, *P. falciparum* – `plasmodium`, *A. thaliana* – `brassicales`, *D. melanogaster* – `diptera`, *C. elegans* – `nematoda`, *T. rubripes* – `12ctinopterygii`, and *D. ananassae* - `diptera`.

Results and discussion

Overview of the benchmarking pipeline

Figure 1 shows an overview of the benchmarking pipeline. For the PacBio CLR and Oxford Nanopore reads we begin with the selection of six representative eukaryotes from the interactive Tree of Life [11]: *Saccharomyces cerevisiae*, *Plasmodium falciparum*, *Arabidopsis thaliana*, *Drosophila melanogaster* , *Caenorhabditis elegans*, and *Takifugu rubripes*. We also use three read sets from the latest human assembly project [16] to generate Badread error and Qscore models [15] for PacBio Continuous Long Reads (CLRs), and Oxford Nanopore reads (see Supplementary Table S2). The reference sequences and models become input to the Badread simulation stage. For each genome, we simulate reads with four different read length distributions and two sequencing technologies (see Table 1), amounting to a total of 8 simulated read sets per reference genome. These reads, as well as real read sets, are assembled with five assembly tools: Canu, Flye, Miniasm, Raven, and Wtdbg2.

For the PacBio HiFi reads we begin with the reference genome of the 4 selected eukaryote species: *Saccharomyces cerevisiae*, *Plasmodium falciparum*, *Arabidopsis thaliana,* and *Drosophila ananassae*. Then we use PBSIM3 and CCS to generate PacBio HiFi reads. Similar to the previous setup, for each reference genome we simulate reads with four different read length distributions. The simulated reads along with real reads for each of the four reference genomes are assembled with five assembly tools: HiCanu, Flye, Hifiasm, LJA, and MBG.

Next, the resulting assemblies are evaluated using COMPASS, QUAST, and BUSCO, and based on the reported metrics we distinguish six main evaluation categories: sequence identity, repeat collapse, rate of valid sequences, contiguity, misassembly count, and gene identification. The selected COMPASS metrics are the coverage, multiplicity, and validity of an assembly, which provide insight on sequence identity, repeat collapse, and the rate of valid sequences, respectively. In this regard, an ideal assembly has coverage, multiplicity and validity close to 1. This suggests that a large fraction of the reference genome is assembled, repeats are generally collapsed Instead of replicated, and most sequences in the assembly are validated by the reference. Among others, QUAST reports the number of misassemblies and the auNG of an assembly. A high auNG value indicates high contiguity. In order to assess contiguity across genomes of different sizes, we report the ratio between the assembly's auNG and the N50 of the references. Lastly, gene identification is quantified in terms of the percentage of complete BUSCOs in an assembly.

The search for an optimal assembler for PacBio CLR and ONT reads is influenced by read sequencing technology, genome complexity, and research goal

To select an assembler that is most versatile across eukaryotic taxa, we simulate PacBio Continuous Long Reads (CLRs), and Oxford Nanopore reads from the genomes of six eukaryotes, assemble these reads, and evaluate the assemblers in the six main categories mentioned in the previous section. The results for each evaluation category are normalized in the range given by the worst and best values encountered in the evaluation of all assemblies of reads with default length. This highlights differences between assemblers, as well as between genomes and sequencing technologies.

The results of the benchmark on the PacBio CLR and ONT read sets with default lengths, namely those belonging to the first iteration (see Table 1), are illustrated in Figure 2. A full report of the evaluation metrics in this figure is included in the Supplementary Tables S8 – S24, under "Iteration 1". We note that no assembler unanimously ranks first in all categories, across different sequencing technologies and eukaryotic genomes, although our findings highlight some of their strengths and thus their

potential for various research aims. The runtime and memory usage of the assembly tools on all of the simulated datasets are reported in Supplementary Tables S25 – S30, since this can also be a deciding factor next to the quality of the assembly for the researchers to choose the suitable assembler for their purpose. We note that all assemblies were run on our local High Performance Computing Cluster, and the runtime and RAM usage may have been affected by the heterogeneity of the shared computing environment in which the assembly jobs executed.

While working with PacBio CLR and ONT reads, Miniasm, Raven, and Wtdbg2 are all well-rounded choices for the simpler *S. cerevisiae*, *P. falciparum* and *C. elegans* genomes, with a balanced trade-off between assembly quality and computational resources. For PacBio HiFi reads, Raven is generally qualitatively outperformed by other assemblers like Canu, Flye, and Miniasm, likely as a consequence of the fact that its pipeline is not customized for all long-read sequencing technology. Nonetheless, if computational resources are a concern, Raven is a more suitable choice, since Miniasm and Wtdbg2 do not scale well for larger genomes.

We can single out Flye as the most robust assembler for PacBio CLR and ONT reads across all six organisms, although for larger genomes such as *T. rubripes*, Canu is a better tool. Both produce assemblies with high sequence identity and validity, as well as good gene prediction, but Flye assemblies generally rank first when we compute the average score across all six metrics. For Canu, we notice more variation in assembly quality across different genomes, particularly for *P. falciparum* and *A. thaliana*, while Flye maintains more consistent results. Nonetheless, on the *T. rubripes* genome, Canu assemblies have higher sequence identity and contiguity, as well as more accurate gene identification.

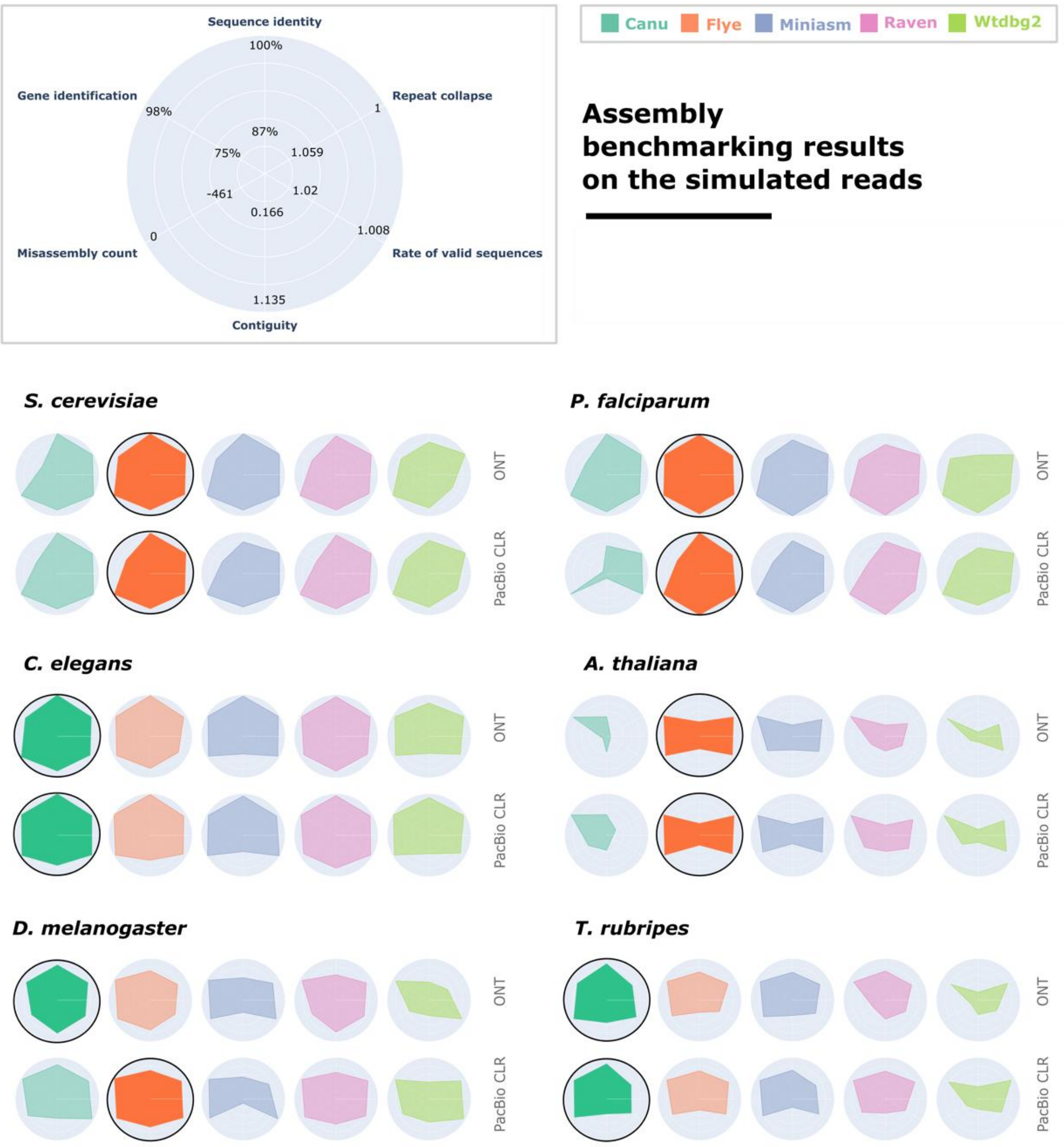

Figure 2: The performance of the five assemblers on the read sets with default read lengths, from iteration 1 (see Table 1), generated from six eukaryotic genomes. Six evaluation categories are reported for each assembler, and the results are normalized among all assemblies included in the figure. Ranges for each metric are reported as the best and worst values computed for these assemblies. The best performing assembler is highlighted and has a black outline.

Evaluation of PacBio CLR and ONT real-read assemblies supports our rankings on simulated-read assemblies

To determine assembler performance on real PacBio CLR and ONT reads and validate the rankings of the simulated-read assemblies, we assemble several real read sets from the six reference eukaryotes (Supplementary Table S5). The Supplementary Figures S1-S12 provides a visual representation of the read length distribution for all of the real read sets. The evaluation results on the real-read assemblies, summarized in Figure 3, indicate that assemblers which perform well on simulated reads perform similarly well in assembling the sets of real reads. The full report of metrics on the real read assemblies is included in Supplementary Table S31. We conclude that, overall, the assembler rankings remain consistent. This illustrates that benchmarking using simulated data is similar to real read sets. For reference-based metrics, we used the reference genomes given in Supplementary Table S1.

Notably, reference-based metrics in the evaluation of real-read assemblies rely on comparisons with an assembly, and not the genome from which the reads were initially sequenced. In contrast to the evaluation of simulated-read assemblies, the existence of a ground truth reference is not available in this case, but reference-based metrics are included for the sake of consistency with the simulated-read evaluation.

In the evaluation of real-read assemblies of PacBio CLR and ONT reads, Flye ranks first for nearly all datasets, with the exception of the *T. rubripes* and *C. elegans* PacBio reads, for which Raven performs better overall. However, even in *C. elegans*, Flye performance is close to the best values in all metrics other than contiguity. As expected, overall assembler performance decreases for reference-based metrics like sequence identity, repeat collapse and validity, but surprisingly the misassembly count is considerably lower.

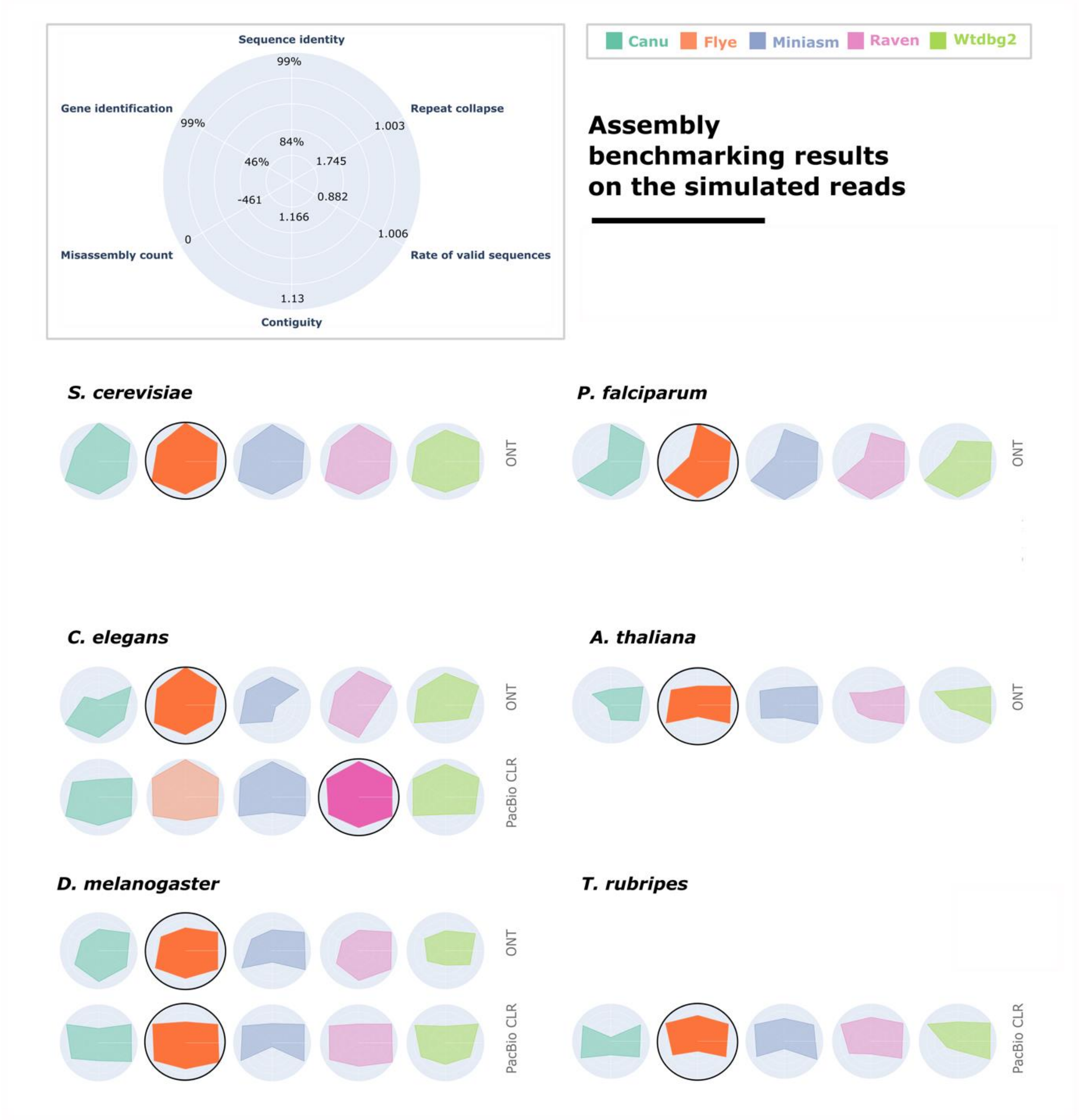

Figure 3: The performance of the five assemblers on the real PacBio CLR and ONT reads (see Supplementary Table S5), sequenced from six eukaryotic genomes. As in Figure 2, six evaluation categories are reported for each assembler, and the results are normalized among all assemblies included in the figure. Ranges for each metric are reported as the best and worst values computed for these assemblies. The best performing assembler is highlighted and has a black outline.

Searching for the best HiFi assembler based on simulated and real datasets

Similarly, in order to identify the best performing HiFi assembler for diverse eukaryotic taxa, we first generate simulated PacBio HiFi reads from the genomes of four different eukaryotes. These simulated reads are then assembled, and the performance of each assembler is evaluated based on the six

primary categories outlined in the previous section. For comparative clarity, the results for each evaluation category are normalized within the range established by the lowest and highest values observed across all assembly evaluations of reads of default length. This method emphasizes both the variations among different assemblers, as well as the discrepancies across genomes and sequencing technologies.

The results from simulated PacBio HiFi read sets with default lengths, namely those belonging to the first iteration (see Table 1), are illustrated in Figure 4. Next to that, the results of real HiFi reads of the same species are presented in Figure 4. We note that Hifiasm, and LJA are outperforming other assemblers and perform well in all datasets. The assembly results generated by the MBG assembler demonstrated notably low sequence identity when compared to the reference genome.

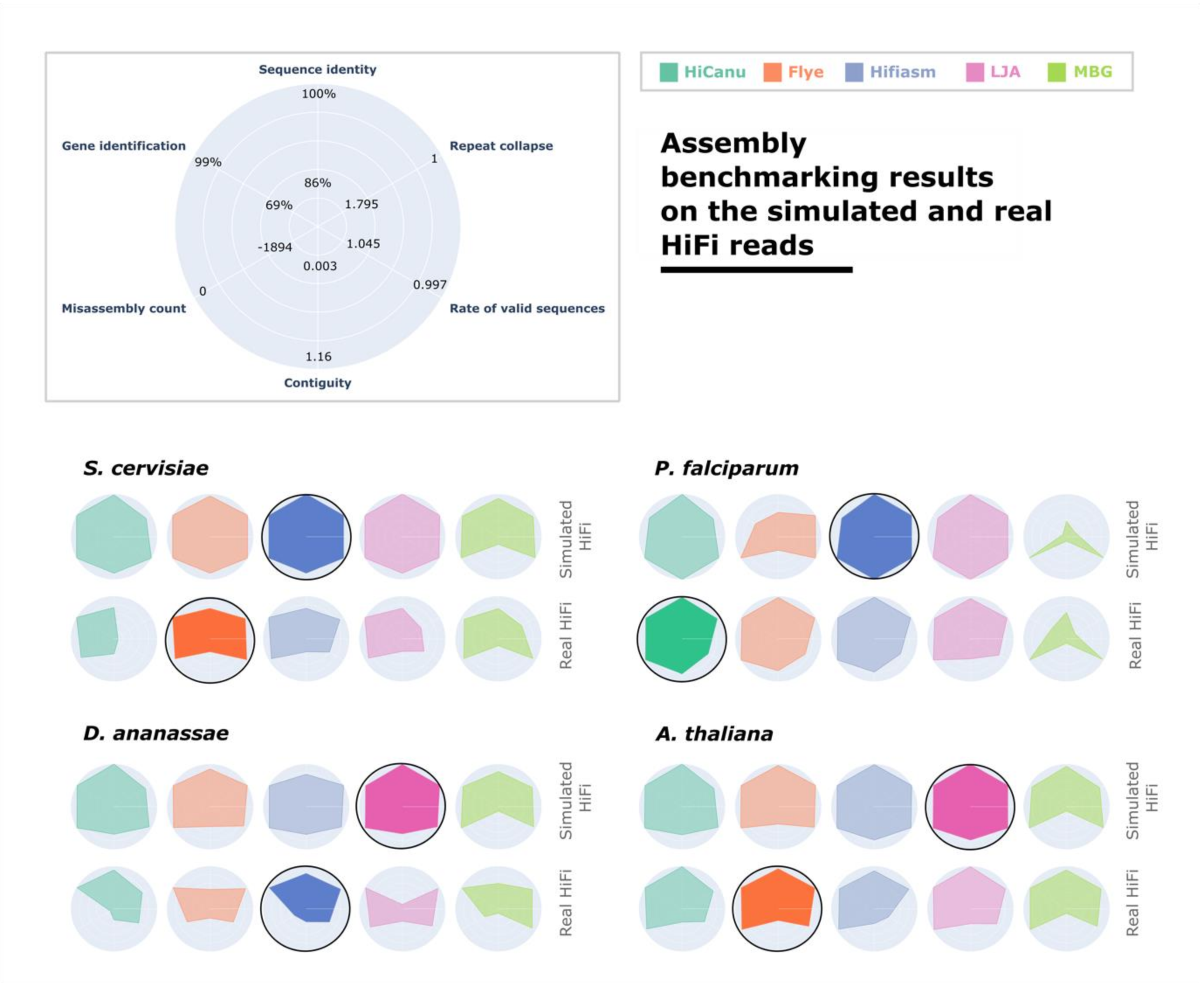

Figure 4: The performance of the five assemblers on the real PacBio HiFi read sets and simulated PacBio HiFi read sets with default read lengths, from iteration 1 (see Table 1), generated from four eukaryotic genomes. Six evaluation categories are reported for each assembler, and the results are normalized among all assemblies included in the figure. Ranges for each metric are reported as the best and worst values computed for these assemblies. The best performing assembler is highlighted and has a black outline.

Longer reads lead to more contiguous assemblies of large genomes, but do not always improve assembly quality

To investigate the effect of increased read length on assembly quality, we simulate Oxford Nanopore, as well as PacBio CLR and HiFi reads with different read length distributions (Table 1). These reads are simulated from the genomes of *S. cerevisiae*, *P. falciparum*, *C. elegans*, *A. thaliana*, *D. melanogaster*, and *T. rubripes* for PacBio CLR and ONT reads and *S. cerevisiae*, *P. falciparum*, *A. thaliana,* and *D. ananassae* for PacBio HiFi reads. We assemble PacBio CLR and ONT reads with Canu, Flye, wtdbg2, Raven, and miniasm and assemble PacBio HiFi reads with HiCanu, Flye, Hifiasm, LJA, and MBG. We evaluate assembly quality based on six evaluation categories (see Overview of the benchmarking pipeline). It is worth mentioning that Canu's PacBio CLR and ONT reads iteration 4 (the longest reads) assemblies of *A. thaliana* and *T. rubripes* did not finish within reasonable time and are excluded from the evaluation.

Figure 5 shows a summary of the assemblers' performance on all simulated read sets, highlighting changes in performance for each read length distribution. All six evaluation metrics are normalized given the maximum and minimum metric values per genome, per sequencing technology, and combined to obtain an average score. For PacBio CLR and ONT read sets, we then average the two resulted scores. Finally we report a rate between 1 and 10 for each assembler, per read length distribution for PacBio CLR and ONT read sets, and a separate score for PacBio HiFi read sets. The results on all computed metrics are fully described in Supplementary Tables S8 – S24.

The results imply that there is a correlation between the size and complexity of the reference genome and the extent of the improvement in assembly quality that can be achieved by increasing the length

of the reads. While we observe no trend in assembly quality improvement on the assemblies of smaller genomes, the results on the *T. rubripes* assemblies are more conclusively in favour of the longer reads. For instance, on the shorter and simpler *S. cerevisiae* and *P. falciparum* genomes, identification of repetitive and complex regions is not aided by increased read length, likely as these regions are already spanned by the reads with default lengths. However, the benchmark results suggest that more complex and repetitive regions within the *A. thaliana*, *D. melanogaster* and, most notably, *T. rubripes* genomes are better captured by longer reads.

As recorded in Supplementary Table S22 and S23, for larger genomes, longer reads generally lead to significantly higher assembly contiguity and a lower misassembly count. The latter implies that the resulting assemblies are more faithful to the references, although this is not necessarily supported by other metrics. We cannot report any compelling improvements in sequence identity, multiplicity, validity, and gene identification.

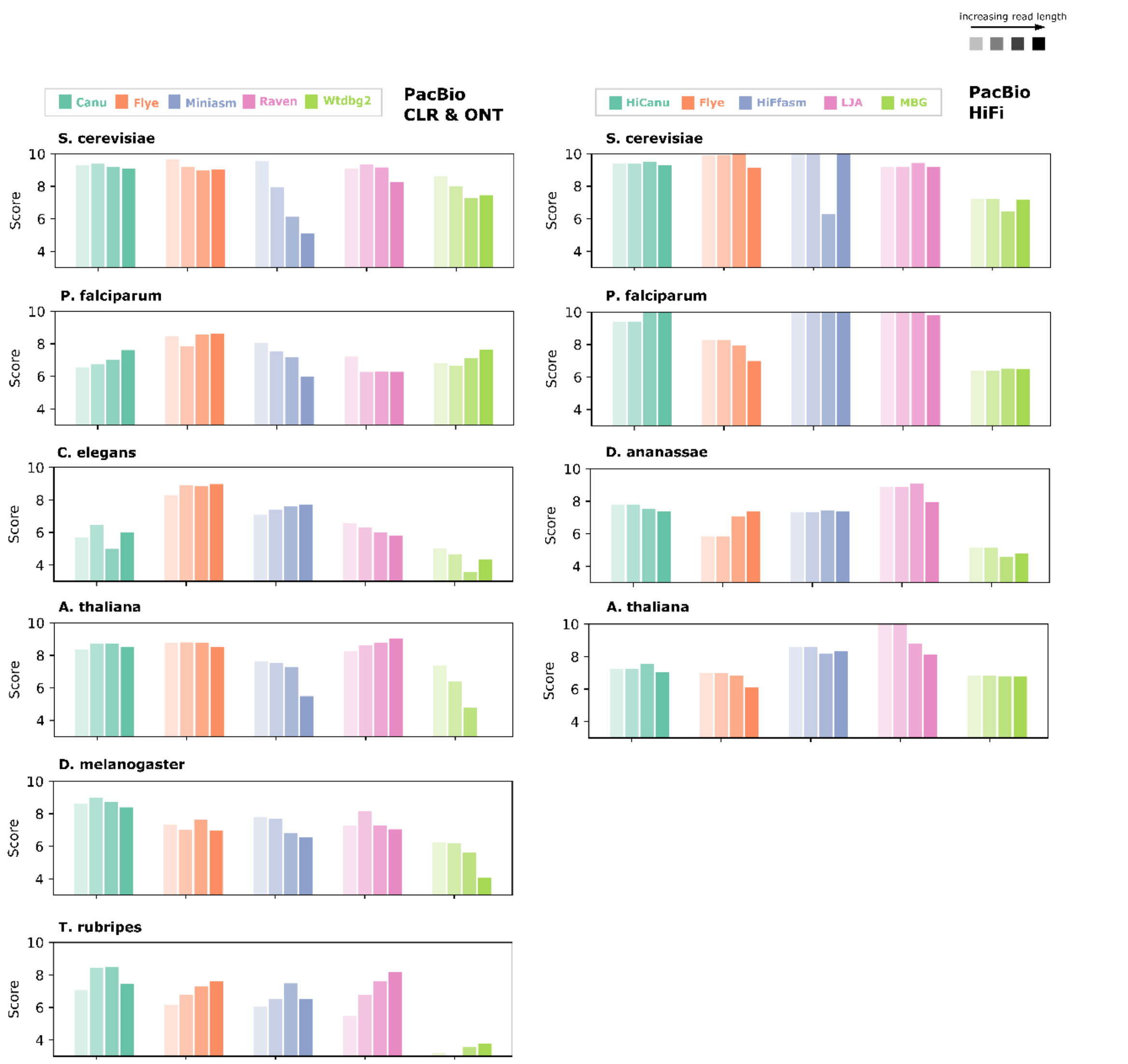

Figure 5: The left panel shows the performance of the five assemblers on all simulated PacBio CLR and ONT read sets, with four different read length distributions (as previously described in Table 1). A score of 1 - 10 is reported for each assembler. The results are normalized for each genome, per sequencing technology. For PacBio CLR and ONT, an average score for each read length distribution is first computed and then these three scores are averaged to obtain an overall score per read length distribution. Similarely, the right panel shows the performance of the five HiFi assemblers on all simulated PacBio HiFi read sets with four different read length distributions.

Conclusion

In fulfilment of the first objective of this study, we conclude that Flye is the highest performing assembler when considering the overview of all evaluation categories in this benchmark, which include the sequence identity, repeat collapse, rate of valid sequences, contiguity, misassembly count, and gene identification. Rankings are mostly consistent for all three sequencing platforms included in the study: PacBio CLR, PacBio HiFi, and ONT. However, no assembler ranks first in all evaluation categories, suggesting that the choice of assembler is often a trade-off between certain advantages and disadvantages. Therefore, we have corroborated the conclusion of Wick and Holt [7], who benchmarked long-read assemblers on prokaryotes, for eukaryotic organisms, and recommend that these benchmarking parameters are considered in relation to the desired outcome of an assembly experiment.

Additionally, the tests performed on real reads validate our rankings of simulated-read assemblies. Flye, the assembler that scored consistently well in most evaluation categories for assemblies of simulated reads in PacBio CLR and ONT datasets, also ranks first when evaluated on several sets of real reads sequenced on long-read platforms.

In our analysis, we found that when processing HiFi reads, both LJA and Hifiasm assemblers showed better performance than other options. While LJA and Hifiasm may not always have been the absolute best, their high performance was a constant, irrespective of the dataset. This was not dataset-specific but was consistently observed in both simulated and real datasets. This underscores their efficiency and accuracy in assembling genomic sequences using HiFi reads.

Regarding our second objective, which is addressing the effect of increased read length on assembly quality, the benchmarking of assemblers on read sets with different read length distributions suggests that longer reads have the potential to improve assembly quality. However, this depends on the size and complexity of the genome that is being reconstructed. We found that improvements in contiguity were most significant among all metrics, as also supported by the conclusion of [8], who showed that

using third generation sequencing considerably improves contiguity in assembling a plant genome (*M. jansenii*). However, we did not find significant improvements in other aspects of assembly quality, such as sequence identity or gene identification.

This study focused on comparison of different sequencing technologies and assemblers on a specific coverage level of 30x, which provided insights into the performance of different assemblers. However, it's important to recognize that assemblers may behave differently at lower or higher coverage levels, and project planners need guidance in selecting the right coverage for their goals and budget. Unfortunately, studying the effect of different coverages on assembly performance is not part of this study.

The field of genomics is continuously evolving, and advancements in sequencing technologies can significantly influence assembly outcomes. While our study focuses on benchmarking long read de novo assembly tools for eukaryotic genomes, the rapid progress in sequencing technologies introduces complexities and challenges in comparing different data types, chemistries, and versions of the tools. In an ideal situation, it would be important to consider all the various factors, including different chemistries, sequencing devices, and base callers when evaluating assemblies. However, due to the limitations of available data and resources, we focused primarily on analyzing the impact of specific chemistry and related factors in this study. We recognize that this represents one of the limitations of our research.

The generations of HiFi reads have witnessed substantial advancements in both read length and accuracy. In earlier versions, HiFi reads typically had read lengths ranging from around 10 to 15 kilobases (kb) with high accuracy rates of 99.9% or greater. However, with subsequent generations, there has been a significant increase in read lengths. The latest versions of HiFi reads now offer read lengths exceeding 20 kilobases, with some reaching up to 30 kilobases or more, while still maintaining high accuracy rates above 99.9%. These longer and highly accurate HiFi reads provide researchers with more contiguous and reliable genomic sequences, enabling improved de novo

assembly and enhancing various genomic analyses. An interesting innovation worth mentioning, while not included in this study, is the introduction of Oxford Nanopore's Duplex reads. This cutting-edge technology holds the potential to enhance sequencing accuracy even further, making it a worthwhile subject for future investigations.

Data availability

All additional supporting data are available in the *GigaScience* repository, GigaDB [35].

Availability of supporting source code and requirements

Our evaluations were produced with QUAST v5.0.2 [31], BUSCO v5.4.2 [33, 34], and COMPASS [13]. We also provide the scripts we used in GitHub:

Project name: Long-read assembly benchmark

Project home page: https://github.com/AbeelLab/long-read-assembly-benchmark

Operating system(s): Platform independent

Programming language: Python and Shell

License: GNU General Public License v3.0

References

1. Boycott KM, Vanstone MR, Bulman DE, MacKenzie AE. Rare-disease genetics in the era of next-generation sequencing: discovery to translation. *Nature Reviews Genetics*. 2013; doi: 10.1038/nrg3555.

2. Bras J, Guerreiro R, Hardy J. Use of next-generation sequencing and other whole-genome strategies to dissect neurological disease. *Nature Reviews Neuroscience*. 2012; doi: 10.1038/nrn3271.

3. Grada A, Weinbrecht K. Next-Generation Sequencing: Methodology and Application. *Journal of Investigative Dermatology*. 2013; doi: 10.1038/jid.2013.248.

4. Schlötterer C, Kofler R, Versace E, Tobler R, Franssen SU. Combining experimental evolution with next-generation sequencing: a powerful tool to study adaptation from standing genetic variation. *Heredity*. 2015; doi: 10.1038/hdy.2014.86.

5. Salazar AN, Gorter de Vries AR, van den Broek M, Wijsman M, de la Torre Cortés P, Brickwedde A, et al.. Nanopore sequencing enables near-complete de novo assembly of Saccharomyces cerevisiae reference strain CEN.PK113-7D. *FEMS Yeast Res*. 2017; doi: 10.1093/femsyr/fox074.

6. Amarasinghe SL, Su S, Dong X, Zappia L, Ritchie ME, Gouil Q. Opportunities and challenges in long-read sequencing data analysis. *Genome Biology*. 2020; doi: 10.1186/s13059-020-1935-5.

7. Wick RR, Holt KE. Benchmarking of long-read assemblers for prokaryote whole genome sequencing. *F1000Research*. 2021; doi: 10.12688/f1000research.21782.4.

8. Murigneux V, Rai SK, Furtado A, Bruxner TJC, Tian W, Harliwong I, et al.. Comparison of long-read methods for sequencing and assembly of a plant genome. *GigaScience*. 2020; doi: 10.1093/gigascience/giaa146.

9. Jung H, Jeon M-S, Hodgett M, Waterhouse P, Eyun S. Comparative Evaluation of Genome Assemblers from Long-Read Sequencing for Plants and Crops. *Journal of Agricultural and Food Chemistry*. 2020; doi: 10.1021/acs.jafc.0c01647.

10. Chen Z, Erickson DL, Meng J. Benchmarking Long-Read Assemblers for Genomic Analyses of Bacterial Pathogens Using Oxford Nanopore Sequencing. *International Journal of Molecular Sciences*. 2020; doi: 10.3390/ijms21239161.

11. Letunic I, Bork P. Interactive Tree Of Life (iTOL) v5: an online tool for phylogenetic tree display and annotation. *Nucleic Acids Research*. 2021; doi: 10.1093/nar/gkab301.

12. Earl D, Bradnam K, John JS, Darling A, Lin D, Fass J, et al.. Assemblathon 1: A competitive assessment of de novo short read assembly methods. *Genome Research*. 2011; doi: 10.1101/gr.126599.111.

13. Bradnam KR, Fass JN, Alexandrov A, Baranay P, Bechner M, Birol I, et al.. Assemblathon 2: evaluating de novo methods of genome assembly in three vertebrate species. *GigaScience*. 2013; doi: 10.1186/2047-217X-2-10.

14. Dijk EL van, Auger H, Jaszczyszyn Y, Thermes C. Ten years of next-generation sequencing technology. *Trends in Genetics*. 2014; doi: 10.1016/j.tig.2014.07.001.

15. Wick R. Badread: simulation of error-prone long reads. *JOSS*. 2019; doi: 10.21105/joss.01316.

16. Nurk S, Koren S, Rhie A, Rautiainen M, Bzikadze AV, Mikheenko A, et al.. The complete sequence of a human genome. *Science*. 2022; doi: 10.1126/science.abj6987.

17. Li H. Minimap2: pairwise alignment for nucleotide sequences. Birol I, editor. *Bioinformatics*. 2018; doi: 10.1093/bioinformatics/bty191.

18. Koren S, Walenz BP, Berlin K, Miller JR, Bergman NH, Phillippy AM. Canu: scalable and accurate long-read assembly via adaptive *k* -mer weighting and repeat separation. *Genome Research*. 2017; doi: 10.1101/gr.215087.116.

19. Kolmogorov M, Yuan J, Lin Y, Pevzner PA. Assembly of long, error-prone reads using repeat graphs. *Nat Biotechnol*. 2019; doi: 10.1038/s41587-019-0072-8.

20. Ruan J, Li H. Fast and accurate long-read assembly with wtdbg2. *Nature Methods*. 2020; doi: 10.1038/s41592-019-0669-3.

21. Vaser R, Šikić M. Time- and memory-efficient genome assembly with Raven. *Nature Computational Science*. 2021; doi: 10.1038/s43588-021-00073-4.

22. Li H. Minimap and miniasm: fast mapping and de novo assembly for noisy long sequences. *Bioinformatics*. 2016; doi: 10.1093/bioinformatics/btw152.

23. Nurk S, Walenz BP, Rhie A, Vollger MR, Logsdon GA, Grothe R, et al.. HiCanu: accurate assembly of segmental duplications, satellites, and allelic variants from high-fidelity long reads. *Genome Res*. 2020; doi: 10.1101/gr.263566.120.

24. Cheng H, Concepcion GT, Feng X, Zhang H, Li H. Haplotype-resolved de novo assembly using phased assembly graphs with hifiasm. *Nat Methods*. 2021; doi: 10.1038/s41592-020-01056-5.

25. Bankevich A, Bzikadze AV, Kolmogorov M, Antipov D, Pevzner PA. Multiplex de Bruijn graphs enable genome assembly from long, high-fidelity reads. *Nat Biotechnol*. 2022; doi: 10.1038/s41587-022-01220-6.

26. Rautiainen M, Marschall T. MBG: Minimizer-based sparse de Bruijn Graph construction. *Bioinformatics*. 2021; doi: 10.1093/bioinformatics/btab004.

27. Chen Z, Erickson DL, Meng J. Polishing the Oxford Nanopore long-read assemblies of bacterial pathogens with Illumina short reads to improve genomic analyses. *Genomics*. 2021; doi: 10.1016/j.ygeno.2021.03.018.

28. Hu T, Chitnis N, Monos D, Dinh A. Next-generation sequencing technologies: An overview. *Human Immunology*. 2021; doi: 10.1016/j.humimm.2021.02.012.

29. Wick RR, Holt KE. Polypolish: Short-read polishing of long-read bacterial genome assemblies. *PLOS Computational Biology*. 2022; doi: 10.1371/journal.pcbi.1009802.

30. Vaser R, Sović I, Nagarajan N, Šikić M. Fast and accurate de novo genome assembly from long uncorrected reads. *Genome Research*. 2017; doi: 10.1101/gr.214270.116.

31. Gurevich A, Saveliev V, Vyahhi N, Tesler G. QUAST: quality assessment tool for genome assemblies. *Bioinformatics*. 2013; doi: 10.1093/bioinformatics/btt086.

32. Barthelson R, McFarlin AJ, Rounsley SD, Young S. Plantagora: Modeling Whole Genome Sequencing and Assembly of Plant Genomes. *PLoS ONE*. 2011; doi: 10.1371/journal.pone.0028436.

33. Simão FA, Waterhouse RM, Ioannidis P, Kriventseva EV, Zdobnov EM. BUSCO: assessing genome assembly and annotation completeness with single-copy orthologs. *Bioinformatics*. 2015; doi: 10.1093/bioinformatics/btv351.

34. Waterhouse RM, Seppey M, Simão FA, Manni M, Ioannidis P, Klioutchnikov G, et al.. BUSCO Applications from Quality Assessments to Gene Prediction and Phylogenomics. *Molecular Biology and Evolution*. 2018; doi: 10.1093/molbev/msx319.

35. Cosma B, Shirali Hossein Zade R, Jordan EN, Lent Pv, Peng C, Pillay S, et al. Supporting data for "Evaluating Long Read De Novo Assembly Tools for Eukaryotic Genomes: Insights and Considerations" *GigaScience Database*. 2023. http://dx.doi.org/10.5524/102425.

Click here to access/download
Supplementary Material
SupplementaryMaterials.docx

Click here to access/download
Supplementary Material
Supplementary_Tables_S25-S31.xlsx

Cover letter Click here to access/download;Personal Cover;cover_letter.pdf

Contact: Dr. T. Abeel
Phone: +31 15 27 85 114
E-mail: t.abeel@tudelft.nl

TU Delft

Faculty of Electrical Engineering, Mathematics and Computer Science

Department of Intelligent Systems
Pattern Recognition and Bioinformatics

Van Mourik Broekmanweg 6
2628XE Delft
Netherlands

Delft, January 10, 2023

Dear Editor,

We are pleased to submit the enclosed review article: “When do longer reads matter? A benchmark of long read de novo assembly tools for eukaryotic genomes” for consideration in *GigaScience*.

De novo genome assembly is essential in several leading fields of research, including disease identification, gene identification, and evolutionary biology. Since it is not constrained to the use of a reference, high quality *de novo* assembly is essential for studying novel organisms, as well as for the discovery of overlooked genomic features, such as gene duplication.

The introduction of third-generation sequencing technology, lead to the development of lots of tools that aim to take advantage of the longer reads to achieve better genome assembly quality. In this study, we benchmark 5 state-of-the-art long-read *de novo* assembly tools using 13 real and 72 simulated datasets from 6 different eukaryotic genomes. These datasets are from both PacBio and Oxford Nanopore, the two widely used third-generation sequencing platforms. We evaluate the assemblies using 6 different metrics, to obtain an exhaustive view of the assemblies. This benchmark will be invaluable to support scientists to identify the right tools to use for their research as well as provide the outline for systematic benchmark of assembly software going into the future.

We confirm that all authors fulfil authorship criteria and have read the final version of the manuscript.

Kind regards,

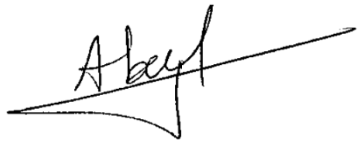

Thomas Abeel
Associate Professor Bioinformatics
Group leader Computational Microbiology
Delft Bioinformatics Lab

Response letter

Click here to access/download;Personal Cover;Response_letter.docx

Dear editor and reviewers,

We sincerely appreciate the thoughtful comments and suggestions you provided for our manuscript. We've strived to address all your queries and incorporate your feedback as much as feasible within our study's purview. We're confident that these modifications have enriched our manuscript significantly. Enclosed, please find our responses to your queries, marked in blue, alongside the original questions in black.

Reviewer reports:

Reviewer #1: Overall, this manuscript is well-written and understandable. There's a lot of good work here and I think the authors were thoughtful about how to compare the resulting assemblies. Scripts and models used have been made available for free via GitHub and could be mirrored on or moved to GigaDB if required. I'll include a several minor comments, including some line-item edits, but the bulk of my comments will focus on a few major items.

Major Comments:

My primary concern here is that the comparison is outdated and doesn't address some of the most helpful questions. CLR-only assemblies are no longer state-of-the-art. There are still applications and situations where ONT (simplex, older-pore)-only assemblies are reasonable, but most projects that are serious about generating excellent assemblies as references are unlikely to take that approach. Generating assemblies for non-reference situations, especially when the sequencing is done "in the field" (e.g., using a MinION with a laptop) or by a group with insufficient funding or other access to PromethIONs and Sequel/Revios, is an exception to this for ONT-only assemblies. Further, this work assumes a person wants to generate "squashed" assemblies instead of haplotype-resolved or pseudohaplotype assemblies. To be fair, sequencing technology in the TGS space has been advancing so rapidly that it is extremely difficult to keep up, and a sequencing run is often outdated by the time analyses are finished, not to mention by the time a manuscript is written, reviewed, and published. Accordingly, in raising my concerns, I am not objecting to the analysis being published or suggesting that the work performed was poor, but I do believe clarifications and discussion are necessary to contextualize the comparison and specify what is missing.

1. This comparison seeks to address Third-generation sequencing technologies: namely PacBio vs. ONT. However, each company offers multiple kinds of long-read sequencing, and they are not all comparable in the same way. Just as long noisy reads (PacBio CLR & ONT simplex) are a whole new generation from "NGS" short reads like from Illumina, long-accurate reads are arguably a new generation beyond noisy long reads. If this paper wants to include PacBio HiFi reads in the comparison, significant changes are necessary to make the comparison meaningful. I think it's reasonable to drop HiFi reads from this paper altogether and focus on noisy long reads since the existing comparison isn't currently set up to tell us enough about HiFi reads and including them would be an ordeal. If including HiFi, consider the following:

1.a. Use assemblers designed for long-accurate reads. HiCanu (i.e., Canu with --pacbio-hifi option) is already used, as is a similar approach for Flye and wtdbg2. However, raven is not meant for HiFi data and miniasm is not either (though, it could be done with the correct minimap2 settings, but Hifiasm would be better). Assemblies of HiFi data with Raven and miniasm should be removed. Sidenote - Raven can be run with --weaken (or similar) for HiFi data, but it is only experimental and the parameter has since been removed. Including Hifiasm would be necessary, and it should have been included since Hifiasm was out when this analysis was done. Similarly, including MBG (released before your analysis was done) would be appropriate. Since you'd be redoing the analyses, it would be appropriate to include other assemblers that have since been released: namely LJA. Once could argue that Verkko should be included, but that opens another can of worms as a hybrid assembler (more on that later).

1b. Use a read simulator that is built for HiFi reads. Badreads is not built for HiFi data (though using custom parameters to make it work for HiFi reads wasn't a bad idea at the time), and new simulators (e.g., PBSIM3, DOI: 10.1093/nargab/lqac092) have since been released that consider the multi-pass process used to generate HiFi data.

1c. ONT Duplex data is likely not available for the species you've chosen as it is a _very_ new technology. However, you should at least discuss its existence as something for readers to "keep an eye on" as something that is conceptually comparable to HiFi.

1d. Use the latest & greatest HiFi data if possible and at least discuss the evolution of HiFi data. Even better would be to compare HiFi data over time, but this data may not really be available and most people won't be using older HiFi data. Though, simulation of older data would conceivably be possible. While doing so would make this paper more complete, I would argue that it isn't worth the effort at this juncture. For reference, in my observation, older data has a median read length around 10-15 kb instead of 18-22 kb.

1e. Include real Hifi data for the species you are assembling. If none is available and you aren't in a position to generate it, then keep the hifi assembler comparison on real data separate from that of the CLR/ONT assembler comparisons on real data by using real HiFi data for other species.

Thank you for your insightful comments and suggestions regarding the inclusion of PacBio HiFi reads in the comparison. We acknowledge the importance of considering the distinct characteristics of different long-read sequencing technologies. Following your suggestion, we encountered challenges in finding HiFi reads for the species under evaluation, which led us to separate the HiFi assembler comparison from the CLR/ONT assemblers. To address this, we acquired publicly available HiFi datasets and performed assemblies using four assemblers for five organisms *S. cerevisiae, A. Thaliana, D. ananassae,* and *P. falciparum*. Additionally, we employed PBSIM3 to generate simulated HiFi reads, enhancing the evaluation of the final assemblies. We have also incorporated a dedicated section in the paper to discuss ONT duplex reads and generations of HiFi reads. While we strived to address these aspects, we appreciate your suggestions for using assemblers specifically designed for HiFi reads and utilizing read simulators built for HiFi data. Due to the constraints of available data and the scope of the study, incorporating the latest advancements in HiFi technology and species-specific HiFi data may present challenges.

“The generations of HiFi reads have witnessed substantial advancements in both read length and accuracy. In earlier versions, HiFi reads typically had read lengths ranging from around 10 to 15 kilobases (kb) with high accuracy rates of 99.9% or greater. However, with subsequent generations, there has been a significant increase in read lengths. The latest versions of HiFi reads now offer read lengths exceeding 20 kilobases, with some reaching up to 30 kilobases or more, while still maintaining high accuracy rates above 99.9%. These longer and highly accurate HiFi reads provide researchers with more contiguous and reliable genomic sequences, enabling improved de novo assembly and enhancing various genomic analyses. An interesting innovation worth mentioning, while not included in this study, is the introduction of Oxford Nanopore's Duplex reads. This cutting-edge technology holds the potential to enhance sequencing accuracy even further, making it a worthwhile subject for future investigations.”

2. Discuss in the intro and/or discussion that you are focusing on "squashed" assemblies. Without clever sample separation and/or trio-based approaches (e.g., DOI: 10.1038/nbt.4277), a single squashed haplotype is the only possible outcome for PacBio CLR and ONT-only approaches. For non-haploid genomes, other approaches (HiFi-only or hybrid approaches (e.g., HiFi + ONT or HiFi + Hi-C)) can generate pseudohaplotypes at worse and fully-resolved haplotypes at best. The latter is an objectively better option when possible, and it's important to note that this comparison wouldn't apply when planning a project with such goals. Similarly, it would probably be helpful to point out to the novice reader that this comparison doesn't apply to metagenome assembly either.

We added the following paragraph to the introduction to specify that we are focusing on “squashed” assemblies.

“It is important to note that our objective is to evaluate the performance of these tools in generating a consensus assembly without taking haplotypes into account. Moreover, It is crucial to highlight that the results and conclusions drawn from this comparison may not be directly applicable to metagenome assembly. The unique characteristics and complexities associated with metagenomic data warrant a separate and distinct analysis, which is beyond the scope of this study.”

3. The title suggests to the reader that we'll be shown how long reads makes a difference in assembly compared to non-long read approaches. However, this is not the case, despite some mention of it in near line 318. Short read assemblies are not compared here and no discussion is provided to suggest how long read-based assemblies would improve outcomes in various situations relative to short reads. Unless such a comparison and/or discussion is added, I think the title should be changed. I've included this point in the "Major Comments" section because including such a comparison would be a big overhaul, but I don't expect this to be done. The core concern is that the analysis is portrayed correctly.

We changed the title to:

“Evaluating Long Read De Novo Assembly Tools for Eukaryotic Genomes: Insights and Considerations”

Done

4. Sequencing technologies are often portrayed as static through time, but this is not accurate. This is a failing of the field generally. Part of the problem is the length of the publishing cycle (often >1yr from when a paper is written to when it's published, not to mention how long it takes to do the analysis before a paper is even written). Part of the problem is that current statistics are often cited in influential papers and then recited in more recent papers based on the influential paper despite changes having

been made since that influential paper was released. Accordingly, the error rate in ONT reads has been misreported as being ~15% for many years even though their chemistry has improved over time and the machine learning models (especially for human samples) have also improved, dropping the error rate substantially. ONT has made improvements to their chemistry and changed nanopores over time and PacBio has tinkered with their polymerase and chemistry too.

Accordingly, a better question for a person planning to perform an assembly would be "which assembler is best for my datatype (pacbio clr vs ont) and chemistry/etc.?" instead of just differentiating by company. Any comparison of those datatypes should at least address this as a factor in their discussion, if not directly in their analysis. I feel that this is missing from this comparison. In an ideal world, we'd have various CLR chemistries and ONT pores/etc. for each species in this analysis. That data likely doesn't exist for each of the chosen species though, and generating it would be non-trivial, especially retroactively. Using the most recent versions is a good option, but may also not exist for every species chosen. Since this analysis was started (circa Nov/Dec 2021 by my estimate based on the chosen assembler versions), ONT has released pore 10; in combination with the most recent release of Guppy, error rates <=3% are expected for a huge portion of the data. That type of data is likely to assemble very differently from R9.4, and starker differences would be expected for data older than R9.4. Even if all the data were the most recent (or from the same generation (e.g., R9.4)), library preps vary greatly, especially between UL (ultralong) libraries and non-UL libraries. Having reads >100kb, especially a large number of them, makes a big difference in assembly outcome in my observation. How does choice of assembler (and possibly different parameters) affect the assembly when UL data is included? How is that different from non-UL data? What about UL data at different percentages of the reads being considered UL? A paper focusing on long noisy reads would be much more impactful if it addresses these questions. Again, this may not be possible for this particular paper considering what's already been done and the available funding, and I think that's okay. However, these issues need to addressed in the discussion as open questions and suggested future work. The type of CLR and ONT data also needs to be specified in this work, e.g., in a supplemental table, and if the various datasets are not from the same types, these differences need to be acknowledged. At a minimum, I think the following data points should be included: chemistry/pore information (e.g., R9.4 for ONT or P2/C5 for PacBio), basecaller (e.g., guppy vX.Y.Z), and read length distribution info (e.g., mean, st. dev., median, %>100kb), ideally a plot of the distribution in addition to summary values. I also understand that these data were generated previously by others, and this information should theoretically be available from their original publications, which are hopefully accessible via the INSDC records associated with the provided accessions. The objective here is making the information easily accessible to the readers of this paper because those could be confounding variables in the analysis.

Thank you for your valuable comment. We made an effort to incorporate the requested information into the manuscript. However, due to limitations such as restricted access to certain manuscripts and unavailability of the information on the NCBI website, we were not able to find requested information. Consequently, we were unable to include the requested table. To address your comment, we have included the following paragraph in the discussion section.

"The field of genomics is continuously evolving, and advancements in sequencing technologies can significantly influence assembly outcomes. While our study focuses on benchmarking long read de novo assembly tools for eukaryotic genomes, the rapid progress in sequencing technologies introduces complexities and challenges in comparing different data types, chemistries, and versions of the tools. In an ideal situation, it would be important to consider all the various factors, including different chemistries, sequencing devices, and base callers when evaluating assemblies. However, due to the limitations of available data and resources, we focused primarily on analyzing the impact of specific

chemistry and related factors in this study. We recognize that this represents one of the limitations of our research."

5. This comparison considered only a single coverage level (30x). That's not an unreasonable shortcut, but it certainly leaves a lot of room for differences between assemblers. If the objective the paper is to help future project planners decide what assembler to use, it would be most helpful if they had an idea of what coverage they can use and still succeed. That's especially true for projects that don't have a lot of funding or aren't planning to make a near-perfect reference genome (which would likely spend the money on high coverage of multiple datatypes). It would be helpful to include some discussion about how these results may be different at much lower (e.g., 2x or 10x coverage) or at higher coverage (e.g., 50x, 70x, etc.) and/or provide some justification from another study for why including that kind of comparison would be unlikely to be worthwhile for this study, even if project planners should consider those factors when developing their budget and objectives.

We add the following paragraph to the conclusion section.

"This study focused on comparison of different sequencing technologies and assemblers on a specific coverage level of 30x, which provided insights into the performance of different assemblers. However, it's important to recognize that assemblers may behave differently at lower or higher coverage levels, and project planners need guidance in selecting the right coverage for their goals and budget. Unfortunately, studying the effect of different coverages on assembly performance is not part of this study."

6. Figure 2 and 3 include a lot of information, and I generally like how they look and that they provide a quick overview. I believe two things are missing that will improve either the assessment or the presentation of the information, and I think one change will also improve things.

6a. I think metrics from Merqury (DOI: 10.1186/s13059-020-02134-9) should be included where possible. Specifically, the k-mer completeness (recovery rate) and reference-free QV estimate (#s 1 and 3 from https://github.com/marbl/merqury/wiki/2.-Overall-k-mer-evaluation). Generally these are meant to be done from data of the same individual. However, most of the species selected for this comparison are highly homozygous strains that should have Illumina data available, and thus having the data come from not the exact some individual will likely be okay. This can serve as another source of validation. If such a dataset is not available for 1 or more of these species, then specify in the text that it wasn't available, and thus such an evaluation wasn't possible. If it's not possible to add one or both of these metrics to the figures (2 & 3), that's fine, but having it as a separate figure would still be helpful. I find these values to be some of the most informative for the quality of an assembly.

We appreciate your suggestion to include Merqury metrics for a more comprehensive evaluation. However, securing Illumina data for each species, which is necessary for this analysis, presents considerable challenges. Moreover, including these metrics is beyond the intended scope of our current study since it already includes so much information. We'll keep your suggestion in mind for future research considerations.

6b. It's not strictly necessary, so this might be more of a minor comment, but I found that I wanted to view individual plots for each metric. Perhaps including such plots in the supplement would help (e.g., 6 sets of plots similar to figure 4 with color based on assembler, grouping based on species, and opacity

based on datatype). The specifics aren't critical, I just found it hard to get more than a very general idea from the main figures and wanted something easy to digest for each metric.

We appreciate your feedback. However, we believe the current figure provides a comprehensive view of the effects of increasing read lengths, This visualization strategy allows for insights without becoming overly complex. While additional individual plots could offer more specific data points, they may not necessarily enhance the overall understanding, given the breadth of information already presented in the existing figure.

6c. Using N50/NG50 for a measure of contiguity is an outdated and often misleading approach. Unfortunately, it's become such common practice that many people feel obligated to include it or use it. Instead, the auN (auNG) would be a better choice for contiguity: https://lh3.github.io/2020/04/08/a-new-metric-on-assembly-contiguity.

Based on the reviewer comment, we replaced NG50 with auNG to measure contiguity.

7. This paper focuses on assembly and intentionally does not consider polishing (line 176), which I think is a reasonable choice. It also does not consider scaffolding or hybrid assembly approaches (again, reasonable choices). In the case of hybrid assembly options, most weren't available when this analysis was done (short read + long read assemblers were available, but I think it's perfectly reasonable to not have included those). Given the frequency of scaffolding (especially with Hi-C data [DOIs: 10.1371/journal.pcbi.1007273 & 10.1093/bioinformatics/btac808]) and the recent shift to hybrid assemblers (e.g., phasing HiFi-based string graphs using Hi-C data to get haplotype resolved diploid assemblies (albeit with some switch errors) [DOI: 10.1038/s41587-022-01261-x] or resolving HiFi-based minimizer de bruijn graphs using ONT data and parental Illumina data to get complete, T2T diploid assemblies [DOI: 10.1038/s41587-023-01662-6]), I think it would be appropriate to briefly mention these methods so the novice reader will know that this benchmark does not apply to hybrid approaches or post-assembly genome finishing. This is a minor change, but I included it in this section because it matches the general theme of ensuring the scope of this benchmark is clear.

This research deeply investigates assembly, a fundamental part in the field of genomic research. It's important to note that this study doesn't explore other processes like polishing, scaffolding, or the increasingly common hybrid assembly methods. While methods involving Hi-C data for scaffolding and innovative hybrid assemblers are interesting areas of research, they are not the focus of this paper's benchmark. Therefore, the findings and insights of this benchmark do not apply to these advanced techniques or to the final stages of genome assembly. This benchmark is a clear representation of a specific area within the ever-changing field of genome assembly.

Minor Comments:

1. line 25 in the abstract. Change Redbean to wtdbg2 for consistency with the rest of the manuscript.

Done.

2. "de novo" should be italicized. It is done correctly in some places but not in others.

Done.

3. line 64. "all TGS technologies": I would argue that this isn't quite true. ONT Duplex isn't included here even though Duplex likely didn't exist when you did this work. Also, see the major comments concerning whether TGS should include HiFi and Duplex.

Changed all TGS technologies to most used TGS technologies.

4. Table 1. Read length distributions vary dramatically by technology and library prep. E.g., HiFi is often a very tight distribution about the mean because of size selection. Including the median in the table would be helpful, but more importantly, I would like to see read-length distribution plots in the supplement for (a) the real data used to generate the initial iteration models and (b) the real data from each species.

We changed the HiFi simulator from Badread to PBSIM3, which doesn't require a model for simulation. However, we added read length distributions of all real datasets the reviewer requested to the supplementary materials.

5. line 166 "fair comparison". I'm not sure that a fair comparison should be the goal, but having them at the same coverage level makes them more comparable which is helpful. Maybe rephrase to indicate that keeping them at the same coverage level reduces potentially confounding variables when comparing between the real and simulated datasets.

Done.

6. line 169. Citation 18 is used for Canu, which is appropriate but incomplete. The citation for HiCanu should also be included here: DOI: 10.1101/gr.263566.120.

We added the citation for HiCanu.

7. line 169. State that these were the most current releases of the various assemblers at the time that this analysis was started. Presumably, that was Nov/Dec 2021. Since then, Raven has gone from v1.7.0->1.8.1 and Flye has gone from v2.9->2.9.1.

Done.

8. line 175. Table S6 is mentioned here, but S5 has not yet been mentioned. S5 is mentioned for the first time on line 196. These two supp tables' numbers should be swapped.

Done.

9. There is inconsistent use of the Oxford comma. I noticed is missing multiple times, e.g., lines 191, 208, 259, & 342.

Done.

10. line 193. The comma at the end of the line (after "tools") should be removed. Alternatively, keep the comma but add a subject to the next clause to make it an independent clause (e.g., "...assembly tools, and they were computed...").

Done.

11. line 237. The N50 of the reference is being used here. You provide accessions for the references used, but most people will not go look those up (which is reasonable). The sequences in a reference can vary greatly in their lengths, even within the same species, because which sequences are included in the reference are not standardized. Even the size difference between a homogametic and heterogametic reference can be non-trivial. Which are included in the reference, and more importantly included in your N50 value, can significantly change the outcome and may bias results if these are not done consistently between the included species. It would be helpful if here or somewhere (e.g., in some supplemental text or a table) the contents of these references was somehow summarized. In addition to 1 copy of each of the expected autosomes, were any of the following included: (a) one or two sex chromosomes if applicable, (b) mitochondrial, chloroplast, or other organelle sequences, (c) alternate sequences (i.e., another copy of an allele of some sequence included elsewhere), (d) unplaced sequence from the 1st copy, (e) unplaced sequence from subsequent copies, and (f) vectors (e.g., EBV used when transforming a cell line)?

Thanks for your insightful comments. We recognize that reference genome details can impact N50 values. But our study's primary focus is on the assembly process. An in-depth report on each reference genome, including aspects like sex chromosomes, organelle sequences, and others, could be valuable. However, it's not within our study's current scope, which emphasizes the impact of read length on assembly performance. So, we've kept our focus on N50 values of reference genomes, which we've included in Supplementary Table S1.

12. Supplemental tables. Some cells are uncolored, and other cells are colored red or blue with varying shading. I didn't notice a legend or description of what the coloring and shading was supposed to mean. Please include this either with each table or at the beginning of the supplemental section that includes these tables and state that it applies to all tables #-#.

We removed the coloring since it was not providing any extra information.

13. Supplemental table S3. It was not clear to me that you created your own model for the hifi data (pacbio_hifi_human2022). I was really confused when I couldn't find that model in the GitHub repo for Badreads. In the caption for this table or in the text somewhere, please make it more explicit that you created this yourself instead of using an existing model.

We decided to use PBSIM3 for simulating HiFi reads and we are not using the model for the assemblies generated for the revised manuscript. We also made it more explicit in the caption of table S3.

Reviewer #2: Dear Cosma and colleagues,

Your manuscript, which I had the pleasure of reading, is, simply put, a benchmark of five long read de novo assembly tools. Using 13 real and 72 simulated datasets, the manuscript evaluated the performance of five widely used long-read de novo assemblers: Canu, Flye, Miniasm, Raven, and Redbean.
Although I find the methodological approach of the manuscript to be solid and trustworthy, I do not think the research is particularly innovative. Long-read assemblers have already been benchmarked in the scientific literature, and similar findings have been made. The authors are aware of this limitation of the study and have added a novel feature: the impact of read length on assembly quality, which in my opinion is still lacking sufficient innovation. However, the manuscript as a whole is valid and worthy of consideration. In light of this, I would like to share some suggestions I made in an effort to make the manuscript unique and more novel. Please see my comment below.

1) Evaluation of the assemblies

The metrics used to evaluate an assembly are frequently a murky subject as we are still lacking a standard language. The authors assessed the assemblies using three types of metrics: compass analysis, assembly statistics, and the Busco assessment, in addition to computational metrics like runtime and RAM usage. This is not incorrect, but I would suggest making a clear distinction between the metrics using (in addition to the computational metrics) three widely recognised metrics, or in short, the 3C criterion. The assembly metrics can be broken down into three dimensions: correctness (your compass analysis), contiguity (NG50) and completeness (the BUSCO assessment). The authors should reconsider the text using the 3C criterion; this will provide a clear, understandable, and structured way of categorising metrics. The paragraph beginning at line 197, for example, causes some confusion for the reader. The NG50 metrics evaluate assembly contiguity, whereas the number of misassemblies (considered by the authors in terms of relocation, inversion, and translocation) evaluate assembly correctness. I must admit that the misassemblies and contiguity can overlap, but I would still recommend keeping the NG50 (within contiguity) and misassemblies (within correctness) metrics separate.

We appreciate your recommendation. Following your advice, we've categorized the metrics according to the 3C criterion. Furthermore, we've replaced NG50 with auNG, a better metric for evaluating contiguity.

2) Novelty of the comparison

The authors of the study had two main goals: to conduct a systematic comparison of five long-read assembly tools (Raven, Flye, Wtdbg2 or Redbean, Canu, and Miniasm) and to determine whether increased read length has a positive effect on overall assembly quality.
The authors acknowledge the study's limitations and include an evaluation of the effect of read length on assembly quality as a novel feature of the manuscript (see line 70).

The manuscript that described the Raven assembler (Vaser, R., Sikic, M. Time- and memory-efficient genome assembly with Raven. Nat Comput Sci 1, 332-336 (2021)) compared the same assemblers' tools (Raven, Flye, Wtdbg2 or Redbean, Canu and Miniasm) evaluated in this manuscript plus two more (Ra and Shasta), used similar eukaryotes (A. thaliana, D. melanogaster, and Human), and reached a similar conclusion on Flye in terms of contiguity (NG50), and completeness (genome fraction) but overall there is not a best assembler in all of the evaluated categories. In this manuscript authors increased the number of eukaryotic genomes (including S. cerevisiae, C. elegans, T. rupribes, and P. falciparum) and reached similar conclusions: there is no assembler that performs the best in all the evaluation categories, but overall Flye is the best-performing assembler. This strengthens the manuscript, but the research is not entirely novel. Given that the field of third-generation technologies is rapidly progressing toward the generation of high-quality reads (Pacbio HiFi technology and ONT Q20+ chemistry are achieving accuracy of 99% and higher), the manuscript should also include a HiFi assembler benchmark. This would add novelty to the manuscript and pique the scientific community's interest.
The authors have already simulated HiFi reads from S. cerevisiae, P. falciparum, C. elegans, A. thaliana, D. melanogaster, T. rubripes in addition to reference reads (or real reads) from S. cerevisiae (SRR18210286). P. falciparum (SRR13050273) and A. thaliana (SRR14728885). Furthermore, I am not sure what the benefit is of evaluating Canu on HiFi data instead of HiCanu, which was designed to deal with HiFi data. The authors already included some HiFi-enabled assemblers like Flye and Wtdbg2 but also HiFiasm should also be considered.

I would strongly advise benchmarking the HiFi assemblers to complete the study and add a level of novelty. I would like to emphasise that the manuscript is solid and that I appreciate it; however, I believe that some novelty should be added.

To address reviewer's suggestion, we expanded our benchmarking to include five HiFi assemblers: HiCanu (previously referred to as Canu), Flye in HiFi mode, hifiasm, mbg, and lja. We evaluated these assemblers using simulated HiFi datasets real read HiFi datasets. Both real and simulated reads were used to evaluate the performance of these assemblers. To improve the manuscript's readability, we decided to present the HiFi assembly comparison separately from the ONT and CLR assembly comparisons. This approach allows for clearer organization and facilitates easier understanding of the results.

3) C elegans genomics the now-discontinued RSII, which had a higher error rate and a shorter average read than Sequel I or Sequel II, was used to generate the genomic data from C elegans. I understand the authors' motivation for including it in the analysis, but the use of RSII may skew the comparisons, and I would suggest adding a few sentences to the discussion about it.

We appreciate your concerns regarding the use of RSII-generated genomic data for C. elegans. Although the RSII technology has indeed been discontinued and it had a higher error rate and shorter average read than Sequel I or II, we felt it was important to include it in the analysis for completeness. We added the following sentence in the manuscript to address the comment.

"Please note that the PacBio CLR data from C. elegans was generated using the older RSII technology. These reads inherent characteristics of the RSII system, such as shorter average reads and a higher error rate, which might have influenced the assembly results."

4) CPU time (h) and memory usage

The authors claim the benchmark evaluation included runtime and RAM usage. However, I missed finding information about the runtime and RAM usage.

Please provide CPU time (h) and memory usage (GB)

All the information about runtime and RAM usage was provided in the Supplementary Tables S25-S30.

Minor comments:

----

1) Lines 64-65

"Here, we provide a comprehensive comparison on de novo assembly tools on all TGS technologies and 7 different eukaryotic genomes, to complement the study of Wick and Holt"
I would modify "on all TGS technologies" as "at the present the two main TGS technologies"

We changed it to the most used TGS technologies.

2) Line 163

Real reads. The term "real reads" may cause confusion for readers, leading them to believe that the authors produced the sequencing reads for the manuscript. I would use the term "ref-reads" indicating "reads from the reference genomes".

Thank you for your feedback. We appreciate your perspective, and we acknowledge that the term "real reads" can potentially be confusing. However, it's important to note that the term "real reads" is used frequently in the literature to signify authentic sequencing data. For instance, a study by Wick et al. (2021) (doi.org/10.12688/f1000research.21782.4) uses "real reads" to distinguish between actual sequencing data and simulated data.

3) Lines 218-219

Please provide full names (genus + species):

S. cerevisiae, P. falciparum, A. thaliana, D. melanogaster, C. elegans, and T. Rubripes

We added the full names.

4) Supplementary Table S4 "Accession number SRR15720446 seems to belong to a sample sequenced with 1 PACBIO_SMRT (Sequel II) rather than ONT

Thanks for noticing this. The correct accession number is SRR16149191.

5) Figures 2 and 3.

Figures 2 and 3 give visual results of the performance of the five assemblers. I want to make a few points here:

According to what I understand, the top-performing assembler is marked with a star and is plotted with a brighter colour than the others. However, this is not immediately apparent, and some readers might have trouble identifying the colour that has been highlighted. I would suggest either lessening the intensity of the other, lower-performance assemblers or giving the best assembler a graphically distinct outline.

I also wonder if it would be useful to give the exact numbers as supplemental tables.

Following the reviewer's suggestion, we decreased the intensity of the radar plot of other assemblers and gave a distinct outline to the best assembler.

The exact numbers were already present in supplemental tables S8-S24.
