# Supplementary material for: Evaluating long-read de novo assembly tools for eukaryotic genomes: insights and considerations
Source: Gigascience. 2023 Nov 24;12:giad100. doi: 10.1093/gigascience/giad100 (PMC10673639; doi:10.1093/gigascience/giad100)
Supplement: giad100_Supplemental_File [file giad100_supplemental_file.zip › SupplementaryMaterials.docx]

When do longer reads matter? A benchmark of long read de novo assembly tools for eukaryotic genomes

Bianca-Maria Cosma^1^, Ramin Shirali Hossein Zade^1^, Erin Noel Jordan^1,2^, Paul van Lent^1^, Chengyao Peng^1^, Stephanie Pillay^1^, and Thomas Abeel^1,3,*^

^1^Delft Bioinformatics Lab, Delft University of Technology Van Mourik, Broekmanweg 6, 2628 XE, Delft, The Netherlands; ^23^Technical Biochemistry, TU Dortmund University, Emil-Figge-Straße 66, 44227, Dortmund, Germany; and ^4^Infectious Disease and Microbiome Program, Broad Institute of MIT and Harvard, 415 Main Street, Cambridge, MA, 02142, USA

*t.abeel@tudelft.nl

# Supplementary Materials


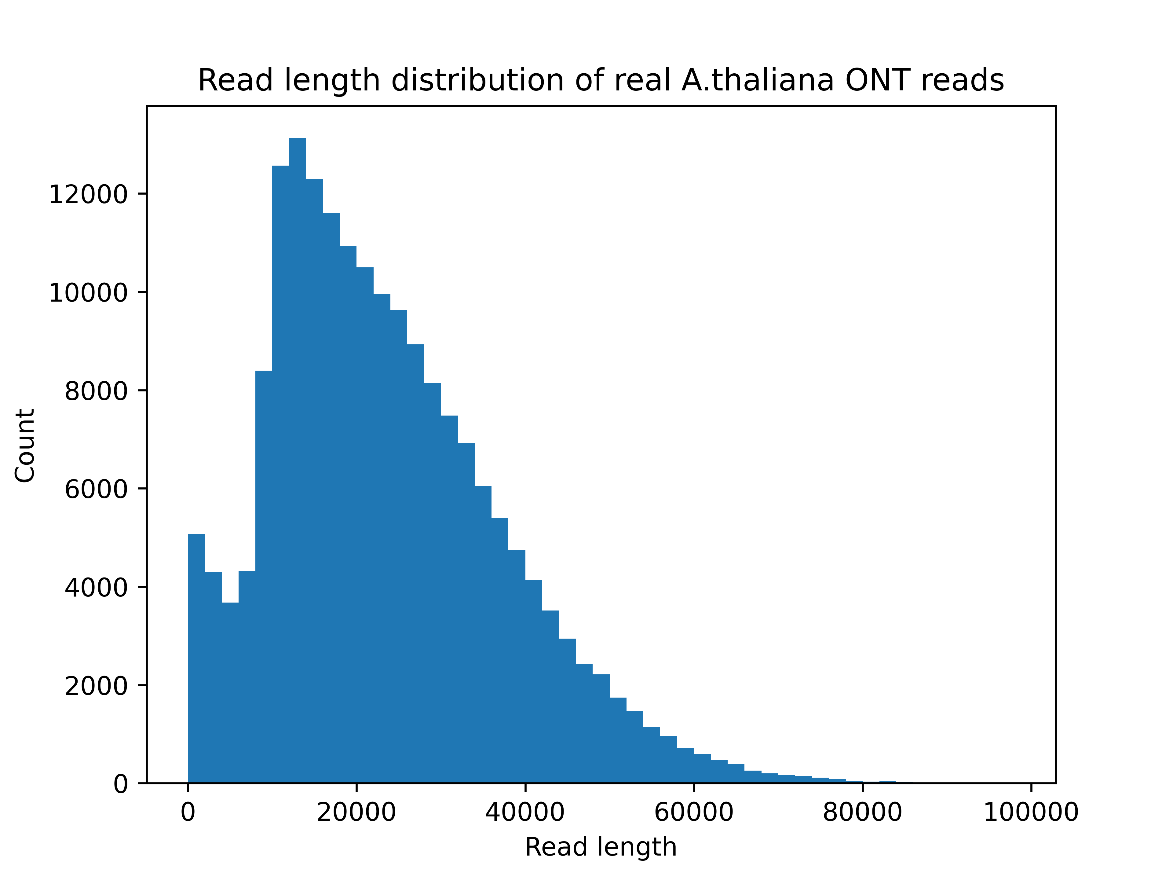


**Supplementary Figure S1: Read length distribution of real A.thaliana ONT reads**

**
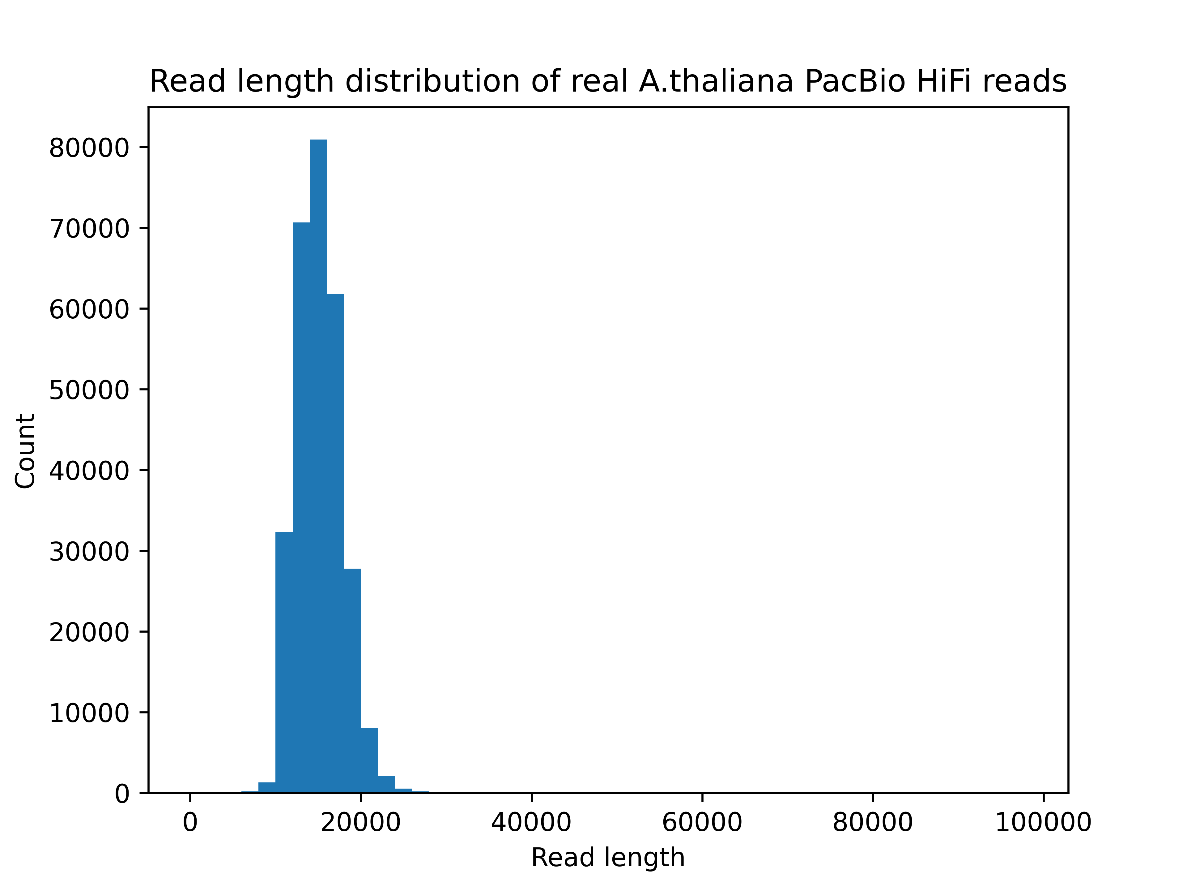
**

**Supplementary Figure S2: Read length distribution of real A.thaliana PacBio HiFi reads**

**
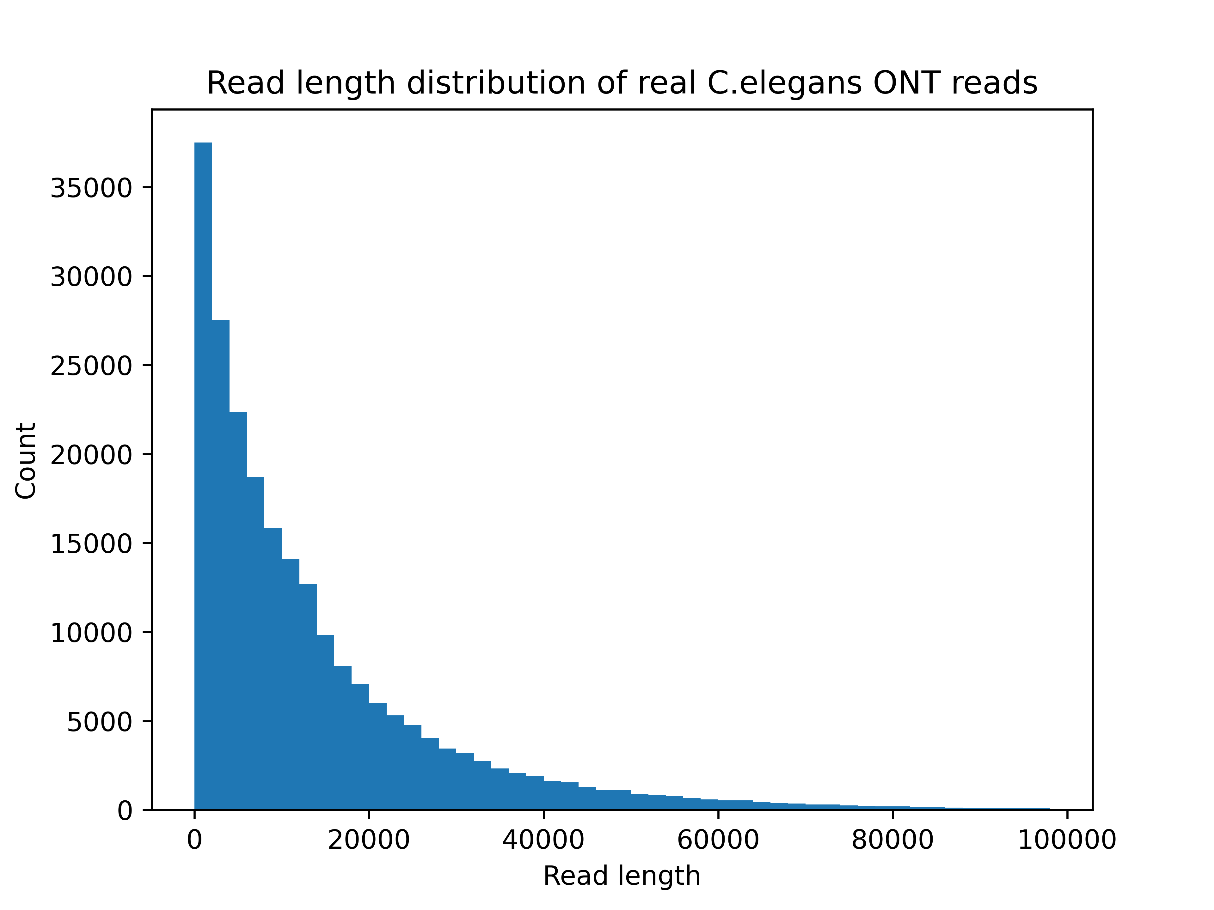
**

**Supplementary Figure S3: Read length distribution of real C.elegans ONT reads**

**
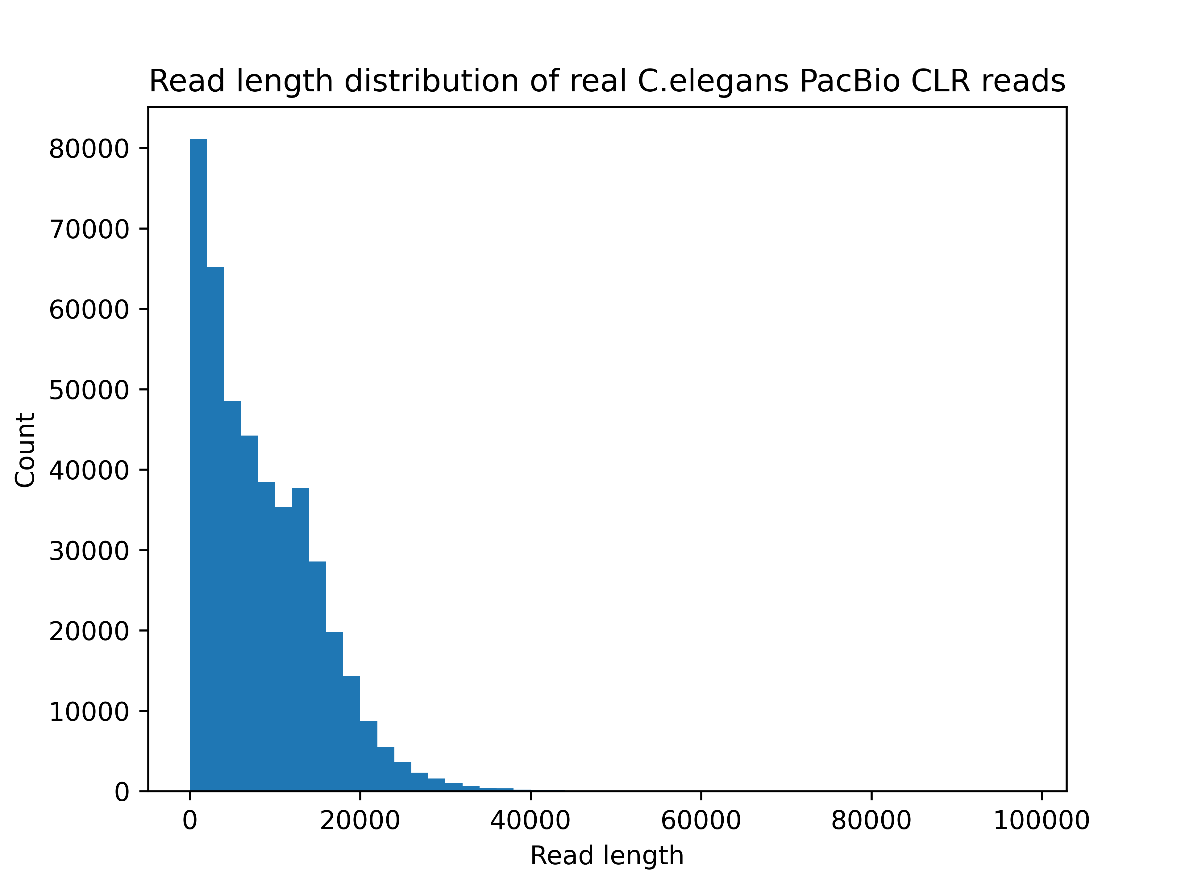
**

**Supplementary Figure S4: Read length distribution of real C.elegans PacBio CLR reads**

**
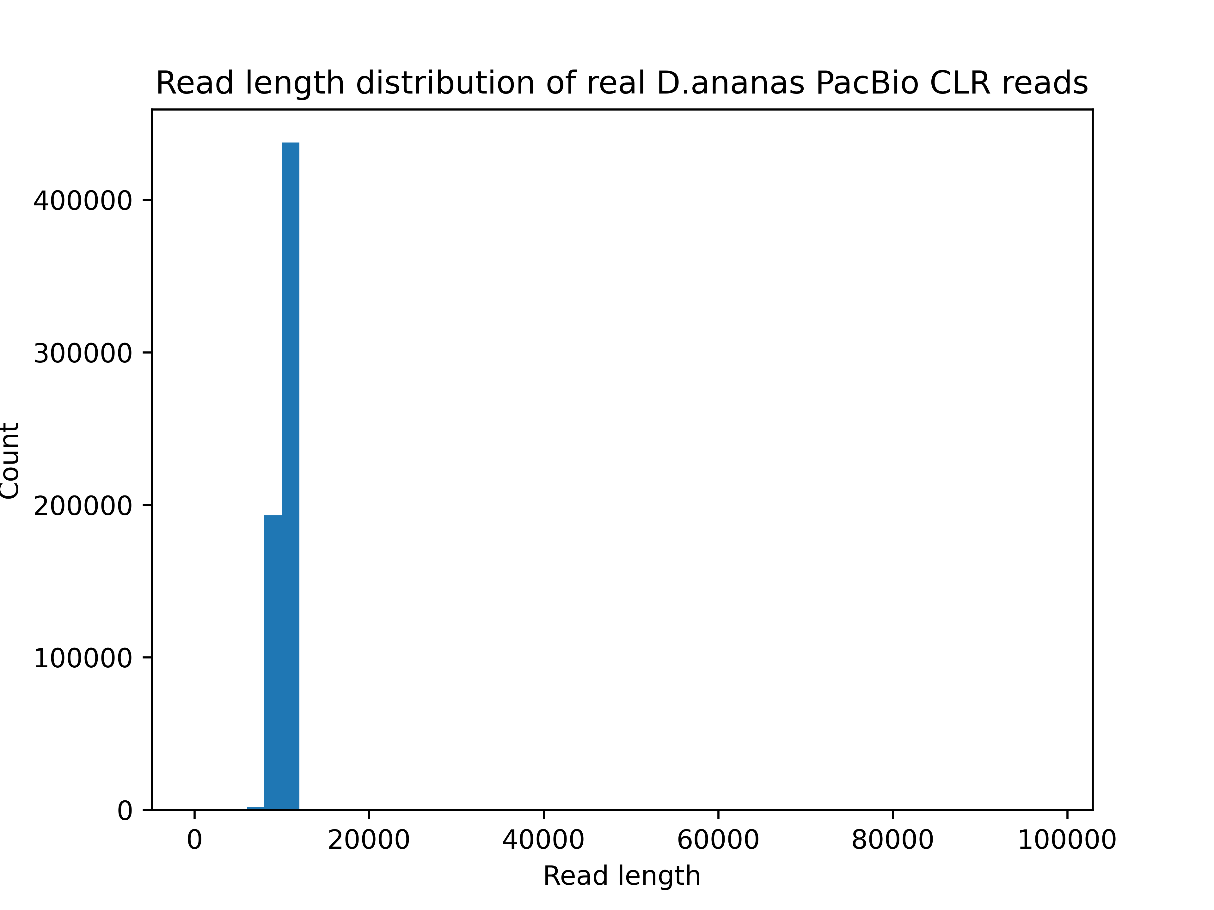
**

**Supplementary Figure S5: Read length distribution of real D.ananas PacBio CLR reads**

**
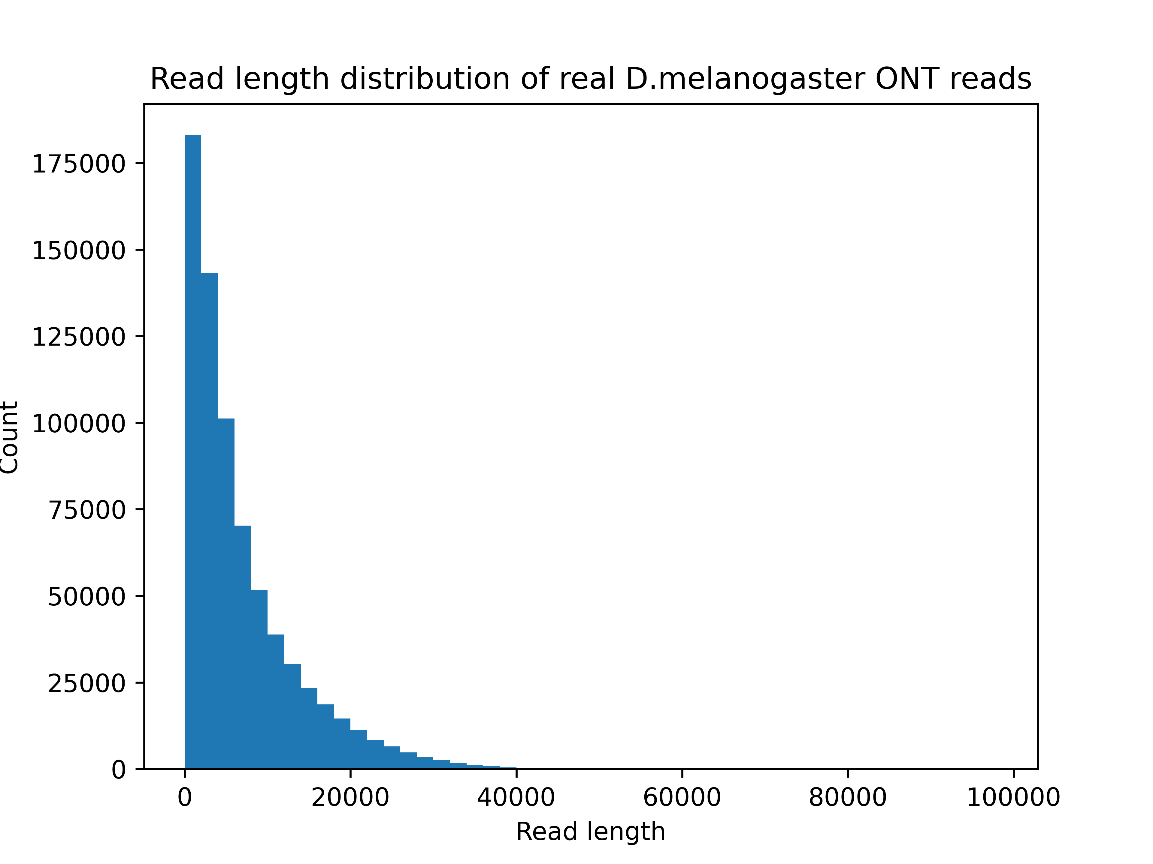
**

**Supplementary Figure S6: Read length distribution of real D.melanogaster ONT reads**

**
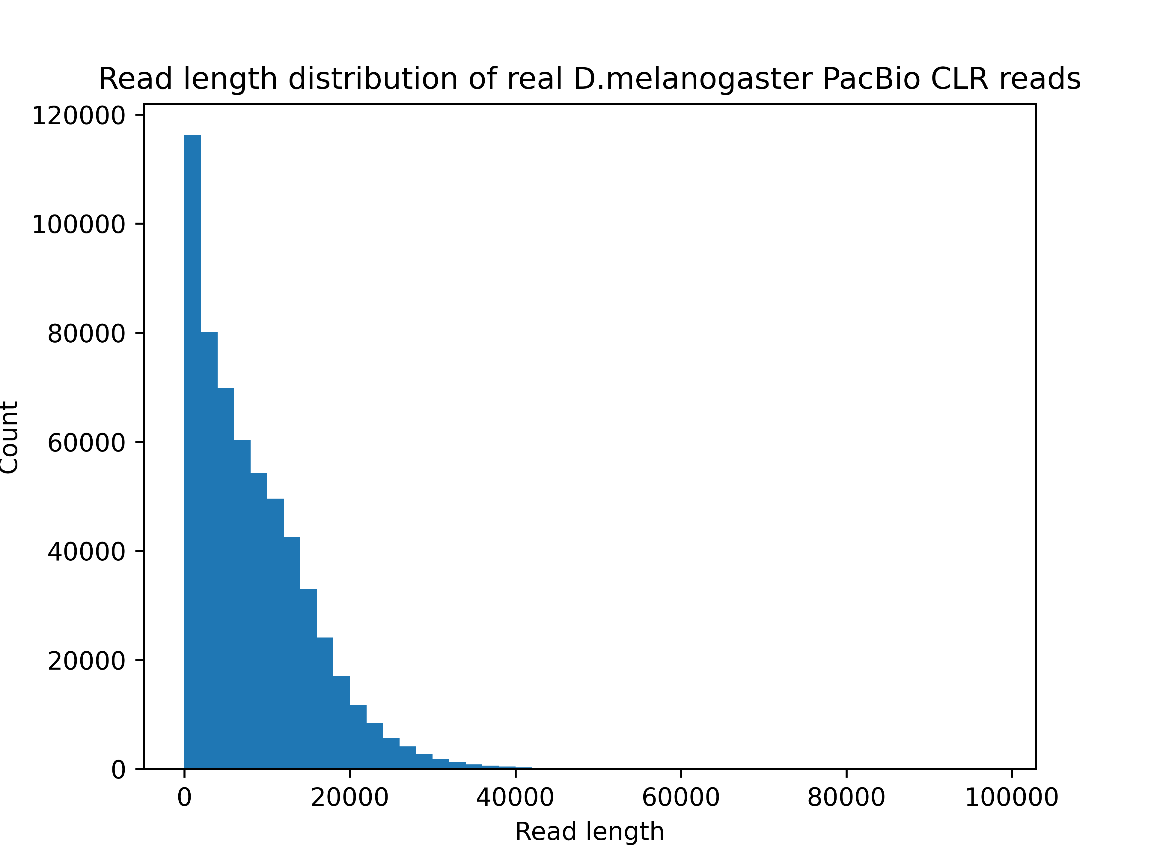
**

**Supplementary Figure S7: Read length distribution of real D.melanogaster PacBio CLR reads**

**
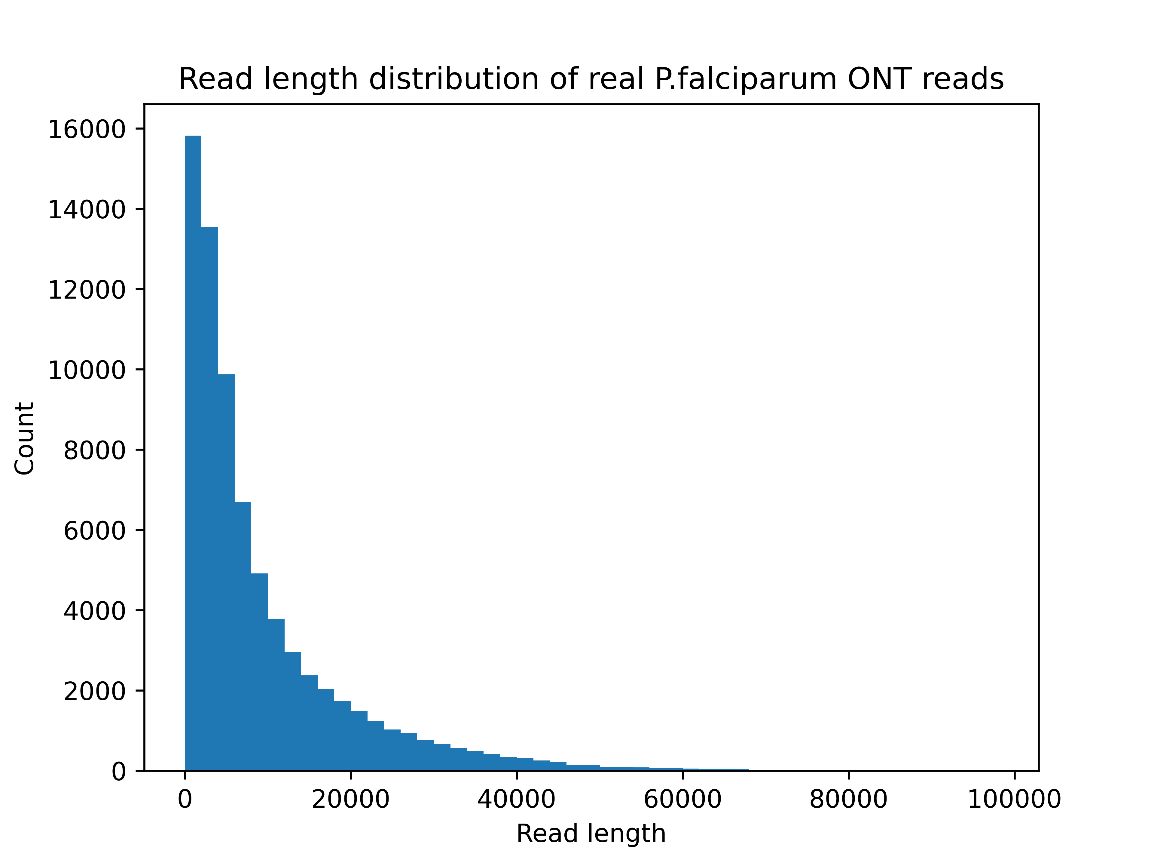
**

**Supplementary Figure S8: Read length distribution of real P.falciparum ONT reads**

**
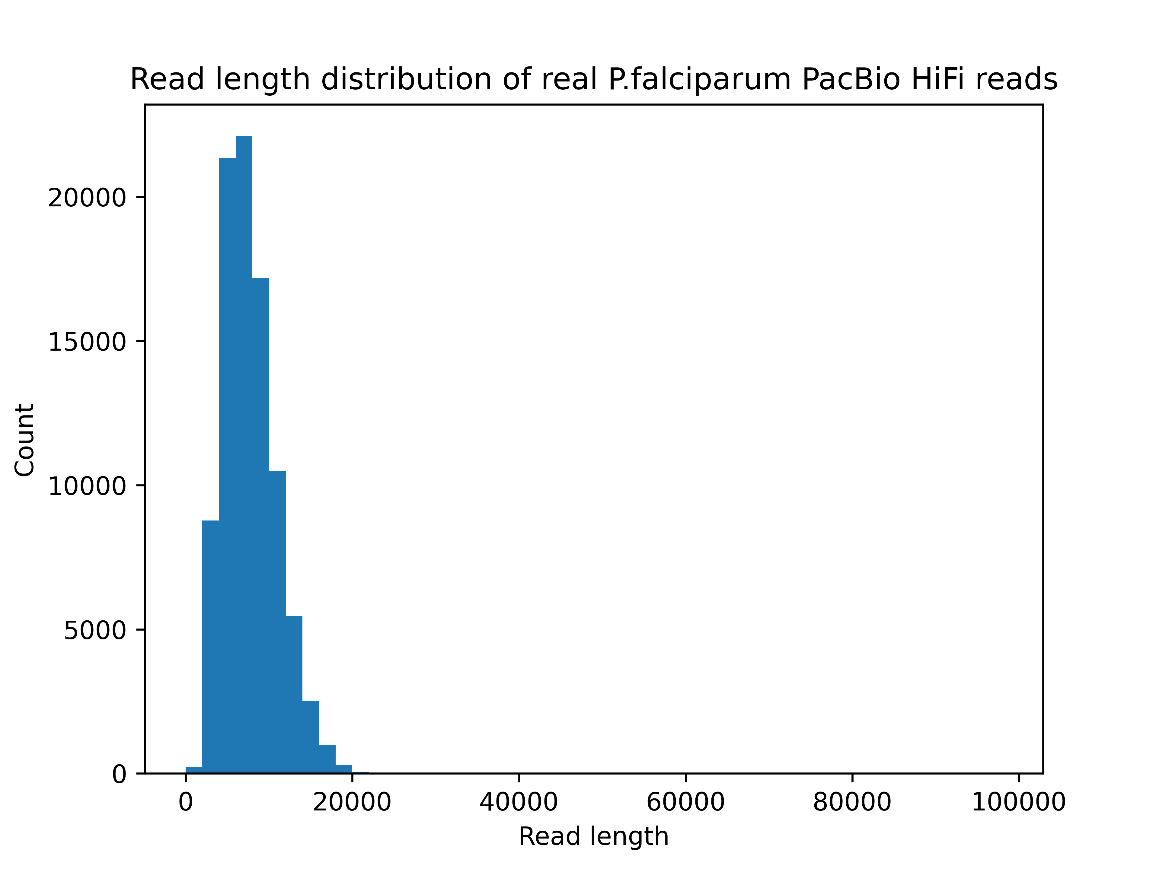
**

**Supplementary Figure S9: Read length distribution of real P.falciparum PacBio HiFi reads**

**
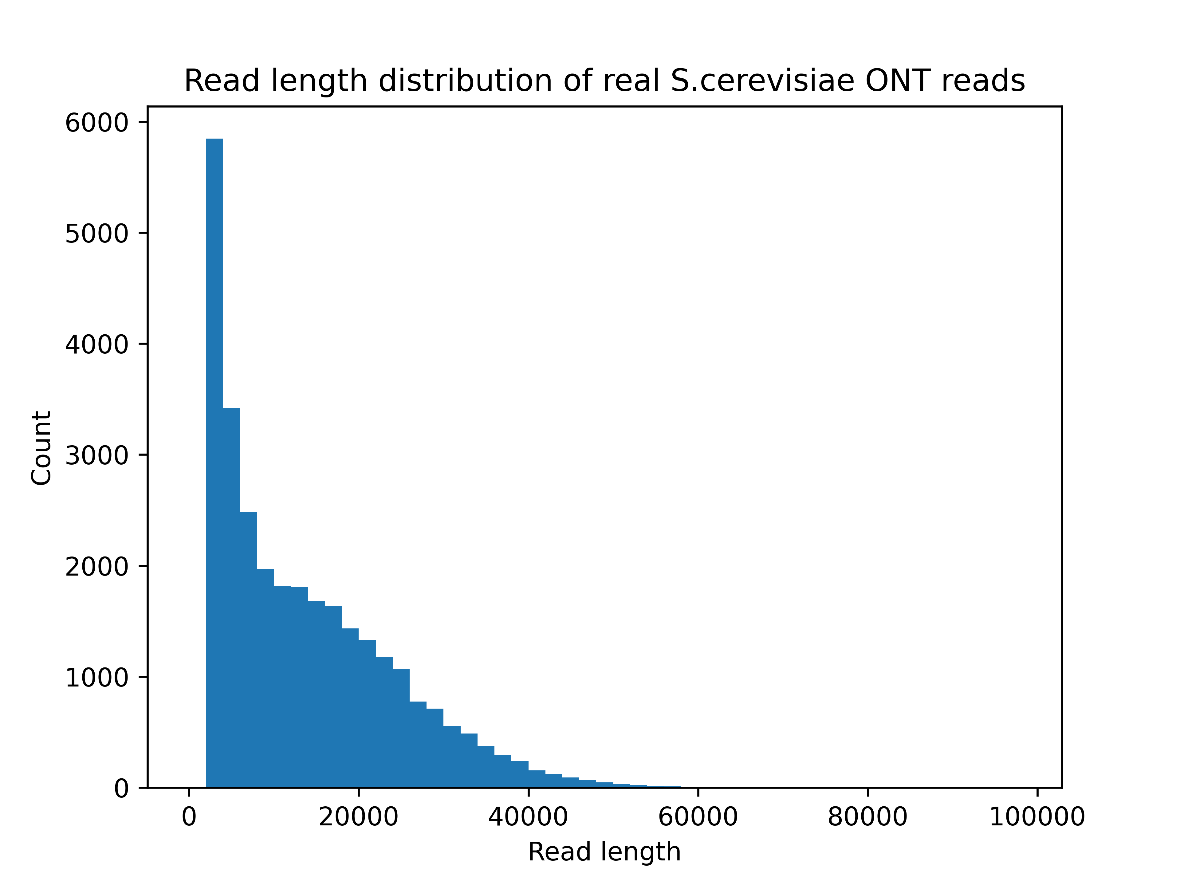
**

**Supplementary Figure S10: Read length distribution of real S.cerevisiae ONT reads**

**
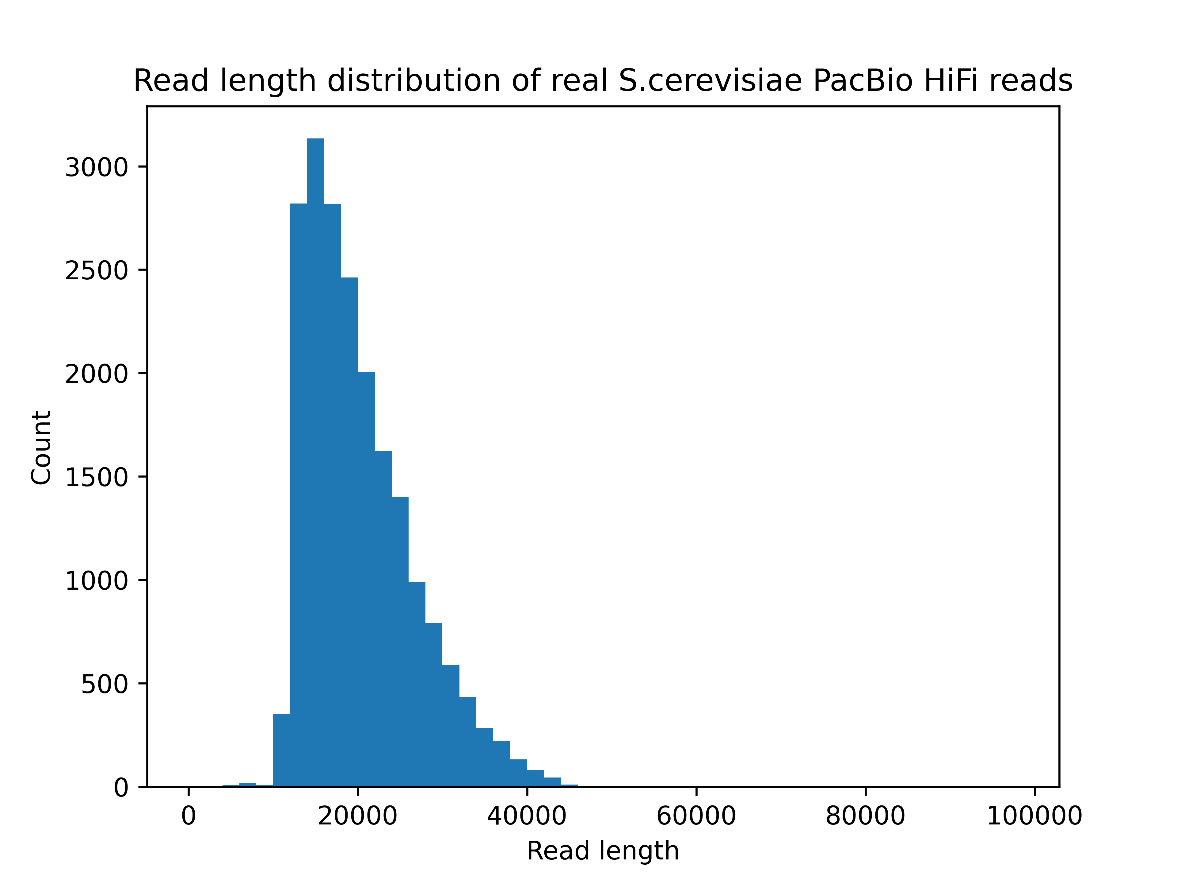
**

**Supplementary Figure S11: Read length distribution of real S.cerevisiae PacBio HiFi reads**

**
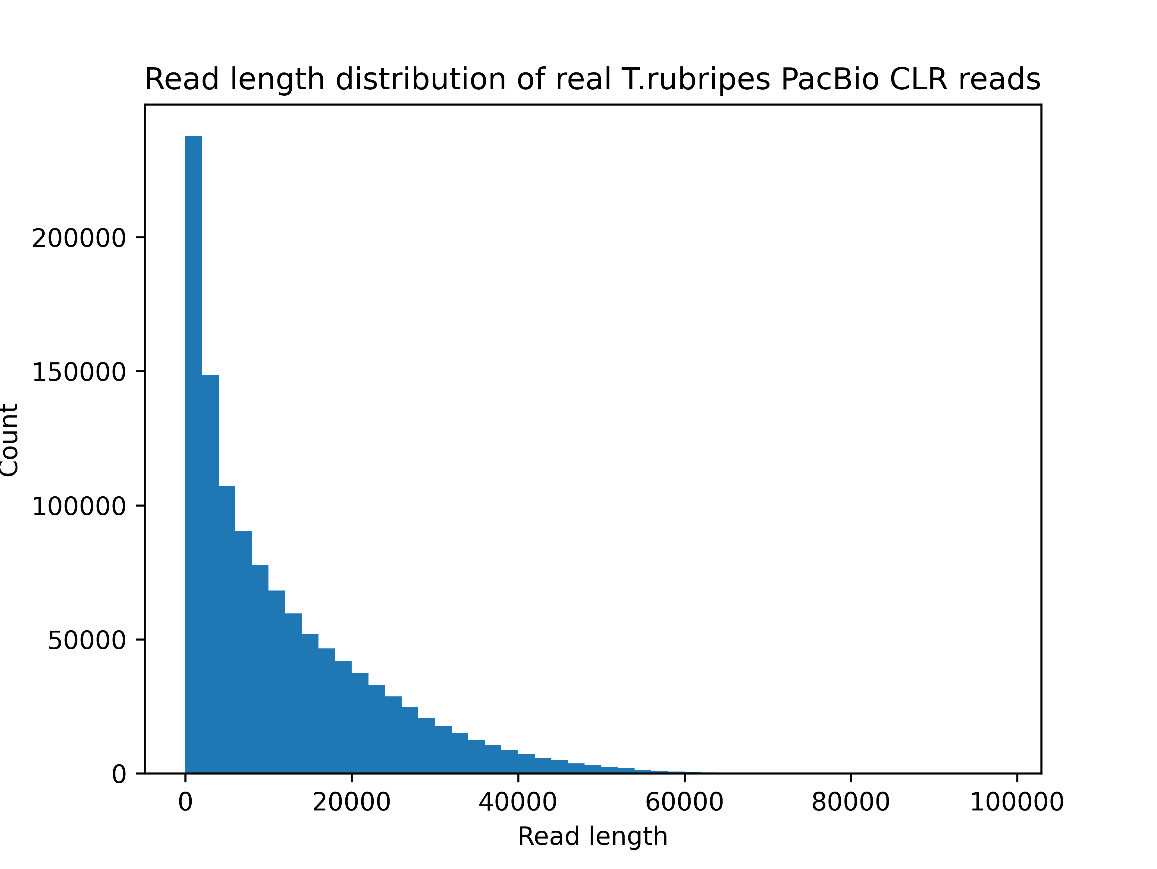
**

**Supplementary Figure S12: Read length distribution of real T.rubripes PacBio CLR reads**


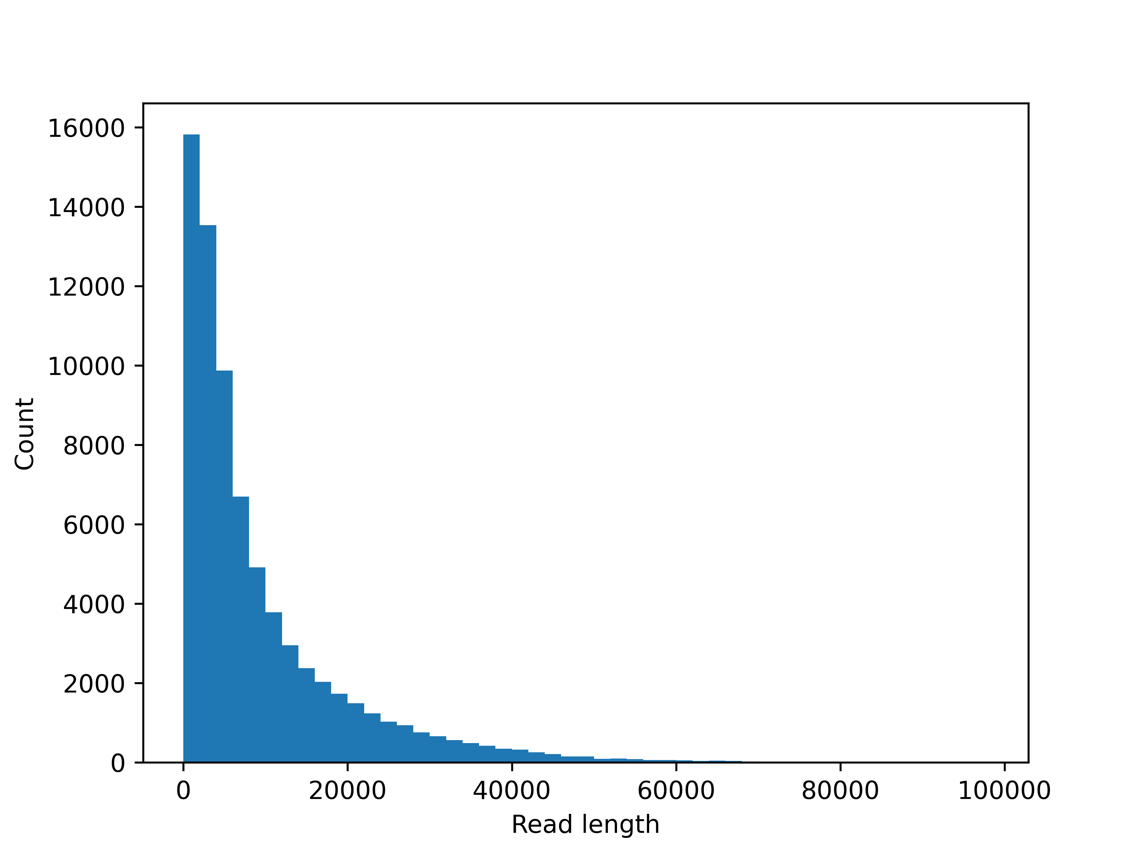


**Supplementary Figure S13: Read length distribution of real Human PacBio CLR reads**

**
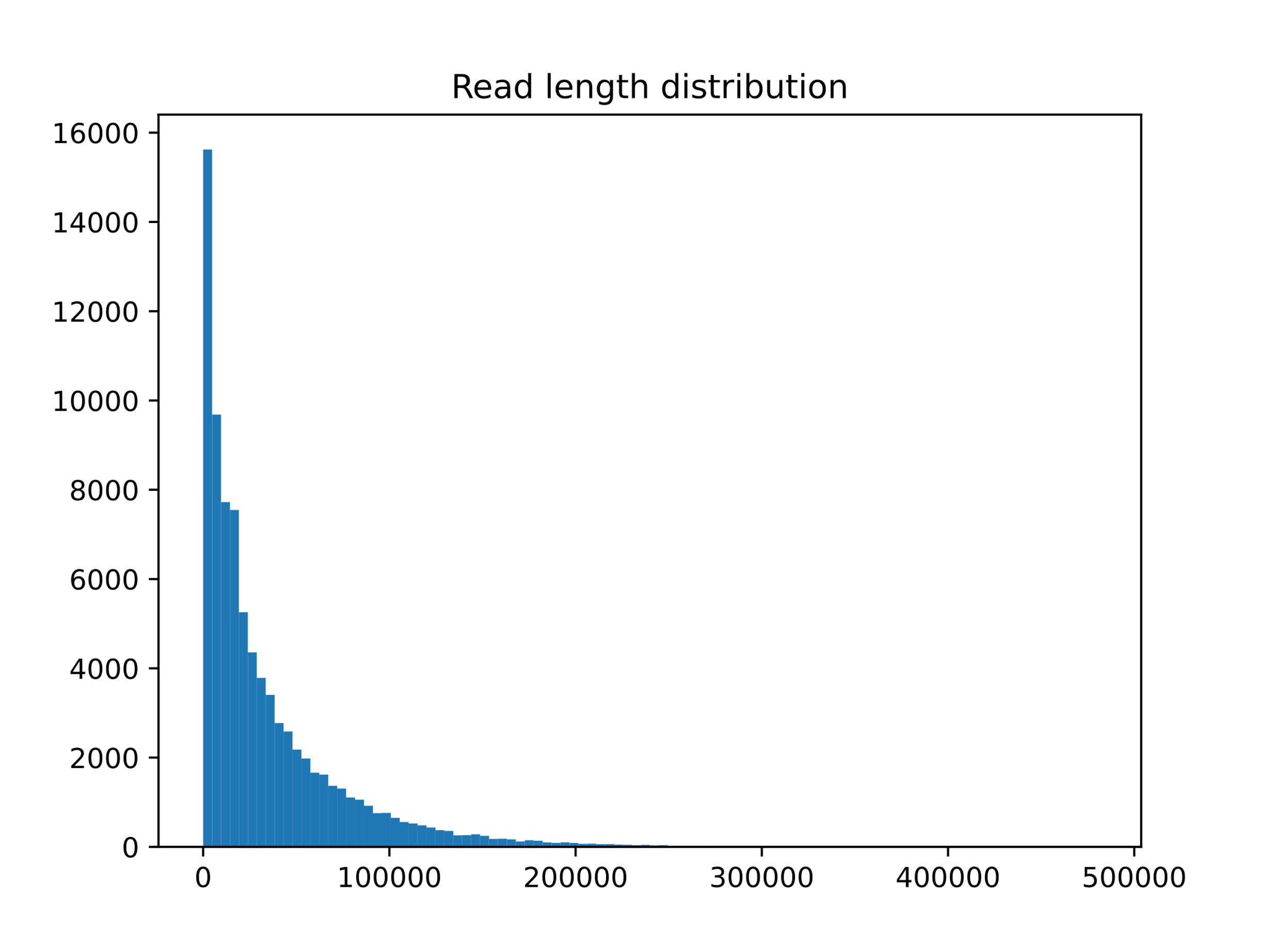
**

**Supplementary Figure S14: Read length distribution of real Human ONT reads**

**Supplementary Table S1:** Assembly accession numbers for all seven reference genomes used in the experiments.

| **Organism** | **Reference assembly** | **N50** |
| --- | --- | --- |
| *S. cerevisiae* S288C | NCBI assembly R64, RefSeq accession GCF_000146045.2 | 924431 |
| *A. thaliana* Ecotype Col-0 | NCBI assembly ASM2091176v1, GenBank accession GCA_020911765.1 (Hou *et al.*, 2022) | 26150454 |
| *C. elegans* VC2010 | ENA project accession PRJEB28388 (Yoshimura *et al.*, 2019) | 17759200 |
| *D. melanogaster* ISO-1 | NCBI RefSeq accession GCF_000001215.4 | 25286936 |
| *T. rubripes* | NCBI assembly fTakRub1.3, GenBank accession GCA_901000725.3 | 16705553 |
| *P. falciparum*  (isolate: 3D7) | NCBI assembly GCA_000002765, GenBank accession GCA_000002765.3 | 1687656 |
| *D. ananassae* strain 14024-0371.13 | NCBI RefSeq accession GCF_017639315.1 | 26427416 |

**Supplementary Table S2:** Long read sets from the human genome used to generate Badread error and QScore models for PacBio CLR and ONT. Where needed, we downsampled reads to 3 Gbp, which meets the simulator’s requirements for at least 1 Gbp of real sequence data. Read identities were calculated as described by (Wick, 2019), who used the definition of BLAST identity. The sequence data was aligned to reference GCF_009914755.1 (Nurk *et al.*, 2022), with Minimap v2.24 (Li, 2016).

| **Technology** | **Sequence data source** | **Read identities (%)**  **(mean, max, stdev)** | **Notes** |
| --- | --- | --- | --- |
| PacBio Continuous Long Reads (CLRs) | HG002 extracted DNA: <https://downloads.pacbcloud.com/public/dataset/SV-HG002-CLR/> | 89.349, 94.668, 2.678 | The read file was split into sub-files containing approximately 3 Gbp of data, using [Fastaq](https://github.com/sanger-pathogens/Fastaq/blob/master/README.md). Alignment and training were performed using the first split. |
| Oxford Nanopore reads | <https://s3.amazonaws.com/nanopore-human-wgs/chm13/nanopore/rel3/rel3.fastq.gz>  Read IDs: <https://s3.amazonaws.com/nanopore-human-wgs/chm13/nanopore/rel3/ids/ucd.ids.gz> | 91.333, 98.783, 2.899 | The read file was split into sub-files containing approximately 3 Gbp of data, using [Fastaq](https://github.com/sanger-pathogens/Fastaq/blob/master/README.md). Alignment and training were performed using the first split. |

**Supplementary Table S3:** Badread parameters used in the simulation of PacBio CLR and ONT reads. In total, using Badread we simulated 48 read sets, accounting for 6 genomes (Supplementary Table S1), 2 sequencing technologies, and 4 read length distributions per technology (Table 1). Aside from read length, these parameters were kept consistent for each technology across all simulations. All other parameters not included in this table were kept as the simulator’s defaults. Please note that the pacbio_human2019 and ont_human2019 models were generated in this study (See Supplementary Table S2).

| **Parameter** | **PacBio CLR** | **Oxford nanopore** |
| --- | --- | --- |
| **--error_model** | pacbio_human2019  (see Supplementary Table S2) | ont_human2019  (see Supplementary Table S2) |
| **--qscore_model** | pacbio_human2019  (see Supplementary Table S2) | ont_human2019  (see Supplementary Table S2) |
| **--seed** | 10 | |
| **--identity** | 89.4,94.7,2.7 | 91.3,98.8,2.9 |
| **--length** | Varies per iteration (see Table 1) | |
| **--quantity** | 30x | |
| **--junk_reads** | default | |
| **--random_reads** | default | |
| **--chimeras** | default | |
| **--glitches** | default | |

**Supplementary Table S4:** Commands and parameters used for simulating PacBio HiFi reads using PBSIM3.

| **Commands for simulating PacBio HiFi reads** |
| --- |
| **pbsim --strategy wgs --method qshmm --qshmm pbsim3/data/QSHMM-RSII.model --depth 30 --genome $reference --pass-num 10 --prefix** $directory **--seed 100 --length-mean $mean --length-sd $std**  **cat $directory/*.sam > $directory/all_reads.sam**  **samtools view -bS @ $threads $directory/all_reads.sam > $directory/all_reads.bam**  **ccs –all -j $threads $directory/all_reads.bam $directory/all_reads.fastq** |

**Supplementary Table S5:** Accession for the sequencing data used in our benchmark of real-read assemblies. To match our simulated reads, we have further downsampled these read sets to 30x coverage. Due to the low coverage of the *T. rubripes* PacBio and D. *melanogaster* ISO-1 Oxford Nanopore datasets, we combined multiple datasets and sampled reads to a coverage of approximately 30x.

|  | **Accessions** | | |
| --- | --- | --- | --- |
| **Organism** | **PacBio CLR** | **PacBio HiFi** | **Oxford Nanopore** |
| *S. cerevisiae* S288C | - | SRR18210286 | SRR17374240 |
| *A. thaliana* Ecotype Col-0 | - | SRR14728885 | SRR16149191 |
| *C. elegans* VC2010 | SRR7594465 | - | SRR7594463 |
| *D. melanogaster* ISO-1 | SRR11906525 | - | SRR6702603, SRR6821890 |
| *T. rubripes* | ERR3261643, ERR3253114, ERR3253113, ERR3253112 | - | - |
| *P. falciparum*  (isolate: 3D7) | - | SRR13050273 | ERR10113962 |
| *D. ananasse* | - | SRR11442117 | - |

**Supplementary Table S6:** Assembly commands for all assemblers. The $genome_size in the assembly commands below was set as follows: *S. cerevisiae* = 12 Mbp, *P. falciparum* = 23 Mbp, *A. thaliana* = 130 Mbp, *D. melanogaster* = 139 Mbp, *C. elegans* = 103 Mbp, *T. rubripes* = 384 Mbp, and *D. ananassae* = . The $threads parameter was set to 8 for *S. cerevisiae* and *P. falciparum*, 16 for *A. thaliana*, *C. elegans*, and *D. melanogaster*, and 20 for *T. rubripes*.

| **Assembler** | **Command** |
| --- | --- |
| Canu | canu genomeSize=$genome_size -pacbio/-nanopore/-pacbio-hifi $reads MaxThreads=$threads useGrid=false -p $prefix -d $directory |
| Flye | flye --pacbio-hifi/--pacbio-raw/--nano-raw $reads --threads $threads --out-dir $directory |
| Miniasm | minimap2 -x ava-ont/ava-pb -t $threads $reads $reads \| gzip -1 > overlap.paf.gz  miniasm -f $reads overlap.paf.gz > unitigs.gfa  minipolish -t $threads $reads unitigs.gfa > assembly.gfa |
| Raven | raven -t $threads $reads > assembly.fasta |
| Wtdbg2 | wtdbg2 -x ont/sq/ccs -g $genome_size -i $reads -t $threads -fo dbg  wtpoa-cns -t $threads -i dbg.ctg.lay.gz -fo dbg.raw.fa  minimap2 -t $threads -x map-ont/map-pb/map-hifi dbg.raw.fa $reads \| gzip > alignment1.paf.gz  racon -t $threads $reads alignment1.paf.gz dbg.raw.fa > polished1.fasta  minimap2 -t $threads -x map-ont/map-pb/map-hifi polished1.fasta $reads \| gzip > alignment2.paf.gz  racon -t $threads $reads alignment2.paf.gz polished1.fasta > assembly.fasta |
| Hifiasm | hifiasm -o $directory/assembly.asm.p_ctg.fasta --primary -t $threads $reads  awk '/^S/{print ">"$2"\n"$3}' $directory/assembly.asm \| fold > $directory/assembly.fasta |
| LJA | lja -o $directory --reads $reads -t $threads |
| MBG | MBG -i $reads -o $directory/graph.gfa -t $threads -k 1501 -w 1450 -a 1 -u 3  awk '/^S/{print ">"$2"\n"$3}' $directory/graph.gfa \| fold > $directory/assembly.fasta |

**Supplementary Table S7:** Definitions and formulas for the COMPASS metrics defined in Assemblathon 2 (Bradnam *et al.*, 2013). We define $C, V, M, P$ as the coverage, validity, multiplicity, and parsimony of an assembly, respectively. We also denote $L_{CI}$ as the total length of the coverage islands, $L_{A}$ as the total length of the alignments between the reference and the assembly, $L_{R}$ as the total length of the reference, and $L_{S}$ as the total length of the assembly (sum of the scaffold lengths).

| **COMPASS metric** | **Definition** | **Formula** |
| --- | --- | --- |
| Coverage | Coverage is a measure of the fraction of the reference genome that is present in an assembly. It is determined as the ratio between the summed length of the coverage islands and the summed length of the reference sequences. Coverage values range from 0 to 1, and a higher coverage is preferred. | $C=\frac{L_{CI}}{L_{R}}$ |
| Validity | Validity is the ratio between the summed length of the alignments and the assembled scaffolds, measuring how much of the assembly is aligned to the reference. By definition, a higher validity implies better assembly quality. However, validity values higher than 1 are encountered when the number of aligned bases is higher than the number of bases in the assembly, suggesting that some of the alignments overlap, implying that some alignments are duplicated. In this evaluation, we considered that a good validity score has a value close to 1. | $V=\frac{L_{A}}{L_{S}}$ |
| Multiplicity | Multiplicity is defined by the ratio of the summed length of the alignments and the summed length of the coverage islands. This metric gives insight on whether the assembler collapsed or replicated repeats within the genome. In the evaluation, multiplicity values close to 1 are considered better. | $M=\frac{L_{A}}{L_{CI}}$ |
| Parsimony | Parsimony is the ratio between multiplicity and validity. Low parsimony values are preferred, because this is interpreted as the assembly cost. The minimum parsimony value is 1, which means there is a one-to-one mapping from all of the assembly fragments to all of the sequences in the reference genome. | $P=\frac{M}{V}$ |

**Supplementary Table S8:** Evaluation results for the S. cerevisiae Oxford Nanopore simulated read assemblies.

| **S. cerevisiae: Oxford Nanopore reads** | | | | |  |
| --- | --- | --- | --- | --- | --- |
|  | **Iteration 1** | **Iteration 2** | **Iteration 3** | **Iteration 4** |  |
| **Canu** | 0.999 | 0.993 | 0.999 | 0.930 | **Sequence  identity** |
| **Flye** | 0.999 | 0.999 | 0.998 | 0.998 |  |
| **Miniasm** | 0.999 | 0.931 | 0.843 | 0.674 |  |
| **Raven** | 0.992 | 0.972 | 0.916 | 0.877 |  |
| **Wtdbg2** | 0.972 | 0.965 | 0.966 | 0.954 |  |
| **Canu** | 1.002 | 1.000 | 1.002 | 1.000 | **Repeat collapse** |
| **Flye** | 1.001 | 1.001 | 1.003 | 1.004 |  |
| **Miniasm** | 1.000 | 1.000 | 1.000 | 1.000 |  |
| **Raven** | 1.002 | 1.002 | 1.000 | 1.001 |  |
| **Wtdbg2** | 1.000 | 1.001 | 1.008 | 1.000 |  |
| **Canu** | 1.000 | 1.001 | 1.001 | 1.001 | **Rate of  valid sequences** |
| **Flye** | 1.001 | 1.001 | 1.002 | 1.001 |  |
| **Miniasm** | 1.000 | 1.000 | 1.000 | 1.002 |  |
| **Raven** | 1.002 | 1.000 | 1.002 | 1.001 |  |
| **Wtdbg2** | 0.992 | 0.999 | 0.988 | 0.999 |  |
| **Canu** | 0.976 | 0.975 | 0.976 | 0.940 | **Contiguity (NG50 / N50)** |
| **Flye** | 0.978 | 0.963 | 0.975 | 0.963 |  |
| **Miniasm** | 0.977 | 0.926 | 0.873 | 0.676 |  |
| **Raven** | 0.977 | 0.971 | 0.947 | 0.926 |  |
| **Wtdbg2** | 0.924 | 0.927 | 1.013 | 0.888 |  |
| **Canu** | 0.000 | 0.000 | 0.000 | 0.000 | **Misassembly count** |
| **Flye** | 0.000 | 1.000 | 1.000 | 3.000 |  |
| **Miniasm** | 0.000 | 3.000 | 0.000 | 2.000 |  |
| **Raven** | 1.000 | 0.000 | 0.000 | 1.000 |  |
| **Wtdbg2** | 1.000 | 2.000 | 3.000 | 6.000 |  |
| **Canu** | 83.500 | 84.100 | 84.000 | 79.100 | **Gene  identification (% complete  BUSCOs)** |
| **Flye** | 95.700 | 95.200 | 95.200 | 96.100 |  |
| **Miniasm** | 92.100 | 87.900 | 80.200 | 64.600 |  |
| **Raven** | 90.200 | 88.600 | 84.300 | 82.400 |  |
| **Wtdbg2** | 92.800 | 92.400 | 93.000 | 92.100 |  |
|  | | | | | |

**Supplementary Table S9:** Evaluation results for the S. cerevisiae PacBio CLR simulated read assemblies.

| **S. cerevisiae: PacBio CLR reads** | | | | | |
| --- | --- | --- | --- | --- | --- |
|  | **Iteration 1** | **Iteration 2** | **Iteration 3** | **Iteration 4** |  |
| **Canu** | 0.998 | 0.998 | 0.998 | 0.993 | **Sequence  identity** |
| **Flye** | 0.998 | 0.999 | 0.999 | 0.995 |  |
| **Miniasm** | 0.969 | 0.883 | 0.835 | 0.683 |  |
| **Raven** | 0.991 | 0.992 | 0.990 | 0.889 |  |
| **Wtdbg2** | 0.974 | 0.959 | 0.963 | 0.972 |  |
| **Canu** | 1.002 | 1.000 | 1.002 | 1.000 | **Repeat collapse** |
| **Flye** | 1.001 | 1.002 | 1.004 | 1.000 |  |
| **Miniasm** | 1.000 | 1.008 | 1.066 | 1.000 |  |
| **Raven** | 1.002 | 1.000 | 1.000 | 1.001 |  |
| **Wtdbg2** | 1.000 | 1.000 | 1.000 | 1.002 |  |
| **Canu** | 1.000 | 1.001 | 1.002 | 1.001 | **Rate of  valid sequences** |
| **Flye** | 1.001 | 1.002 | 1.001 | 1.000 |  |
| **Miniasm** | 1.000 | 0.997 | 0.999 | 1.002 |  |
| **Raven** | 1.002 | 1.000 | 1.000 | 1.001 |  |
| **Wtdbg2** | 0.995 | 0.984 | 0.996 | 0.994 |  |
| **Canu** | 0.977 | 0.976 | 0.976 | 0.975 | **Contiguity (NG50 / N50)** |
| **Flye** | 0.975 | 0.977 | 0.953 | 0.967 |  |
| **Miniasm** | 0.926 | 0.801 | 0.627 | 0.666 |  |
| **Raven** | 0.974 | 0.976 | 0.972 | 0.939 |  |
| **Wtdbg2** | 0.934 | 0.933 | 0.908 | 0.932 |  |
| **Canu** | 0.000 | 0.000 | 0.000 | 0.000 | **Misassembly count** |
| **Flye** | 0.000 | 1.000 | 1.000 | 0.000 |  |
| **Miniasm** | 0.000 | 0.000 | 2.000 | 0.000 |  |
| **Raven** | 0.000 | 0.000 | 0.000 | 0.000 |  |
| **Wtdbg2** | 0.000 | 1.000 | 1.000 | 3.000 |  |
| **Canu** | 87.500 | 87.100 | 88.900 | 88.500 | **Gene  identification (% complete  BUSCOs)** |
| **Flye** | 90.000 | 89.500 | 90.700 | 90.200 |  |
| **Miniasm** | 87.800 | 81.400 | 76.900 | 64.500 |  |
| **Raven** | 87.100 | 87.500 | 88.700 | 81.300 |  |
| **Wtdbg2** | 89.900 | 89.400 | 89.800 | 89.400 |  |

**Supplementary Table S10:** Evaluation results for the S. cerevisiae PacBio HiFi simulated read assemblies.

| **S. cerevisiae: PacBio Hifi reads** | | | | | |
| --- | --- | --- | --- | --- | --- |
|  | **Iteration 1** | **Iteration 2** | **Iteration 3** | **Iteration 4** |  |
| **Canu** | 0.995 | 0.995 | 0.993 | 0.983 | **Sequence  identity** |
| **Flye** | 0.991 | 0.997 | 0.989 | 0.985 |  |
| **Hifiasm** | 0.998 | 0.889 | 0.997 | 0.817 |  |
| **LJA** | 0.998 | 0.994 | 0.995 | 0.985 |  |
| **MBG** | 0.982 | 0.981 | 0.978 | 0.968 |  |
| **Canu** | 1.124 | 1.092 | 1.134 | 1.326 | **Repeat collapse** |
| **Flye** | 1.000 | 1.000 | 1.001 | 1.000 |  |
| **Hifiasm** | 1.000 | 1.000 | 1.000 | 1.000 |  |
| **LJA** | 1.001 | 1.000 | 1.001 | 1.000 |  |
| **MBG** | 1.063 | 1.064 | 1.063 | 1.070 |  |
| **Canu** | 1.000 | 1.000 | 1.000 | 1.000 | **Rate of  valid sequences** |
| **Flye** | 1.000 | 1.000 | 1.001 | 1.000 |  |
| **Hifiasm** | 1.000 | 1.001 | 1.000 | 1.002 |  |
| **LJA** | 1.001 | 1.000 | 1.001 | 1.000 |  |
| **MBG** | 0.999 | 0.998 | 0.999 | 0.999 |  |
| **Canu** | 0.976 | 0.975 | 0.974 | 0.993 | **Contiguity (NG50 / N50)** |
| **Flye** | 0.975 | 0.974 | 0.971 | 0.963 |  |
| **Hifiasm** | 0.976 | 0.883 | 0.974 | 0.654 |  |
| **LJA** | 0.976 | 0.972 | 0.972 | 0.956 |  |
| **MBG** | 0.103 | 0.100 | 0.107 | 0.088 |  |
| **Canu** | 0.000 | 0.000 | 0.000 | 0.000 | **Misassembly count** |
| **Flye** | 0.000 | 0.000 | 0.000 | 0.000 |  |
| **Hifiasm** | 0.000 | 2.000 | 0.000 | 3.000 |  |
| **LJA** | 0.000 | 1.000 | 0.000 | 0.000 |  |
| **MBG** | 0.000 | 0.000 | 0.000 | 0.000 |  |
| **Canu** | 99.600 | 99.600 | 99.600 | 99.600 | **Gene  identification (% complete  BUSCOs)** |
| **Flye** | 99.600 | 99.600 | 99.600 | 99.500 |  |
| **Hifiasm** | 99.600 | 90.700 | 99.600 | 84.200 |  |
| **LJA** | 99.500 | 99.500 | 99.600 | 99.500 |  |
| **MBG** | 98.400 | 98.400 | 98.200 | 98.000 |  |

**Supplementary Table S11:** Evaluation results for the P. falciparum Oxford Nanopore simulated read assemblies.

| **P. falciparum: Oxford Nanopore reads** | | | | | |
| --- | --- | --- | --- | --- | --- |
|  | **Iteration 1** | **Iteration 2** | **Iteration 3** | **Iteration 4** |  |
| **Canu** | 0.998 | 0.997 | 0.999 | 0.995 | **Sequence  identity** |
| **Flye** | 0.996 | 0.994 | 0.998 | 0.998 |  |
| **Miniasm** | 0.979 | 0.979 | 0.960 | 0.952 |  |
| **Raven** | 0.964 | 0.975 | 0.980 | 0.958 |  |
| **Wtdbg2** | 0.930 | 0.922 | 0.949 | 0.948 |  |
| **Canu** | 1.003 | 1.001 | 1.015 | 1.001 | **Repeat collapse** |
| **Flye** | 1.005 | 1.008 | 1.006 | 1.004 |  |
| **Miniasm** | 1.001 | 1.001 | 1.000 | 1.001 |  |
| **Raven** | 1.003 | 1.005 | 1.007 | 1.004 |  |
| **Wtdbg2** | 1.002 | 1.004 | 1.003 | 1.000 |  |
| **Canu** | 0.998 | 0.996 | 1.000 | 1.002 | **Rate of  valid sequences** |
| **Flye** | 1.001 | 1.002 | 1.001 | 1.001 |  |
| **Miniasm** | 1.001 | 1.001 | 1.001 | 1.001 |  |
| **Raven** | 0.998 | 0.996 | 0.996 | 0.996 |  |
| **Wtdbg2** | 0.997 | 0.991 | 0.994 | 0.989 |  |
| **Canu** | 1.023 | 1.057 | 1.160 | 1.153 | **Contiguity (NG50 / N50)** |
| **Flye** | 1.082 | 1.015 | 1.124 | 1.143 |  |
| **Miniasm** | 1.129 | 1.134 | 1.114 | 1.116 |  |
| **Raven** | 1.110 | 1.139 | 1.152 | 1.147 |  |
| **Wtdbg2** | 1.054 | 1.161 | 0.957 | 1.229 |  |
| **Canu** | 0.000 | 0.000 | 2.000 | 0.000 | **Misassembly count** |
| **Flye** | 4.000 | 3.000 | 3.000 | 4.000 |  |
| **Miniasm** | 0.000 | 0.000 | 0.000 | 1.000 |  |
| **Raven** | 8.000 | 13.000 | 14.000 | 7.000 |  |
| **Wtdbg2** | 6.000 | 6.000 | 2.000 | 2.000 |  |
| **Canu** | 87.600 | 88.600 | 89.400 | 89.200 | **Gene  identification (% complete  BUSCOs)** |
| **Flye** | 97.800 | 97.700 | 98.100 | 98.000 |  |
| **Miniasm** | 92.500 | 92.400 | 90.400 | 90.700 |  |
| **Raven** | 91.600 | 91.400 | 90.900 | 89.900 |  |
| **Wtdbg2** | 92.900 | 92.300 | 93.700 | 93.400 |  |

**Supplementary Table S12:** Evaluation results for the P. falciparum PacBio CLR simulated read assemblies.

| **P. falciparum: PacBio CLR reads** | | | | | |
| --- | --- | --- | --- | --- | --- |
|  | **Iteration 1** | **Iteration 2** | **Iteration 3** | **Iteration 4** |  |
| **Canu** | 0.956 | 0.959 | 0.975 | 0.974 | **Sequence  identity** |
| **Flye** | 0.999 | 0.994 | 0.996 | 0.996 |  |
| **Miniasm** | 0.973 | 0.937 | 0.934 | 0.836 |  |
| **Raven** | 0.970 | 0.975 | 0.980 | 0.983 |  |
| **Wtdbg2** | 0.950 | 0.954 | 0.961 | 0.960 |  |
| **Canu** | 1.002 | 1.003 | 1.001 | 1.001 | **Repeat collapse** |
| **Flye** | 1.006 | 1.007 | 1.004 | 1.005 |  |
| **Miniasm** | 1.009 | 1.012 | 1.011 | 1.012 |  |
| **Raven** | 1.002 | 1.004 | 1.006 | 1.005 |  |
| **Wtdbg2** | 1.001 | 1.000 | 1.000 | 1.000 |  |
| **Canu** | 1.000 | 1.002 | 1.004 | 1.004 | **Rate of  valid sequences** |
| **Flye** | 1.000 | 0.999 | 1.003 | 1.003 |  |
| **Miniasm** | 0.997 | 1.000 | 1.000 | 0.999 |  |
| **Raven** | 0.996 | 0.992 | 0.993 | 0.973 |  |
| **Wtdbg2** | 0.997 | 0.993 | 0.992 | 0.990 |  |
| **Canu** | 0.178 | 0.253 | 0.475 | 0.538 | **Contiguity (NG50 / N50)** |
| **Flye** | 1.136 | 1.133 | 1.122 | 1.122 |  |
| **Miniasm** | 1.061 | 0.842 | 0.890 | 0.836 |  |
| **Raven** | 1.124 | 1.143 | 1.157 | 1.159 |  |
| **Wtdbg2** | 0.878 | 0.980 | 1.018 | 0.998 |  |
| **Canu** | 0.000 | 0.000 | 0.000 | 0.000 | **Misassembly count** |
| **Flye** | 2.000 | 3.000 | 2.000 | 2.000 |  |
| **Miniasm** | 0.000 | 0.000 | 0.000 | 1.000 |  |
| **Raven** | 4.000 | 9.000 | 7.000 | 4.000 |  |
| **Wtdbg2** | 5.000 | 3.000 | 1.000 | 0.000 |  |
| **Canu** | 75.400 | 77.200 | 79.600 | 79.000 | **Gene  identification (% complete  BUSCOs)** |
| **Flye** | 88.900 | 88.100 | 88.100 | 88.400 |  |
| **Miniasm** | 86.800 | 84.900 | 83.300 | 76.700 |  |
| **Raven** | 85.300 | 85.200 | 85.900 | 85.300 |  |
| **Wtdbg2** | 88.100 | 87.500 | 87.800 | 87.400 |  |

**Supplementary Table S13:** Evaluation results for the P. falciparum PacBio HiFi simulated read assemblies.

| **P. falciparum: PacBio HiFi reads** | | | | | |
| --- | --- | --- | --- | --- | --- |
|  | **Iteration 1** | **Iteration 2** | **Iteration 3** | **Iteration 4** |  |
| **Canu** | 0.997 | 0.997 | 0.994 | 0.991 | **Sequence  identity** |
| **Flye** | 0.932 | 0.876 | 0.721 | 0.189 |  |
| **Hifiasm** | 0.998 | 0.999 | 0.996 | 0.993 |  |
| **LJA** | 0.996 | 0.997 | 0.994 | 0.984 |  |
| **MBG** | 0.900 | 0.907 | 0.914 | 0.903 |  |
| **Canu** | 1.145 | 1.013 | 1.001 | 1.001 | **Repeat collapse** |
| **Flye** | 1.007 | 1.007 | 1.006 | 1.013 |  |
| **Hifiasm** | 1.002 | 1.001 | 1.001 | 1.001 |  |
| **LJA** | 1.003 | 1.001 | 1.002 | 1.001 |  |
| **MBG** | 1.693 | 1.722 | 1.754 | 1.802 |  |
| **Canu** | 1.001 | 1.000 | 1.000 | 1.000 | **Rate of  valid sequences** |
| **Flye** | 1.000 | 1.000 | 1.002 | 1.009 |  |
| **Hifiasm** | 1.000 | 1.000 | 1.000 | 1.000 |  |
| **LJA** | 1.000 | 1.000 | 1.001 | 1.000 |  |
| **MBG** | 0.999 | 1.000 | 1.000 | 1.000 |  |
| **Canu** | 1.163 | 1.162 | 1.158 | 1.151 | **Contiguity (NG50 / N50)** |
| **Flye** | 0.280 | 0.210 | 0.163 | 0.036 |  |
| **Hifiasm** | 1.164 | 1.165 | 1.162 | 1.155 |  |
| **LJA** | 1.162 | 1.165 | 1.159 | 1.141 |  |
| **MBG** | 0.005 | 0.005 | 0.005 | 0.005 |  |
| **Canu** | 1.000 | 0.000 | 0.000 | 0.000 | **Misassembly count** |
| **Flye** | 2.000 | 2.000 | 2.000 | 11.000 |  |
| **Hifiasm** | 0.000 | 0.000 | 0.000 | 0.000 |  |
| **LJA** | 0.000 | 0.000 | 0.000 | 0.000 |  |
| **MBG** | 0.000 | 0.000 | 0.000 | 0.000 |  |
| **Canu** | 95.400 | 95.600 | 95.500 | 95.100 | **Gene  identification (% complete  BUSCOs)** |
| **Flye** | 86.200 | 79.300 | 62.700 | 11.700 |  |
| **Hifiasm** | 95.300 | 94.900 | 94.800 | 94.200 |  |
| **LJA** | 95.200 | 95.200 | 95.400 | 95.300 |  |
| **MBG** | 69.100 | 69.800 | 71.300 | 70.400 |  |

**Supplementary Table S14:** Evaluation results for the C. elegans Oxford Nanopore simulated read assemblies.

| **C. elegans: Oxford Nanopore reads** | | | | | |
| --- | --- | --- | --- | --- | --- |
|  | **Iteration 1** | **Iteration 2** | **Iteration 3** | **Iteration 4** |  |
| **Canu** | 1.000 | 1.000 | 0.999 | 0.998 | **Sequence  identity** |
| **Flye** | 0.998 | 0.998 | 0.998 | 0.998 |  |
| **Miniasm** | 0.996 | 0.991 | 0.989 | 0.990 |  |
| **Raven** | 0.994 | 0.997 | 0.996 | 0.996 |  |
| **Wtdbg2** | 0.974 | 0.984 | 0.955 | 0.951 |  |
| **Canu** | 1.004 | 1.004 | 1.003 | 1.002 | **Repeat collapse** |
| **Flye** | 1.005 | 1.007 | 1.005 | 1.010 |  |
| **Miniasm** | 1.002 | 1.002 | 1.003 | 1.002 |  |
| **Raven** | 1.004 | 1.003 | 1.007 | 1.003 |  |
| **Wtdbg2** | 1.003 | 1.002 | 1.002 | 1.005 |  |
| **Canu** | 1.002 | 1.001 | 0.997 | 1.004 | **Rate of  valid sequences** |
| **Flye** | 1.005 | 1.005 | 1.003 | 1.004 |  |
| **Miniasm** | 1.001 | 1.001 | 1.002 | 1.002 |  |
| **Raven** | 1.003 | 1.002 | 1.003 | 0.994 |  |
| **Wtdbg2** | 0.997 | 0.995 | 0.996 | 0.995 |  |
| **Canu** | 0.966 | 0.964 | 0.935 | 0.922 | **Contiguity (NG50 / N50)** |
| **Flye** | 0.894 | 0.958 | 0.865 | 0.985 |  |
| **Miniasm** | 0.517 | 0.810 | 0.956 | 0.959 |  |
| **Raven** | 0.966 | 0.970 | 0.836 | 0.888 |  |
| **Wtdbg2** | 0.502 | 0.373 | 0.631 | 0.707 |  |
| **Canu** | 3.000 | 6.000 | 5.000 | 8.000 | **Misassembly count** |
| **Flye** | 37.000 | 36.000 | 29.000 | 49.000 |  |
| **Miniasm** | 22.000 | 33.000 | 53.000 | 34.000 |  |
| **Raven** | 56.000 | 43.000 | 36.000 | 55.000 |  |
| **Wtdbg2** | 38.000 | 27.000 | 39.000 | 72.000 |  |
| **Canu** | 95.700 | 96.000 | 95.900 | 95.000 | **Gene  identification (% complete  BUSCOs)** |
| **Flye** | 97.400 | 97.500 | 97.800 | 97.500 |  |
| **Miniasm** | 97.900 | 97.300 | 97.400 | 96.500 |  |
| **Raven** | 97.400 | 97.500 | 97.000 | 97.200 |  |
| **Wtdbg2** | 97.200 | 97.600 | 95.700 | 93.900 |  |

**Supplementary Table S15:** Evaluation results for the C. elegans PacBio CLR simulated read assemblies.

| **C. elegans: PacBio CLR reads** | | | | | |
| --- | --- | --- | --- | --- | --- |
|  | **Iteration 1** | **Iteration 2** | **Iteration 3** | **Iteration 4** |  |
| **Canu** | 1.000 | 1.000 | 1.000 | 0.999 | **Sequence  identity** |
| **Flye** | 0.998 | 0.998 | 0.998 | 0.998 |  |
| **Miniasm** | 0.993 | 0.993 | 0.988 | 0.975 |  |
| **Raven** | 0.994 | 0.995 | 0.997 | 0.997 |  |
| **Wtdbg2** | 0.988 | 0.992 | 0.989 | 0.985 |  |
| **Canu** | 1.003 | 1.001 | 1.003 | 1.003 | **Repeat collapse** |
| **Flye** | 1.005 | 1.005 | 1.005 | 1.009 |  |
| **Miniasm** | 1.005 | 1.008 | 1.016 | 1.009 |  |
| **Raven** | 1.004 | 1.003 | 1.004 | 1.003 |  |
| **Wtdbg2** | 1.003 | 1.003 | 1.003 | 1.004 |  |
| **Canu** | 1.001 | 0.999 | 0.999 | 1.001 | **Rate of  valid sequences** |
| **Flye** | 0.998 | 0.997 | 0.998 | 1.002 |  |
| **Miniasm** | 1.000 | 1.001 | 1.000 | 1.000 |  |
| **Raven** | 1.001 | 1.001 | 1.002 | 1.001 |  |
| **Wtdbg2** | 0.997 | 0.996 | 0.995 | 0.995 |  |
| **Canu** | 0.846 | 0.921 | 0.938 | 0.953 | **Contiguity (NG50 / N50)** |
| **Flye** | 0.710 | 0.782 | 0.818 | 0.918 |  |
| **Miniasm** | 0.484 | 0.547 | 0.392 | 0.590 |  |
| **Raven** | 0.917 | 0.972 | 0.974 | 0.975 |  |
| **Wtdbg2** | 0.540 | 0.645 | 0.809 | 0.954 |  |
| **Canu** | 10.000 | 10.000 | 4.000 | 1.000 | **Misassembly count** |
| **Flye** | 21.000 | 33.000 | 40.000 | 30.000 |  |
| **Miniasm** | 7.000 | 6.000 | 3.000 | 10.000 |  |
| **Raven** | 53.000 | 40.000 | 39.000 | 55.000 |  |
| **Wtdbg2** | 22.000 | 31.000 | 34.000 | 50.000 |  |
| **Canu** | 98.500 | 98.400 | 98.500 | 98.100 | **Gene  identification (% complete  BUSCOs)** |
| **Flye** | 98.700 | 98.700 | 98.700 | 98.700 |  |
| **Miniasm** | 97.700 | 98.000 | 97.900 | 95.600 |  |
| **Raven** | 97.900 | 98.300 | 98.200 | 98.100 |  |
| **Wtdbg2** | 98.200 | 98.300 | 98.100 | 97.100 |  |

**Supplementary Table S16:** Evaluation results for the A. thaliana Oxford Nanopore simulated read assemblies.

| **A. thaliana: Oxford Nanopore reads** | | | | | |
| --- | --- | --- | --- | --- | --- |
|  | **Iteration 1** | **Iteration 2** | **Iteration 3** | **Iteration 4** |  |
| **Canu** | 0.929 | 0.934 | 0.937 |  | **Sequence  identity** |
| **Flye** | 0.913 | 0.920 | 0.925 | 0.936 |  |
| **Miniasm** | 0.902 | 0.906 | 0.909 | 0.914 |  |
| **Raven** | 0.902 | 0.909 | 0.910 | 0.915 |  |
| **Wtdbg2** | 0.879 | 0.885 | 0.859 | 0.871 |  |
| **Canu** | 1.059 | 1.055 | 1.108 | 0.000 | **Repeat collapse** |
| **Flye** | 1.004 | 1.003 | 1.004 | 1.005 |  |
| **Miniasm** | 1.012 | 1.013 | 1.012 | 1.007 |  |
| **Raven** | 1.025 | 1.035 | 1.036 | 1.043 |  |
| **Wtdbg2** | 1.028 | 1.068 | 1.086 | 1.105 |  |
| **Canu** | 1.020 | 1.017 | 1.028 |  | **Rate of  valid sequences** |
| **Flye** | 1.002 | 1.002 | 1.003 | 1.002 |  |
| **Miniasm** | 1.006 | 1.007 | 1.006 | 1.002 |  |
| **Raven** | 1.012 | 1.016 | 1.014 | 1.013 |  |
| **Wtdbg2** | 1.007 | 1.008 | 1.008 | 1.004 |  |
| **Canu** | 0.472 | 0.485 | 0.630 |  | **Contiguity (NG50 / N50)** |
| **Flye** | 0.388 | 0.460 | 0.468 | 0.482 |  |
| **Miniasm** | 0.412 | 0.474 | 0.442 | 0.456 |  |
| **Raven** | 0.442 | 0.437 | 0.462 | 0.505 |  |
| **Wtdbg2** | 0.206 | 0.215 | 0.156 | 0.245 |  |
| **Canu** | 461.000 | 423.000 | 871.000 |  | **Misassembly count** |
| **Flye** | 30.000 | 9.000 | 7.000 | 9.000 |  |
| **Miniasm** | 159.000 | 155.000 | 111.000 | 6.000 |  |
| **Raven** | 312.000 | 459.000 | 467.000 | 596.000 |  |
| **Wtdbg2** | 405.000 | 388.000 | 424.000 | 335.000 |  |
| **Canu** | 96.700 | 96.600 | 96.500 |  | **Gene  identification (% complete  BUSCOs)** |
| **Flye** | 98.400 | 98.600 | 98.500 | 98.500 |  |
| **Miniasm** | 98.000 | 98.000 | 98.000 | 98.000 |  |
| **Raven** | 97.700 | 97.800 | 97.500 | 97.500 |  |
| **Wtdbg2** | 95.100 | 95.000 | 92.400 | 93.100 |  |

**Supplementary Table S17:** Evaluation results for the A. thaliana PacBio CLR simulated read assemblies.

| **A. thaliana: PacBio CLR reads** | | | | | |
| --- | --- | --- | --- | --- | --- |
|  | **Iteration 1** | **Iteration 2** | **Iteration 3** | **Iteration 4** |  |
| **Canu** | 0.932 | 0.931 | 0.936 | 0.935 | **Sequence  identity** |
| **Flye** | 0.904 | 0.909 | 0.910 | 0.919 |  |
| **Miniasm** | 0.901 | 0.898 | 0.896 | 0.891 |  |
| **Raven** | 0.898 | 0.902 | 0.904 | 0.906 |  |
| **Wtdbg2** | 0.884 | 0.885 | 0.878 | 0.889 |  |
| **Canu** | 1.049 | 1.030 | 1.047 | 1.047 | **Repeat collapse** |
| **Flye** | 1.003 | 1.003 | 1.007 | 1.006 |  |
| **Miniasm** | 1.010 | 1.008 | 1.011 | 1.014 |  |
| **Raven** | 1.016 | 1.019 | 1.028 | 1.033 |  |
| **Wtdbg2** | 1.019 | 1.018 | 1.026 | 1.052 |  |
| **Canu** | 1.019 | 1.012 | 1.017 | 1.015 | **Rate of  valid sequences** |
| **Flye** | 0.998 | 0.999 | 1.002 | 1.004 |  |
| **Miniasm** | 1.004 | 1.003 | 1.002 | 1.001 |  |
| **Raven** | 1.008 | 1.010 | 1.011 | 1.013 |  |
| **Wtdbg2** | 1.005 | 1.001 | 1.002 | 0.999 |  |
| **Canu** | 0.449 | 0.473 | 0.480 | 0.495 | **Contiguity (NG50 / N50)** |
| **Flye** | 0.321 | 0.424 | 0.461 | 0.465 |  |
| **Miniasm** | 0.280 | 0.383 | 0.387 | 0.453 |  |
| **Raven** | 0.482 | 0.535 | 0.464 | 0.593 |  |
| **Wtdbg2** | 0.243 | 0.194 | 0.209 | 0.360 |  |
| **Canu** | 257.000 | 225.000 | 406.000 | 397.000 | **Misassembly count** |
| **Flye** | 19.000 | 23.000 | 19.000 | 50.000 |  |
| **Miniasm** | 94.000 | 82.000 | 57.000 | 64.000 |  |
| **Raven** | 215.000 | 232.000 | 287.000 | 374.000 |  |
| **Wtdbg2** | 285.000 | 259.000 | 237.000 | 231.000 |  |
| **Canu** | 98.100 | 97.800 | 98.100 | 98.000 | **Gene  identification (% complete  BUSCOs)** |
| **Flye** | 98.500 | 98.800 | 98.700 | 98.600 |  |
| **Miniasm** | 97.400 | 97.200 | 97.600 | 96.700 |  |
| **Raven** | 97.400 | 97.300 | 97.400 | 97.100 |  |
| **Wtdbg2** | 97.400 | 96.400 | 96.200 | 97.100 |  |

**Supplementary Table S18:** Evaluation results for the A. thaliana PacBio Hifi simulated read assemblies.

| **A. thaliana: PacBio HiFi reads** | | | | | |
| --- | --- | --- | --- | --- | --- |
|  | **Iteration 1** | **Iteration 2** | **Iteration 3** | **Iteration 4** |  |
| **Canu** | 0.998 | 0.997 | 0.995 | 0.722 | **Sequence  identity** |
| **Flye** | 0.993 | 0.994 | 0.988 | 0.695 |  |
| **Hifiasm** | 0.996 | 0.996 | 0.996 | 0.827 |  |
| **LJA** | 0.998 | 0.998 | 0.997 | 0.704 |  |
| **MBG** | 0.990 | 0.989 | 0.987 | 0.730 |  |
| **Canu** | 1.131 | 1.144 | 1.204 | 1.001 | **Repeat collapse** |
| **Flye** | 1.002 | 1.003 | 1.002 | 1.003 |  |
| **Hifiasm** | 1.001 | 1.001 | 1.002 | 1.002 |  |
| **LJA** | 1.000 | 1.001 | 1.001 | 1.001 |  |
| **MBG** | 1.101 | 1.105 | 1.104 | 1.098 |  |
| **Canu** | 0.998 | 0.999 | 1.001 | 1.001 | **Rate of  valid sequences** |
| **Flye** | 1.002 | 1.002 | 1.002 | 1.001 |  |
| **Hifiasm** | 1.001 | 1.001 | 1.001 | 1.001 |  |
| **LJA** | 1.000 | 1.001 | 1.001 | 1.000 |  |
| **MBG** | 0.999 | 0.999 | 0.999 | 0.999 |  |
| **Canu** | 0.720 | 0.644 | 0.603 | 0.311 | **Contiguity (NG50 / N50)** |
| **Flye** | 0.396 | 0.430 | 0.412 | 0.309 |  |
| **Hifiasm** | 0.880 | 0.784 | 0.614 | 0.332 |  |
| **LJA** | 0.884 | 0.764 | 0.613 | 0.323 |  |
| **MBG** | 0.003 | 0.003 | 0.003 | 0.002 |  |
| **Canu** | 0.000 | 1.000 | 1.000 | 0.000 | **Misassembly count** |
| **Flye** | 3.000 | 4.000 | 7.000 | 3.000 |  |
| **Hifiasm** | 3.000 | 4.000 | 2.000 | 4.000 |  |
| **LJA** | 0.000 | 1.000 | 3.000 | 4.000 |  |
| **MBG** | 0.000 | 0.000 | 0.000 | 1.000 |  |
| **Canu** | 99.200 | 99.200 | 99.300 | 77.200 | **Gene  identification (% complete  BUSCOs)** |
| **Flye** | 99.200 | 99.200 | 99.200 | 74.600 |  |
| **Hifiasm** | 99.300 | 99.200 | 99.200 | 86.300 |  |
| **LJA** | 99.200 | 99.200 | 99.200 | 74.600 |  |
| **MBG** | 97.000 | 97.100 | 96.900 | 76.100 |  |

**Supplementary Table S19:** Evaluation results for the D. melanogaster Oxford Nanopore simulated read assemblies.

| **D. melanogaster: Oxford Nanopore reads** | | | | | |
| --- | --- | --- | --- | --- | --- |
|  | **Iteration 1** | **Iteration 2** | **Iteration 3** | **Iteration 4** |  |
| **Canu** | 0.980 | 0.980 | 0.981 | 0.982 | **Sequence  identity** |
| **Flye** | 0.961 | 0.965 | 0.966 | 0.964 |  |
| **Miniasm** | 0.938 | 0.935 | 0.922 | 0.868 |  |
| **Raven** | 0.948 | 0.953 | 0.951 | 0.949 |  |
| **Wtdbg2** | 0.924 | 0.930 | 0.914 | 0.921 |  |
| **Canu** | 1.010 | 1.012 | 1.014 | 1.010 | **Repeat collapse** |
| **Flye** | 1.016 | 1.011 | 1.013 | 1.012 |  |
| **Miniasm** | 1.012 | 1.010 | 1.013 | 1.031 |  |
| **Raven** | 1.011 | 1.009 | 1.010 | 1.010 |  |
| **Wtdbg2** | 1.032 | 1.119 | 1.148 | 1.189 |  |
| **Canu** | 1.005 | 1.003 | 0.998 | 0.999 | **Rate of  valid sequences** |
| **Flye** | 1.007 | 1.006 | 1.007 | 1.008 |  |
| **Miniasm** | 1.002 | 1.003 | 1.001 | 1.000 |  |
| **Raven** | 1.005 | 1.002 | 1.004 | 0.999 |  |
| **Wtdbg2** | 0.998 | 0.996 | 0.992 | 0.989 |  |
| **Canu** | 0.924 | 0.932 | 0.962 | 0.941 | **Contiguity (NG50 / N50)** |
| **Flye** | 0.837 | 0.780 | 0.734 | 0.762 |  |
| **Miniasm** | 0.377 | 0.562 | 0.510 | 0.326 |  |
| **Raven** | 0.892 | 0.900 | 0.936 | 0.987 |  |
| **Wtdbg2** | 0.465 | 0.485 | 0.500 | 0.274 |  |
| **Canu** | 148.000 | 131.000 | 145.000 | 168.000 | **Misassembly count** |
| **Flye** | 53.000 | 29.000 | 43.000 | 49.000 |  |
| **Miniasm** | 52.000 | 41.000 | 27.000 | 34.000 |  |
| **Raven** | 173.000 | 182.000 | 178.000 | 230.000 |  |
| **Wtdbg2** | 238.000 | 501.000 | 614.000 | 681.000 |  |
| **Canu** | 95.000 | 95.000 | 94.300 | 93.500 | **Gene  identification (% complete  BUSCOs)** |
| **Flye** | 97.800 | 98.000 | 97.900 | 97.600 |  |
| **Miniasm** | 97.600 | 97.800 | 97.500 | 94.700 |  |
| **Raven** | 97.200 | 97.300 | 97.300 | 97.600 |  |
| **Wtdbg2** | 96.700 | 94.800 | 93.200 | 92.500 |  |

**Supplementary Table S20**: Evaluation results for the D. melanogaster PacBio CLR simulated read assemblies.

| **D. melanogaster: PacBio CLR reads** | | | | | |
| --- | --- | --- | --- | --- | --- |
|  | **Iteration 1** | **Iteration 2** | **Iteration 3** | **Iteration 4** |  |
| **Canu** | 0.977 | 0.977 | 0.973 | 0.974 | **Sequence  identity** |
| **Flye** | 0.959 | 0.963 | 0.964 | 0.966 |  |
| **Miniasm** | 0.937 | 0.931 | 0.903 | 0.841 |  |
| **Raven** | 0.952 | 0.954 | 0.953 | 0.954 |  |
| **Wtdbg2** | 0.920 | 0.937 | 0.930 | 0.921 |  |
| **Canu** | 1.009 | 1.008 | 1.008 | 1.007 | **Repeat collapse** |
| **Flye** | 1.009 | 1.008 | 1.010 | 1.013 |  |
| **Miniasm** | 1.019 | 1.037 | 1.033 | 1.045 |  |
| **Raven** | 1.008 | 1.007 | 1.005 | 1.008 |  |
| **Wtdbg2** | 1.008 | 1.019 | 1.054 | 1.129 |  |
| **Canu** | 1.001 | 1.002 | 1.002 | 1.004 | **Rate of  valid sequences** |
| **Flye** | 0.998 | 0.997 | 1.002 | 1.004 |  |
| **Miniasm** | 0.999 | 0.998 | 0.997 | 0.999 |  |
| **Raven** | 1.004 | 1.004 | 1.003 | 0.998 |  |
| **Wtdbg2** | 0.999 | 0.999 | 0.992 | 0.984 |  |
| **Canu** | 0.560 | 0.857 | 0.859 | 0.869 | **Contiguity (NG50 / N50)** |
| **Flye** | 0.791 | 0.738 | 0.811 | 0.771 |  |
| **Miniasm** | 0.167 | 0.151 | 0.203 | 0.205 |  |
| **Raven** | 0.714 | 0.867 | 0.946 | 0.973 |  |
| **Wtdbg2** | 0.660 | 0.619 | 0.697 | 0.623 |  |
| **Canu** | 98.000 | 88.000 | 104.000 | 111.000 | **Misassembly count** |
| **Flye** | 36.000 | 18.000 | 36.000 | 40.000 |  |
| **Miniasm** | 39.000 | 42.000 | 41.000 | 54.000 |  |
| **Raven** | 69.000 | 55.000 | 33.000 | 53.000 |  |
| **Wtdbg2** | 143.000 | 134.000 | 206.000 | 375.000 |  |
| **Canu** | 97.600 | 97.300 | 98.000 | 97.300 | **Gene  identification (% complete  BUSCOs)** |
| **Flye** | 98.400 | 98.100 | 98.500 | 98.400 |  |
| **Miniasm** | 97.300 | 97.000 | 96.300 | 91.200 |  |
| **Raven** | 97.200 | 96.500 | 97.300 | 97.200 |  |
| **Wtdbg2** | 96.900 | 97.000 | 96.100 | 94.000 |  |

**Supplementary Table S21:** Evaluation results for the D. ananassae PacBio HiFi simulated read assemblies.

| **D. ananassae: PacBio HiFi reads** | | | | | |
| --- | --- | --- | --- | --- | --- |
|  | **Iteration 1** | **Iteration 2** | **Iteration 3** | **Iteration 4** |  |
| **Canu** | 0.998 | 0.997 | 0.997 | 0.992 | **Sequence  identity** |
| **Flye** | 0.980 | 0.981 | 0.983 | 0.982 |  |
| **Hifiasm** | 0.961 | 0.968 | 0.960 | 0.954 |  |
| **LJA** | 0.997 | 0.996 | 0.996 | 0.991 |  |
| **MBG** | 0.971 | 0.971 | 0.970 | 0.966 |  |
| **Canu** | 1.139 | 1.165 | 1.204 | 1.017 | **Repeat collapse** |
| **Flye** | 1.006 | 1.005 | 1.004 | 1.004 |  |
| **Hifiasm** | 1.001 | 1.001 | 1.003 | 1.002 |  |
| **LJA** | 1.002 | 1.002 | 1.002 | 1.002 |  |
| **MBG** | 1.087 | 1.089 | 1.093 | 1.097 |  |
| **Canu** | 1.003 | 1.003 | 1.003 | 1.003 | **Rate of  valid sequences** |
| **Flye** | 1.005 | 1.004 | 1.004 | 1.004 |  |
| **Hifiasm** | 1.003 | 1.003 | 1.003 | 1.003 |  |
| **LJA** | 1.003 | 1.002 | 1.004 | 1.003 |  |
| **MBG** | 0.997 | 0.996 | 0.997 | 0.996 |  |
| **Canu** | 0.703 | 0.613 | 0.684 | 0.567 | **Contiguity (NG50 / N50)** |
| **Flye** | 0.471 | 0.575 | 0.601 | 0.532 |  |
| **Hifiasm** | 0.708 | 0.662 | 0.689 | 0.682 |  |
| **LJA** | 0.694 | 0.654 | 0.578 | 0.643 |  |
| **MBG** | 0.004 | 0.004 | 0.004 | 0.004 |  |
| **Canu** | 24.000 | 19.000 | 16.000 | 16.000 | **Misassembly count** |
| **Flye** | 51.000 | 36.000 | 29.000 | 36.000 |  |
| **Hifiasm** | 31.000 | 32.000 | 24.000 | 25.000 |  |
| **LJA** | 17.000 | 22.000 | 21.000 | 15.000 |  |
| **MBG** | 3.000 | 2.000 | 3.000 | 1.000 |  |
| **Canu** | 99.100 | 99.100 | 99.000 | 99.000 | **Gene  identification (% complete  BUSCOs)** |
| **Flye** | 99.100 | 99.100 | 99.100 | 99.100 |  |
| **Hifiasm** | 99.100 | 99.100 | 99.000 | 99.100 |  |
| **LJA** | 99.000 | 99.000 | 99.000 | 98.900 |  |
| **MBG** | 97.700 | 97.600 | 97.400 | 97.500 |  |

**Supplementary Table S22:** Evaluation results for the T. rubripes Oxford Nanopore simulated read assemblies.

| **T. rubripes: ONT reads** | | | | | |
| --- | --- | --- | --- | --- | --- |
|  | **Iteration 1** | **Iteration 2** | **Iteration 3** | **Iteration 4** |  |
| **Canu** | 0.984 | 0.985 | 0.983 |  | **Sequence  identity** |
| **Flye** | 0.957 | 0.965 | 0.967 | 0.970 |  |
| **Miniasm** | 0.956 | 0.948 | 0.944 | 0.914 |  |
| **Raven** | 0.960 | 0.965 | 0.969 | 0.967 |  |
| **Wtdbg2** | 0.891 | 0.892 | 0.900 | 0.915 |  |
| **Canu** | 1.019 | 1.010 | 1.013 |  | **Repeat collapse** |
| **Flye** | 1.014 | 1.013 | 1.013 | 1.018 |  |
| **Miniasm** | 1.016 | 1.013 | 1.007 | 1.005 |  |
| **Raven** | 1.018 | 1.017 | 1.018 | 1.017 |  |
| **Wtdbg2** | 1.012 | 1.025 | 1.031 | 1.043 |  |
| **Canu** | 1.004 | 1.003 | 1.006 |  | **Rate of  valid sequences** |
| **Flye** | 1.010 | 1.010 | 1.008 | 1.009 |  |
| **Miniasm** | 1.008 | 1.005 | 1.003 | 1.004 |  |
| **Raven** | 1.010 | 1.007 | 1.006 | 1.002 |  |
| **Wtdbg2** | 1.011 | 1.009 | 1.008 | 1.002 |  |
| **Canu** | 0.648 | 0.864 | 0.923 |  | **Contiguity (NG50 / N50)** |
| **Flye** | 0.377 | 0.509 | 0.738 | 0.829 |  |
| **Miniasm** | 0.462 | 0.607 | 0.699 | 0.759 |  |
| **Raven** | 0.555 | 0.715 | 0.859 | 0.864 |  |
| **Wtdbg2** | 0.437 | 0.395 | 0.393 | 0.401 |  |
| **Canu** | 48.000 | 29.000 | 41.000 |  | **Misassembly count** |
| **Flye** | 123.000 | 99.000 | 102.000 | 101.000 |  |
| **Miniasm** | 102.000 | 38.000 | 30.000 | 44.000 |  |
| **Raven** | 339.000 | 248.000 | 209.000 | 164.000 |  |
| **Wtdbg2** | 412.000 | 377.000 | 382.000 | 381.000 |  |
| **Canu** | 93.800 | 93.600 | 93.000 |  | **Gene  identification (% complete  BUSCOs)** |
| **Flye** | 95.900 | 95.500 | 95.600 | 95.500 |  |
| **Miniasm** | 95.800 | 95.000 | 94.500 | 92.400 |  |
| **Raven** | 95.500 | 95.300 | 95.100 | 95.500 |  |
| **Wtdbg2** | 92.300 | 91.700 | 92.600 | 92.900 |  |

**Supplementary Table S23:** Evaluation results for the T. rubripes PacBio CLR simulated read assemblies.

| **T. rubripes: PacBio CLR reads** | | | | | |
| --- | --- | --- | --- | --- | --- |
|  | **Iteration 1** | **Iteration 2** | **Iteration 3** | **Iteration 4** |  |
| **Canu** | 0.980 | 0.979 | 0.979 | 0.971 | **Sequence  identity** |
| **Flye** | 0.957 | 0.959 | 0.962 | 0.968 |  |
| **Miniasm** | 0.959 | 0.957 | 0.955 | 0.931 |  |
| **Raven** | 0.956 | 0.963 | 0.966 | 0.962 |  |
| **Wtdbg2** | 0.905 | 0.892 | 0.929 | 0.917 |  |
| **Canu** | 1.021 | 1.013 | 1.010 | 1.008 | **Repeat collapse** |
| **Flye** | 1.013 | 1.009 | 1.010 | 1.011 |  |
| **Miniasm** | 1.023 | 1.024 | 1.018 | 1.021 |  |
| **Raven** | 1.012 | 1.012 | 1.011 | 1.012 |  |
| **Wtdbg2** | 1.006 | 1.011 | 1.015 | 1.028 |  |
| **Canu** | 1.007 | 1.005 | 1.003 | 1.003 | **Rate of  valid sequences** |
| **Flye** | 1.006 | 1.006 | 1.006 | 1.007 |  |
| **Miniasm** | 1.006 | 1.006 | 1.003 | 1.002 |  |
| **Raven** | 1.010 | 1.007 | 1.006 | 1.004 |  |
| **Wtdbg2** | 1.008 | 1.009 | 1.009 | 1.001 |  |
| **Canu** | 0.449 | 0.642 | 0.773 | 0.756 | **Contiguity (NG50 / N50)** |
| **Flye** | 0.346 | 0.420 | 0.446 | 0.687 |  |
| **Miniasm** | 0.273 | 0.412 | 0.539 | 0.598 |  |
| **Raven** | 0.426 | 0.577 | 0.770 | 0.800 |  |
| **Wtdbg2** | 0.327 | 0.425 | 0.511 | 0.451 |  |
| **Canu** | 57.000 | 28.000 | 24.000 | 16.000 | **Misassembly count** |
| **Flye** | 132.000 | 105.000 | 109.000 | 93.000 |  |
| **Miniasm** | 93.000 | 89.000 | 52.000 | 45.000 |  |
| **Raven** | 182.000 | 148.000 | 86.000 | 53.000 |  |
| **Wtdbg2** | 348.000 | 315.000 | 294.000 | 280.000 |  |
| **Canu** | 96.100 | 96.300 | 96.100 | 95.200 | **Gene  identification (% complete  BUSCOs)** |
| **Flye** | 96.200 | 96.600 | 96.500 | 96.600 |  |
| **Miniasm** | 96.000 | 95.500 | 95.100 | 93.700 |  |
| **Raven** | 95.700 | 95.900 | 95.700 | 94.800 |  |
| **Wtdbg2** | 94.100 | 92.400 | 95.100 | 93.200 |  |

**Supplementary Table S24:** Evaluation results for the PacBio HiFi real read assemblies.

| **PacBio HiFi real reads assemblies** | | | | | |
| --- | --- | --- | --- | --- | --- |
|  | **S. cerevisiae** | **P. falciparum** | **A. thaliana** | **D. ananassae** |  |
| **Canu** | 0.957 | 0.994 | 0.996 | 0.984 | **Sequence  identity** |
| **Flye** | 0.954 | 0.993 | 0.989 | 0.914 |  |
| **Hifiasm** | 0.954 | 0.994 | 0.981 | 0.972 |  |
| **LJA** | 0.954 | 0.989 | 0.996 | 0.860 |  |
| **MBG** | 0.953 | 0.940 | 0.984 | 0.936 |  |
| **Canu** | 1.795 | 1.052 | 1.149 | 1.216 | **Repeat collapse** |
| **Flye** | 1.051 | 1.022 | 1.021 | 1.049 |  |
| **Hifiasm** | 1.090 | 1.027 | 1.069 | 1.070 |  |
| **LJA** | 1.441 | 1.022 | 1.058 | 1.042 |  |
| **MBG** | 1.337 | 1.684 | 1.067 | 1.088 |  |
| **Canu** | 1.045 | 1.015 | 1.020 | 1.017 | **Rate of  valid sequences** |
| **Flye** | 1.001 | 1.014 | 1.009 | 1.019 |  |
| **Hifiasm** | 1.019 | 1.015 | 0.969 | 1.019 |  |
| **LJA** | 1.021 | 1.012 | 1.015 | 1.010 |  |
| **MBG** | 0.996 | 1.002 | 0.991 | 1.005 |  |
| **Canu** | 0.326 | 0.934 | 0.273 | 0.205 | **Contiguity (NG50 / N50)** |
| **Flye** | 0.265 | 0.836 | 0.227 | 0.151 |  |
| **Hifiasm** | 0.267 | 0.876 | 0.317 | 0.270 |  |
| **LJA** | 0.253 | 0.480 | 0.322 | 0.248 |  |
| **MBG** | 0.082 | 0.006 | 0.008 | 0.007 |  |
| **Canu** | 263.000 | 47.000 | 121.000 | 1894.000 | **Misassembly count** |
| **Flye** | 133.000 | 21.000 | 62.000 | 816.000 |  |
| **Hifiasm** | 149.000 | 31.000 | 105.000 | 1446.000 |  |
| **LJA** | 201.000 | 24.000 | 60.000 | 314.000 |  |
| **MBG** | 158.000 | 25.000 | 62.000 | 1327.000 |  |
| **Canu** | 99.600 | 98.600 | 99.100 | 99.100 | **Gene  identification (% complete  BUSCOs)** |
| **Flye** | 99.600 | 98.500 | 99.200 | 99.100 |  |
| **Hifiasm** | 99.600 | 98.700 | 97.300 | 99.200 |  |
| **LJA** | 99.600 | 98.100 | 99.200 | 99.000 |  |
| **MBG** | 96.700 | 79.900 | 98.600 | 98.500 |  |

Supplementary Table S25-S31 are provided in a separate Excel sheet.
