# Supplementary material for: Evaluating long-read de novo assembly tools for eukaryotic genomes: insights and considerations
Source: Gigascience. 2023 Nov 24;12:giad100. doi: 10.1093/gigascience/giad100 (PMC10673639; doi:10.1093/gigascience/giad100)
Supplement: giad100_Response_to_Reviewer_Comments_Original_Submission [file giad100_response_to_reviewer_comments_original_submission.pdf]

Author's Response To Reviewer Comments

Dear Editor,

In order to be able to use different colors in our responses, we have uploaded a document named Response_letter.docx which includes the reviewers comments and our responses.
