# Supplementary material for: Evaluating long-read de novo assembly tools for eukaryotic genomes: insights and considerations
Source: Gigascience. 2023 Nov 24;12:giad100. doi: 10.1093/gigascience/giad100 (PMC10673639; doi:10.1093/gigascience/giad100)
Supplement: giad100_Reviewer_1_Report_Original_Submission — Brandon Pickett -- 2/23/2023 Reviewed [file giad100_reviewer_1_report_original_submission.pdf]

Reviewer Report

Title: Evaluating Long Read De Novo Assembly Tools for Eukaryotic Genomes: Insights and Considerations

Version: Original Submission **Date:** 2/23/2023

Reviewer name: Brandon Pickett

Reviewer Comments to Author:

Overall, this manuscript is well-written and understandable. There's a lot of good work here and I think the authors were thoughtful about how to compare the resulting assemblies. Scripts and models used have been made available for free via GitHub and could be mirrored on or moved to GigaDB if required. I'll include a several minor comments, including some line-item edits, but the bulk of my comments will focus on a few major items.
Major Comments:
My primary concern here is that the comparison is outdated and doesn't address some of the most helpful questions. CLR-only assemblies are no longer state-of-the-art. There are still applications and situations where ONT (simplex, older-pore)-only assemblies are reasonable, but most projects that are serious about generating excellent assemblies as references are unlikely to take that approach. Generating assemblies for non-reference situations, especially when the sequencing is done "in the field" (e.g., using a MinION with a laptop) or by a group with insufficient funding or other access to PromethIONs and Sequel/Revios, is an exception to this for ONT-only assemblies. Further, this work assumes a person wants to generate "squashed" assemblies instead of haplotype-resolved or pseudohaplotype assemblies. To be fair, sequencing technology in the TGS space has been advancing so rapidly that it is extremely difficult to keep up, and a sequencing run is often outdated by the time analyses are finished, not to mention by the time a manuscript is written, reviewed, and published. Accordingly, in raising my concerns, I am not objecting to the analysis being published or suggesting that the work performed was poor, but I do believe clarifications and discussion are necessary to contextualize the comparison and specify what is missing.
1. This comparison seeks to address Third-generation sequencing technologies: namely PacBio vs. ONT. However, each company offers multiple kinds of long-read sequencing, and they are not all comparable in the same way. Just as long noisy reads (PacBio CLR & ONT simplex) are a whole new generation from "NGS" short reads like from Illumina, long-accurate reads are arguably a new generation beyond noisy long reads. If this paper wants to include PacBio HiFi reads in the comparison, significant changes are necessary to make the comparison meaningful. I think it's reasonable to drop HiFi reads from this paper altogether and focus on noisy long reads since the existing comparison isn't currently set up to tell us enough about HiFi reads and including them would be an ordeal. If including HiFi, consider the following:
1.a. Use assemblers designed for long-accurate reads. HiCanu (i.e., Canu with --pacbio-hifi option) is already used, as is a similar approach for Flye and wtdbg2. However, raven is not meant for HiFi data and miniasm is not either (though, it could be done with the correct minimap2 settings, but Hifiasm

would be better). Assemblies of HiFi data with Raven and miniasm should be removed. Sidenote - Raven can be run with --weaken (or similar) for HiFi data, but it is only experimental and the parameter has since been removed. Including Hifiasm would be necessary, and it should have been included since Hifiasm was out when this analysis was done. Similarly, including MBG (released before your analysis was done) would be appropriate. Since you'd be redoing the analyses, it would be appropriate to include other assemblers that have since been released: namely LJA. Once could argue that Verkko should be included, but that opens another can of worms as a hybrid assembler (more on that later).
1b. Use a read simulator that is built for HiFi reads. Badreads is not built for HiFi data (though using custom parameters to make it work for HiFi reads wasn't a bad idea at the time), and new simulators (e.g., PBSIM3, DOI: 10.1093/nargab/lqac092) have since been released that consider the multi-pass process used to generate HiFi data.
1c. ONT Duplex data is likely not available for the species you've chosen as it is a _very_ new technology. However, you should at least discuss its existence as something for readers to "keep an eye on" as something that is conceptually comparable to HiFi.
1d. Use the latest & greatest HiFi data if possible and at least discuss the evolution of HiFi data. Even better would be to compare HiFi data over time, but this data may not really be available and most people won't be using older HiFi data. Though, simulation of older data would conceivably be possible. While doing so would make this paper more complete, I would argue that it isn't worth the effort at this juncture. For reference, in my observation, older data has a median read length around 10-15 kb instead of 18-22 kb.
1e. Include real Hifi data for the species you are assembling. If none is available and you aren't in a position to generate it, then keep the hifi assembler comparison on real data separate from that of the CLR/ONT assembler comparisons on real data by using real HiFi data for other species.
2. Discuss in the intro and/or discussion that you are focusing on "squashed" assemblies. Without clever sample separation and/or trio-based approaches (e.g., DOI: 10.1038/nbt.4277), a single squashed haplotype is the only possible outcome for PacBio CLR and ONT-only approaches. For non-haploid genomes, other approaches (HiFi-only or hybrid approaches (e.g., HiFi + ONT or HiFi + Hi-C)) can generate pseudohaplotypes at worse and fully-resolved haplotypes at best. The latter is an objectively better option when possible, and it's important to note that this comparison wouldn't apply when planning a project with such goals. Similarly, it would probably be helpful to point out to the novice reader that this comparison doesn't apply to metagenome assembly either.
3. The title suggests to the reader that we'll be shown how long reads makes a difference in assembly compared to non-long read approaches. However, this is not the case, despite some mention of it in near line 318. Short read assemblies are not compared here and no discussion is provided to suggest how long read-based assemblies would improve outcomes in various situations relative to short reads. Unless such a comparison and/or discussion is added, I think the title should be changed. I've included this point in the "Major Comments" section because including such a comparison would be a big overhaul, but I don't expect this to be done. The core concern is that the analysis is portrayed correctly.
4. Sequencing technologies are often portrayed as static through time, but this is not accurate. This is a failing of the field generally. Part of the problem is the length of the publishing cycle (often >1yr from when a paper is written to when it's published, not to mention how long it takes to do the analysis before a paper is even written). Part of the problem is that current statistics are often cited in influential

papers and then recited in more recent papers based on the influential paper despite changes having been made since that influential paper was released. Accordingly, the error rate in ONT reads has been misreported as being ~15% for many years even though their chemistry has improved over time and the machine learning models (especially for human samples) have also improved, dropping the error rate substantially. ONT has made improvements to their chemistry and changed nanopores over time and PacBio has tinkered with their polymerase and chemistry too. Accordingly, a better question for a person planning to perform an assembly would be "which assembler is best for my datatype (pacbio clr vs ont) and chemistry/etc.?" instead of just differentiating by company. Any comparison of those datatypes should at least address this as a factor in their discussion, if not directly in their analysis. I feel that this is missing from this comparison. In an ideal world, we'd have various CLR chemistries and ONT pores/etc. for each species in this analysis. That data likely doesn't exist for each of the chosen species though, and generating it would be non-trivial, especially retroactively. Using the most recent versions is a good option, but may also not exist for every species chosen. Since this analysis was started (circa Nov/Dec 2021 by my estimate based on the chosen assembler versions), ONT has released pore 10; in combination with the most recent release of Guppy, error rates <=3% are expected for a huge portion of the data. That type of data is likely to assemble very differently from R9.4, and starker differences would be expected for data older than R9.4. Even if all the data were the most recent (or from the same generation (e.g., R9.4)), library preps vary greatly, especially between UL (ultralong) libraries and non-UL libraries. Having reads >100kb, especially a large number of them, makes a big difference in assembly outcome in my observation. How does choice of assembler (and possibly different parameters) affect the assembly when UL data is included? How is that different from non-UL data? What about UL data at different percentages of the reads being considered UL? A paper focusing on long noisy reads would be much more impactful if it addresses these questions. Again, this may not be possible for this particular paper considering what's already been done and the available funding, and I think that's okay. However, these issues need to addressed in the discussion as open questions and suggested future work. The type of CLR and ONT data also needs to be specified in this work, e.g., in a supplemental table, and if the various datasets are not from the same types, these differences need to be acknowledged. At a minimum, I think the following data points should be included: chemistry/pore information (e.g., R9.4 for ONT or P2/C5 for PacBio), basecaller (e.g., guppy vX.Y.Z), and read length distribution info (e.g., mean, st. dev., median, %>100kb), ideally a plot of the distribution in addition to summary values. I also understand that these data were generated previously by others, and this information should theoretically be available from their original publications, which are hopefully accessible via the INSDC records associated with the provided accessions. The objective here is making the information easily accessible to the readers of this paper because those could be confounding variables in the analysis.
5. This comparison considered only a single coverage level (30x). That's not an unreasonable shortcut, but it certainly leaves a lot of room for differences between assemblers. If the objective the paper is to help future project planners decide what assembler to use, it would be most helpful if they had an idea of what coverage they can use and still succeed. That's especially true for projects that don't have a lot of funding or aren't planning to make a near-perfect reference genome (which would likely spend the money on high coverage of multiple datatypes). It would be helpful to include some discussion about how these results may be different at much lower (e.g., 2x or 10x coverage) or at higher coverage (e.g., 50x, 70x, etc.) and/or provide some justification from another study for why including that kind of

comparison would be unlikely to be worthwhile for this study, even if project planners should consider those factors when developing their budget and objectives.

6. Figure 2 and 3 include a lot of information, and I generally like how they look and that they provide a quick overview. I believe two things are missing that will improve either the assessment or the presentation of the information, and I think one change will also improve things.

6a. I think metrics from Merqury (DOI: 10.1186/s13059-020-02134-9) should be included where possible. Specifically, the k-mer completeness (recovery rate) and reference-free QV estimate (#s 1 and 3 from https://github.com/marbl/merqury/wiki/2.-Overall-k-mer-evaluation). Generally these are meant to be done from data of the same individual. However, most of the species selected for this comparison are highly homozygous strains that should have Illumina data available, and thus having the data come from not the exact some individual will likely be okay. This can serve as another source of validation. If such a dataset is not available for 1 or more of these species, then specify in the text that it wasn't available, and thus such an evaluation wasn't possible. If it's not possible to add one or both of these metrics to the figures (2 & 3), that's fine, but having it as a separate figure would still be helpful. I find these values to be some of the most informative for the quality of an assembly.

6b. It's not strictly necessary, so this might be more of a minor comment, but I found that I wanted to view individual plots for each metric. Perhaps including such plots in the supplement would help (e.g., 6 sets of plots similar to figure 4 with color based on assembler, grouping based on species, and opacity based on datatype). The specifics aren't critical, I just found it hard to get more than a very general idea from the main figures and wanted something easy to digest for each metric.

6c. Using N50/NG50 for a measure of contiguity is an outdated and often misleading approach. Unfortunately, it's become such common practice that many people feel obligated to include it or use it. Instead, the auN (auNG) would be a better choice for contiguity: https://lh3.github.io/2020/04/08/a-new-metric-on-assembly-contiguity.

7. This paper focuses on assembly and intentionally does not consider polishing (line 176), which I think is a reasonable choice. It also does not consider scaffolding or hybrid assembly approaches (again, reasonable choices). In the case of hybrid assembly options, most weren't available when this analysis was done (short read + long read assemblers were available, but I think it's perfectly reasonable to not have included those). Given the frequency of scaffolding (especially with Hi-C data [DOIs: 10.1371/journal.pcbi.1007273 & 10.1093/bioinformatics/btac808]) and the recent shift to hybrid assemblers (e.g., phasing HiFi-based string graphs using Hi-C data to get haplotype resolved diploid assemblies (albeit with some switch errors) [DOI: 10.1038/s41587-022-01261-x] or resolving HiFi-based minimizer de bruijn graphs using ONT data and parental Illumina data to get complete, T2T diploid assemblies [DOI: 10.1038/s41587-023-01662-6]), I think it would be appropriate to briefly mention these methods so the novice reader will know that this benchmark does not apply to hybrid approaches or post-assembly genome finishing. This is a minor change, but I included it in this section because it matches the general theme of ensuring the scope of this benchmark is clear.

Minor Comments:

1. line 25 in the abstract. Change Redbean to wtdbg2 for consistency with the rest of the manuscript.
2. "de novo" should be italicized. It is done correctly in some places but not in others.
3. line 64. "all TGS technologies": I would argue that this isn't quite true. ONT Duplex isn't included here even though Duplex likely didn't exist when you did this work. Also, see the major comments concerning

whether TGS should include HiFi and Duplex.

4. Table 1. Read length distributions vary dramatically by technology and library prep. E.g., HiFi is often a very tight distribution about the mean because of size selection. Including the median in the table would be helpful, but more importantly, I would like to see read-length distribution plots in the supplement for (a) the real data used to generate the initial iteration models and (b) the real data from each species.
5. line 166 "fair comparison". I'm not sure that a fair comparison should be the goal, but having them at the same coverage level makes them more comparable which is helpful. Maybe rephrase to indicate that keeping them at the same coverage level reduces potentially confounding variables when comparing between the real and simulated datasets.
6. line 169. Citation 18 is used for Canu, which is appropriate but incomplete. The citation for HiCanu should also be included here: DOI: 10.1101/gr.263566.120.
7. line 169. State that these were the most current releases of the various assemblers at the time that this analysis was started. Presumably, that was Nov/Dec 2021. Since then, Raven has gone from v1.7.0->1.8.1 and Flye has gone from v2.9->2.9.1.
8. line 175. Table S6 is mentioned here, but S5 has not yet been mentioned. S5 is mentioned for the first time on line 196. These two supp tables' numbers should be swapped.
9. There is inconsistent use of the Oxford comma. I noticed is missing multiple times, e.g., lines 191, 208, 259, & 342.
10. line 193. The comma at the end of the line (after "tools") should be removed. Alternatively, keep the comma but add a subject to the next clause to make it an independent clause (e.g., "...assembly tools, and they were computed...").
11. line 237. The N50 of the reference is being used here. You provide accessions for the references used, but most people will not go look those up (which is reasonable). The sequences in a reference can vary greatly in their lengths, even within the same species, because which sequences are included in the reference are not standardized. Even the size difference between a homogametic and heterogametic reference can be non-trivial. Which are included in the reference, and more importantly included in your N50 value, can significantly change the outcome and may bias results if these are not done consistently between the included species. It would be helpful if here or somewhere (e.g., in some supplemental text or a table) the contents of these references was somehow summarized. In addition to 1 copy of each of the expected autosomes, were any of the following included: (a) one or two sex chromosomes if applicable, (b) mitochondrial, chloroplast, or other organelle sequences, (c) alternate sequences (i.e., another copy of an allele of some sequence included elsewhere), (d) unplaced sequence from the 1st copy, (e) unplaced sequence from subsequent copies, and (f) vectors (e.g., EBV used when transforming a cell line)?
12. Supplemental tables. Some cells are uncolored, and other cells are colored red or blue with varying shading. I didn't notice a legend or description of what the coloring and shading was supposed to mean. Please include this either with each table or at the beginning of the supplemental section that includes these tables and state that it applies to all tables #-#.
13. Supplemental table S3. It was not clear to me that you created your own model for the hifi data (pacbio_hifi_human2022). I was really confused when I couldn't find that model in the GitHub repo for Badreads. In the caption for this table or in the text somewhere, please make it more explicit that you created this yourself instead of using an existing model.

Methods

Are the methods appropriate to the aims of the study, are they well described, and are necessary controls included? Choose an item.

Conclusions

Are the conclusions adequately supported by the data shown? Choose an item.

Reporting Standards

Does the manuscript adhere to the journal's guidelines on minimum standards of reporting? Choose an item.

Choose an item.

Statistics

Are you able to assess all statistics in the manuscript, including the appropriateness of statistical tests used? Choose an item.

Quality of Written English

Please indicate the quality of language in the manuscript: Choose an item.

Declaration of Competing Interests

Please complete a declaration of competing interests, considering the following questions:

- Have you in the past five years received reimbursements, fees, funding, or salary from an organisation that may in any way gain or lose financially from the publication of this manuscript, either now or in the future?
- Do you hold any stocks or shares in an organisation that may in any way gain or lose financially from the publication of this manuscript, either now or in the future?
- Do you hold or are you currently applying for any patents relating to the content of the manuscript?
- Have you received reimbursements, fees, funding, or salary from an organization that holds or has applied for patents relating to the content of the manuscript?
- Do you have any other financial competing interests?
- Do you have any non-financial competing interests in relation to this paper?

If you can answer no to all of the above, write 'I declare that I have no competing interests' below. If your reply is yes to any, please give details below.

No to 1-5. Maybe to #6, depending on your view. The lab I'm in developed one of the software packages being tested (before I arrived and it is no longer in development). Our lab also recently developed a new assembler, though I would argue that it is in a different ballpark from those tested here.

I agree to the open peer review policy of the journal. I understand that my name will be included on my report to the authors and, if the manuscript is accepted for publication, my named report including any attachments I upload will be posted on the website along with the authors' responses. I agree for my report to be made available under an Open Access Creative Commons CC-BY license (http://creativecommons.org/licenses/by/4.0/). I understand that any comments which I do not wish to be included in my named report can be included as confidential comments to the editors, which will not be published.

Choose an item.

To further support our reviewers, we have joined with Publons, where you can gain additional credit to further highlight your hard work (see: https://publons.com/journal/530/gigascience). On publication of this paper, your review will be automatically added to Publons, you can then choose whether or not to claim your Publons credit. I understand this statement.

Yes Choose an item.
