# Supplementary material for: Evaluating long-read de novo assembly tools for eukaryotic genomes: insights and considerations
Source: Gigascience. 2023 Nov 24;12:giad100. doi: 10.1093/gigascience/giad100 (PMC10673639; doi:10.1093/gigascience/giad100)
Supplement: giad100_Reviewer_2_Report_Original_Submission — Luca Ermini -- 2/28/2023 Reviewed [file giad100_reviewer_2_report_original_submission.pdf]

Reviewer Report

Title: Evaluating Long Read De Novo Assembly Tools for Eukaryotic Genomes: Insights and Considerations

Version: Original Submission **Date:** 2/28/2023

Reviewer name: Luca Ermini

Reviewer Comments to Author:

Dear Cosma and colleagues,
Your manuscript, which I had the pleasure of reading, is, simply put, a benchmark of five long read de novo assembly tools.
Using 13 real and 72 simulated datasets, the manuscript evaluated the performance of five widely used long-read de novo assemblers: Canu, Flye, Miniasm, Raven, and Redbean.
Although I find the methodological approach of the manuscript to be solid and trustworthy, I do not think the research is particularly innovative. Long-read assemblers have already been benchmarked in the scientific literature, and similar findings have been made.
The authors are aware of this limitation of the study and have added a novel feature: the impact of read length on assembly quality, which in my opinion is still lacking sufficient innovation. However, the manuscript as a whole is valid and worthy of consideration. In light of this, I would like to share some suggestions I made in an effort to make the manuscript unique and more novel.
Please see my comment below.
1) Evaluation of the assemblies
The metrics used to evaluate an assembly are frequently a murky subject as we are still lacking a standard language. The authors assessed the assemblies using three types of metrics: compass analysis, assembly statistics, and the Busco assessment, in addition to computational metrics like runtime and RAM usage. This is not incorrect, but I would suggest making a clear distinction between the metrics using (in addition to the computational metrics) three widely recognised metrics, or in short, the 3C criterion. The assembly metrics can be broken down into three dimensions: correctness (your compass analysis), contiguity (NG50) and completeness (the BUSCO assessment). The authors should reconsider the text using the 3C criterion; this will provide a clear, understandable, and structured way of categorising metrics. The paragraph beginning at line 197, for example, causes some confusion for the reader. The NG50 metrics evaluate assembly contiguity, whereas the number of misassemblies (considered by the authors in terms of relocation, inversion, and translocation) evaluate assembly correctness. I must admit that the misassemblies and contiguity can overlap, but I would still recommend keeping the NG50 (within contiguity) and misassemblies (within correctness) metrics separate.
2) Novelty of the comparison
The authors of the study had two main goals: to conduct a systematic comparison of five long-read assembly tools (Raven, Flye, Wtdbg2 or Redbean, Canu, and Miniasm) and to determine whether increased read length has a positive effect on overall assembly quality.

The authors acknowledge the study's limitations and include an evaluation of the effect of read length on assembly quality as a novel feature of the manuscript (see line 70).
The manuscript that described the Raven assembler (Vaser, R., Sikic, M. Time- and memory-efficient genome assembly with Raven. Nat Comput Sci 1, 332-336 (2021)) compared the same assemblers' tools (Raven, Flye, Wtdbg2 or Redbean, Canu and Miniasm) evaluated in this manuscript plus two more (Ra and Shasta), used similar eukaryotes (A. thaliana, D. melanogaster, and Human), and reached a similar conclusion on Flye in terms of contiguity (NG50), and completeness (genome fraction) but overall there is not a best assembler in all of the evaluated categories. In this manuscript authors increased the number of eukaryotic genomes (including S. cerevisiae, C. elegans, T. rupribes, and P. falciparum) and reached similar conclusions: there is no assembler that performs the best in all the evaluation categories, but overall Flye is the best-performing assembler. This strengthens the manuscript, but the research is not entirely novel.
Given that the field of third-generation technologies is rapidly progressing toward the generation of high-quality reads (Pacbio HiFi technology and ONT Q20+ chemistry are achieving accuracy of 99% and higher), the manuscript should also include a HiFi assembler benchmark. This would add novelty to the manuscript and pique the scientific community's interest.
The authors have already simulated HiFi reads from S. cerevisiae, P. falciparum, C. elegans, A. thaliana, D. melanogaster, T. rubripes in addition to reference reads (or real reads) from
S. cerevisiae (SRR18210286). P. falciparum (SRR13050273) and A. thaliana (SRR14728885). Furthermore, I am not sure what the benefit is of evaluating Canu on HiFi data instead of HiCanu, which was designed to deal with HiFi data. The authors already included some HiFi-enabled assemblers like Flye and Wtdbg2 but also HiFiasm should also be considered.
I would strongly advise benchmarking the HiFi assemblers to complete the study and add a level of novelty. I would like to emphasise that the manuscript is solid and that I appreciate it; however, I believe that some novelty should be added.
3) C elegans genomics
The now-discontinued RSII, which had a higher error rate and a shorter average read than Sequel I or Sequel II, was used to generate the genomic data from C elegans. I understand the authors' motivation for including it in the analysis, but the use of RSII may skew the comparisons, and I would suggest adding a few sentences to the discussion about it.
4) CPU time (h) and memory usage
The authors claim the benchmark evaluation included runtime and RAM usage. However, I missed finding information about the runtime and RAM usage.
Please provide CPU time (h) and memory usage (GB)

Minor comments:
----
1) Lines 64-65
"Here, we provide a comprehensive comparison on de novo assembly tools on all TGS technologies and 7 different eukaryotic genomes, to complement the study of Wick and Holt"
I would modify "on all TGS technologies" as "at the present the two main TGS technologies"
2) Line 163

Real reads. The term "real reads" may cause confusion for readers, leading them to believe that the authors produced the sequencing reads for the manuscript. I would use the term "ref-reads" indicating "reads from the reference genomes"

3) Lines 218-219

Please provide full names (genus + species):

S. cerevisiae, P. falciparum, A. thaliana, D. melanogaster, C. elegans, and T. rubripes

4) Supplementary Table S4

"Accession number SRR15720446 seems to belong to a sample sequenced with 1 PACBIO_SMRT (Sequel II) rather than ONT

5) Figures 2 and 3.

Figures 2 and 3 give visual results of the performance of the five assemblers.

I want to make a few points here:

According to what I understand, the top-performing assembler is marked with a star and is plotted with a brighter colour than the others. However, this is not immediately apparent, and some readers might have trouble identifying the colour that has been highlighted. I would suggest either lessening the intensity of the other, lower-performance assemblers or giving the best assembler a graphically distinct outline.

I also wonder if it would be useful to give the exact numbers as supplemental tables.

Methods

Are the methods appropriate to the aims of the study, are they well described, and are necessary controls included? Choose an item.

Conclusions

Are the conclusions adequately supported by the data shown? Choose an item.

Reporting Standards

Does the manuscript adhere to the journal's guidelines on minimum standards of reporting? Choose an item.

Choose an item.

Statistics

Are you able to assess all statistics in the manuscript, including the appropriateness of statistical tests used? Choose an item.

Quality of Written English

Please indicate the quality of language in the manuscript: Choose an item.

Declaration of Competing Interests

Please complete a declaration of competing interests, considering the following questions:

- Have you in the past five years received reimbursements, fees, funding, or salary from an organisation that may in any way gain or lose financially from the publication of this manuscript, either now or in the future?
- Do you hold any stocks or shares in an organisation that may in any way gain or lose financially from the publication of this manuscript, either now or in the future?
- Do you hold or are you currently applying for any patents relating to the content of the manuscript?
- Have you received reimbursements, fees, funding, or salary from an organization that holds or has applied for patents relating to the content of the manuscript?
- Do you have any other financial competing interests?
- Do you have any non-financial competing interests in relation to this paper?

If you can answer no to all of the above, write 'I declare that I have no competing interests' below. If your reply is yes to any, please give details below.

I declare that I have no competing interests

I agree to the open peer review policy of the journal. I understand that my name will be included on my report to the authors and, if the manuscript is accepted for publication, my named report including any attachments I upload will be posted on the website along with the authors' responses. I agree for my report to be made available under an Open Access Creative Commons CC-BY license (http://creativecommons.org/licenses/by/4.0/). I understand that any comments which I do not wish to be included in my named report can be included as confidential comments to the editors, which will not be published.

Choose an item.

To further support our reviewers, we have joined with Publons, where you can gain additional credit to further highlight your hard work (see: https://publons.com/journal/530/gigascience). On publication of this paper, your review will be automatically added to Publons, you can then choose whether or not to claim your Publons credit. I understand this statement.

Yes Choose an item.
